# f-Element heavy pnictogen chemistry

**DOI:** 10.1039/d3sc05056d

**Published:** 2023-12-04

**Authors:** Jingzhen Du, Philip J. Cobb, Junru Ding, David P. Mills, Stephen T. Liddle

**Affiliations:** a College of Chemistry, Zhengzhou University Zhengzhou 450001 China; b Department of Chemistry and Centre for Radiochemistry Research, The University of Manchester Oxford Road Manchester M13 9PL UK david.mills@manchester.ac.uk steve.liddle@manchester.ac.uk

## Abstract

The coordination and organometallic chemistry of the f-elements, that is group 3, lanthanide, and actinide ions, supported by nitrogen ligands, *e.g.* amides, imides, and nitrides, has become well developed over many decades. In contrast, the corresponding f-element chemisty with the heavier pnictogen analogues phosphorus, arsenic, antimony, and bismuth has remained significantly underdeveloped, due largely to a lack of suitable synthetic methodologies and also the inherent hard(f-element)–soft(heavier pnictogen) acid–base mismatch, but has begun to flourish in recent years. Here, we review complexes containing chemical bonds between the f-elements and heavy pnictogens from phosphorus to bismuth that spans five decades of endeavour. We focus on complexes whose identity has been unambiguously established by structural authentication by single-crystal X-ray diffraction with respect to their synthesis, characterisation, bonding, and reactivity, in order to provide a representative overview of this burgeoning area. By highlighting that much has been achieved but that there is still much to do this review aims to inspire, focus and guide future efforts in this area.

## Abstract 2

In this review, the molecular chemistry of the f-elements with heavy pnictogen ligands from phosphorus to bismuth is described.

## Graphical abstract

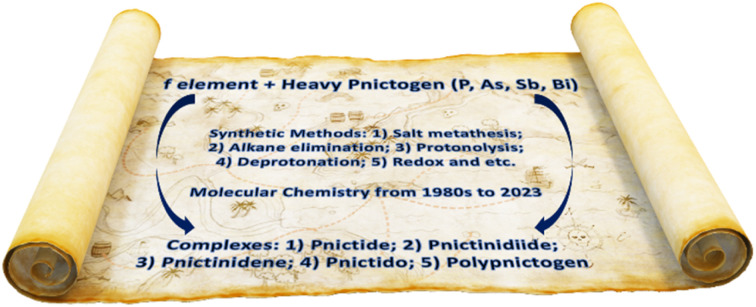

## 1. Introduction

**Fig. 1:**
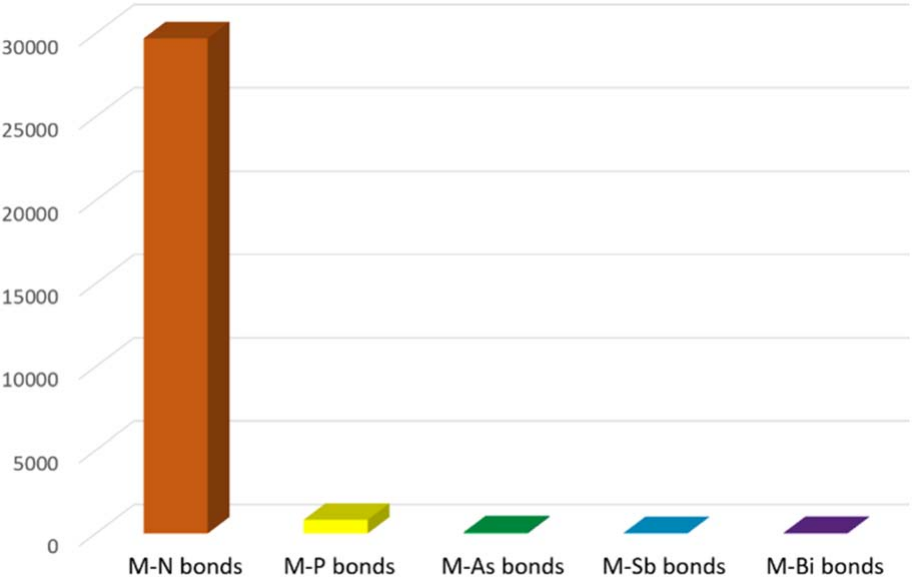
Bar graph summarising the number of molecular structures deposited into the CCDC by 10-08-2023 for any type of f-element pnictogen bond. Total numbers: M–N = 29 713, M–P = 817, M–As = 67, M–Sb = 21, M–Bi = 28.

Due to their widespread and important applications in magnetic materials,^1,2^ electronic devices,^3,4^ bioimaging,^5,6^ synthesis,^7,8^ catalysis,^9,10^ materials science^11,12^ and nuclear technologies,^13,14^ there has been burgeoning interest in the fundamental chemistry of the f-elements over the past few decades. Since f-element metal ions, that is group 3, lanthanide, and actinide ions, are hard Lewis acids with typically large radii and high coordination numbers, they preferentially bind with hard bases (by the hard–soft-acid-base definition); the chemical bonds of these ions are understood to be predominantly ionic, thus their solution chemistry is dominated by N-, O-, and halide-donor ligands.^15^ With ever-developing synthetic methods and characterisation techniques, molecular non-aqueous f-element chemistry has developed in recent years, and under non-aqueous conditions f-element complexes with novel linkages involving softer donor atoms can be accessed and investigated.^16^ More peripherally, but still relevant, it is known that softer donor ligands can effect better selectivity in extraction processes, so the study of such linkages can provide bonding benchmarks of wider relevance.^17^ For group 15, the pnictogens, f-element chemistry is well developed for nitrogen ligands, *e.g.* amides, imides, and nitrides, but this is not the case for the heavier congeners phosphorus, arsenic, antimony, and bismuth.^18–20^ To illustrate the point, a search of the Cambridge Structural Database (CSD, 10th August 2023††Shortly after the census date of this review, reports of a yttrium-bismolyl complex and a yttrium-bismuth cluster were published by Demir and co-workers, and an account of rare-earth phosphinidene complexes was published by Chen and co-workers. See ref. 156–158 for details.)^21^ for any type of crystallographically characterised chemical bond between the f-elements and any pnictogen reveals a stark picture, Fig. 1. There are almost thirty thousand complexes with f-element nitrogen bonds, over 30 times the total number of 933 for f-element heavier pnictogen complexes. Furthermore, below phosphorus there are only 67, 21, and 28 examples of f-element bonds to arsenic, antimony, and bismuth, respectively, with most of those examples reported in the last decade.

The above data parallel transition metal chemistry in many regards, though are a more extreme picture reflecting that stabilisation of the heavier and softer pnictogen ligands multiply bonded at large and hard Lewis acidic f-element metal ions is certainly more challenging. However, f-element pnictinidene and pnictido complexes remain of interest since in addition to being heavy amide, imide, and nitride analogues, they are isoelectronic congeners of transition metal alkyls, carbenes, and carbynes, respectively, that have developed into excellent catalysts for various organic transformations^22^ or as precursors to inorganic materials.^23^ Furthermore, whilst f-element phosphorus and arsenic multiple bonding is precedented, f-element antimony or bismuth multiple bonds are conspicuous by their absence.^20^ Indeed, as group 15 is descended the pnictide ions become increasingly electropositive and metal-like, which increases the challenges of pairing electropositive f-element and increasingly large pnictogen metal ions together to form weak and highly polarised metal–metal bonds. Thus, well-defined molecules are of vital importance to study the inherent physicochemical properties and nature of covalency in f-element ligand bonds. This in turn could benefit the development of new synthetic methods, ligand design, catalytic transformations, and materials precursors. Reflecting the growing nature of this field, there have been a number of excellent but very general or ligand-specific review articles and book chapters covering the historical developments of some of the subtopics,^20,24–29^ but recent developments justify a broad but detailed review specifically focussed on this topic.

This review highlights the most notable achievements in the field of f-element heavy pnictogen chemistry from phosphorus to the heaviest abundant main group element bismuth up to August 2023. In line with the criteria for reviews, a representative selection, rather than a complete literature survey, is presented, and discussions concentrate on structurally characterised molecules. We aim to highlight the major advances involving all heavy pnictogen ligand types, with the exception of phospholyl and arsolyl ligands, which were reviewed in 2021,^30^ and (OCE)^−^ (E = P, As) ligands, which were reviewed in 2019 and are normally O-bound unless the E centre decisively directs the chemistry;^31^ several other previous reviews have separately covered the ligand classes that comprise this review.^20,24–29^ Here we present current challenges to inspire researchers and focus and guide future efforts of the field to develop f-element heavy pnictogen chemistry more rapidly in the future. In this review, we include the group 3 elements scandium, yttrium, and lanthanum under the heading of lanthanide sections for convenience. ^31^P NMR chemical shifts for P-bound complexes covered in this review are compiled in Table 1.

**Table 1:** ^31^P NMR chemical shifts of reported f-element complexes with phosphorus ligands in this review

Complex name	^31^P NMR (ppm)	Solvent	Ref.
[Y(Cp′′)_2_(THF)(PHSi^*t*^Bu_3_)] (5)	−181.1	C_6_D_6_	44
[Y(Cp′′)_2_{(μ-PH_2_)(μ-Li[tmeda])}_2_(Cl)}] (6)	−218.5	Toluene-*d*_8_	45
[Yb(Cp*)_2_{(PCHCMeCMeCHC)_2_}] (8c)	191.6	THF-*d*_8_	48
[Yb(Cp*)_2_{(PCHCMeCMeCHC)_2_}] (8c)	178.2	Toluene-*d*_8_	48
[{Lu(PNP^iPr^)(μ-PMes)}_2_] (9)	186.8	C_6_D_6_	50
[{Lu(PNP^iPr^)(μ-PMes)}_2_] (9)	18.1	C_6_D_6_	50
[{Nd(μ-PDipp)(I)(THF)_3_}_2_] (10)	−168	C_6_D_6_	51
[{Sc(PNP^iPr^)(μ-PTripp)}_2_] (13)	227.4	C_6_D_6_	53
[{Sc(PNP^iPr^)(μ-PTripp)}_2_] (13)	7.0	C_6_D_6_	53
[Sc(PNP^iPr^)(μ-PDmp)(μ-Br)Li] (14)	9.8	C_6_D_6_	53
[Sc(PNP^iPr^)(μ-PDmp)(μ-Br)Li] (14)	8.0/13.2	C_6_D_6_	53
[Sc(PNP^iPr^)(μ-PDmp)(μ-Br)Li(DME)] (15)	56.1	C_6_D_6_	53
[Sc(PNP^iPr^)(μ-PDmp)(μ-Br)Li(DME)] (15)	10.8/5.6	C_6_D_6_	53
[{Sc(NCCN^iPr^)(μ-PXyl}_2_] (16)	183.8	C_6_D_6_	54
[{Sc(NCCN^iPr^)(μ-PXyl)(DMAP)}_2_] (17)	181.3	C_6_D_6_	54
[Sc(NCCN^iPr^)(2,2′-bipy){η^2^-P_2_(Xyl)_2_}] (18)	30.0/25.9	C_6_D_6_	54
[{Sc(NCCN^iPr^)}_2_(μ-S){μ-η^2^-P_2_(Xyl)_2_}] (19S)	−79.0	C_6_D_6_	54
[{Sc(NCCN^iPr^)}_2_(μ-Se){μ-η^2^-P_2_(Xyl)_2_}] (19Se)	−72.2	C_6_D_6_	54
[{Sc(NCCNiPr)}_2_(μ-Te){μ-η^2^-P_2_(Xyl)_2_}] (19Te)	−60.1	C_6_D_6_	54
[Sc(NCCN^Dipp^)(Me){P(H)Dipp}] (20)	−90.9	C_6_D_6_	55
[{Sc(NCCN^Dipp^)}_2_(μ-CH_2_)(μ-PDipp)] (21)	84.1	C_6_D_6_	55
[{Y[PhC(NDipp)_2_](μ_2_-Me)}_3_(μ_3_-Me)(μ_3_-PPh)] (26Y)	138.8	C_6_D_6_	56
[{Lu[PhC(NDipp)_2_](μ_2_-Me)}_3_(μ_3_-Me)(μ_3_-PPh)] (26Lu)	103.4	C_6_D_6_	56
[{Y[PhC(NDipp)_2_]}_3_(μ_2_-Me)_2_(μ_3_-Me)(μ_2_,η^2^:η^3^-PC_6_H_4_)] (27Y)	262.48	C_6_D_6_	56
[{Lu[PhC(NDipp)_2_]}_3_(μ_2_-Me)_2_(μ_3_-Me)(μ_2_,η^2^:η^3^-PC_6_H_4_)] (27Lu)	192.52	C_6_D_6_	56
[{Y(Cp^Me^)_2_}_3_(μ-PMes)_3_Li][Li(THF)_4_]_2_ (28Y)	57.24	C_6_D_6_	58
[Sc(NCCN^Dipp^){PP(EDA^Dipp^)}] (29)	412.0	C_6_D_6_	59
[Sc(NCCN^Dipp^){PP(EDA^Dipp^)}] (29)	157.2	C_6_D_6_	59
[Sc(NCCN^Dipp^){PP(EDA^Dipp^)}] (29)	402.3	THF-*d*_8_	59
[Sc(NCCN^Dipp^){PP(EDA^Dipp^)}] (29)	158.5	THF-*d*_8_	59
[Sc(NCCN^Me^){PP(EDA^Dipp^)}] (30)	324.8	C_6_D_6_	59
[Sc(NCCN^Me^){PP(EDA^Dipp^)}] (30)	169.0	C_6_D_6_	59
[Sc(NCCN^Me^){PP(EDA^Dipp^)}] (30)	312.2	THF-*d*_8_	59
[Sc(NCCN^Me^){PP(EDA^Dipp^)}] (30)	166.8	THF-*d*_8_	59
[Sc(NCCN^Me^){(μ-PB)[N(Dipp)CHCHN(Dipp)]}(μ-Cl)K]_2_ (35)	11.4	Toluene-*d*_8_	60
[Sc(NCCN^Me^){(μ-PB)[N(Dipp)CHCHN(Dipp)]}(μ-Cl){K(DB18C6)}] (36)	19.6	Toluene-*d*_8_	60
[Sc(NCCN^Me^){N(^i^Pr)C(PB{N(Dipp)CHCHN(Dipp)})N(^i^Pr)}] (37)	−103.1	C_6_D_6_	60
[Y(Tp^*t*Bu,Me^)(Me)(HPDipp)] (38Y)	−117.8	C_6_D_6_	61
[Y(Tp^*t*Bu,Me^)(PDipp)(DMAP)_2_] (40)	−5.5	C_6_D_6_	61
[{Y(I)}{Y[μ_3_-P(Dipp)](μ-I)(THF)}_4_(μ_6_-P){K(C_7_H_8_)}] (41)	347.4	C_6_D_6_	62
[{Y(I)}{Y[μ_3_-P(Dipp)](μ-I)(THF)}_4_(μ_6_-P){K(C_7_H_8_)}] (41)	154.7	C_6_D_6_	62
[{Y(I)}{Y[μ_3_-P(Dipp)](μ-I)(THF)}_4_(μ_6_-P){K(THF)}] (42)	346.6	C_6_D_6_	62
[{Y(I)}{Y[μ_3_-P(Dipp)](μ-I)(THF)}_4_(μ_6_-P){K(THF)}] (42)	154.3	C_6_D_6_	62
[{Y(I)}{Y(I)(THF)}_2_{Y(THF)_2_}_2_(μ-I)[μ_3_-P(Dipp)]_4_(μ_5_-P)] (43)	358.8	THF-*d*_8_	62
[{Y(I)}{Y(I)(THF)}_2_{Y(THF)_2_}_2_(μ-I)[μ_3_-P(Dipp)]_4_(μ_5_-P)] (43)	148.4	THF-*d*_8_	62
[{Y(I)}{Y(I)(THF)}_2_{Y(NCCN^iPr^)}_2_(μ-I)[μ_3_-P(Dipp)]_4_(μ_5_-P)] (44)	400.7	THF-*d*_8_	62
[{Y(I)}{Y(I)(THF)}_2_{Y(NCCN^iPr^)}_2_(μ-I)[μ_3_-P(Dipp)]_4_(μ_5_-P)] (44)	176.3/172.9	THF-*d*_8_	62
[{Y(THF)}{Y(μ-I)(THF)}_2_{Y(THF)}{Y(Cp*)}(μ_3_-I)(μ-I)[μ_3_-P(Dipp)]_4_(μ_6_-P){K(THF)}] (45)	300.9	THF-*d*_8_	62
[{Y(THF)}{Y(μ-I)(THF)}_2_{Y(THF)}{Y(Cp*)}(μ_3_-I)(μ-I)[μ_3_-P(Dipp)]_4_(μ_6_-P){K(THF)}] (45)	72.8 to 130.4	THF-*d*_8_	62
[{Sc(NN^fc^)}_3_P_7_] (47Sc)	−131.4 to 23.2	C_6_D_6_	64
[{Y(NN^fc^)(THF)}_3_P_7_] (47Y)	−130.4 to −20.4	C_6_D_6_	64
[{Sm(DippForm)_2_}_2_(μ^2^-η^4^:η^4^-P_4_)] (48)	453	C_6_D_6_	67
[{Y(DippDBD)(THF)}_2_(P_3_)][K(18C6)(toluene)] (49)	−273.01	THF-*d*_8_	68
[Th(Cp*)_2_(PPh_2_)_2_] (50)	143	C_6_D_6_	79
[Th(Cp*)_2_(μ-PPh_2_)_2_Ni(CO)_2_] (51)	177	C_6_D_6_	79
[Th(Cp*)_2_(μ-PPh_2_)_2_Pt(PMe_3_)] (52)	149.3	Toluene-*d*_8_	80
[Th(Cp*)_2_(μ-PPh_2_)_2_Pt(PMe_3_)] (52)	−3.3	Toluene-*d*_8_	80
[Th(Cp*)_2_(PHTripp)_2_] (53)	1.66	C_6_D_6_	81
[Th(Tren^TIPS^)(PH_2_)] (54Th)	−144.08	C_6_D_6_	83
[U(Tren^TIPS^)(PH_2_)] (54U)	595.07	C_6_D_6_	82
[Th(Tren^TCHS^)(PH_2_)] (55Th)	−133.01	THF-*d*_8_	84
[U(Tren^TCHS^)(PH_2_)] (55U)	605.91	THF-*d*_8_	84
[{Th(Cp*)_2_}_2_{μ-P[(2,6-CH_2_CHCH_3_)_2_-4-^i^PrC_6_H_2_]}] (57)	161.9	C_6_D_6_	81
[{Th(Cp*)_2_(μ-PTripp)(μ-PHTripp)(K)}_2_] (58)	171.91	THF-*d*_8_	87
[{Th(Cp*)_2_(μ-PTripp)(μ-PHTripp)(K)}_2_] (58)	−110.54	THF-*d*_8_	87
[Th(Cp*)_2_(PTripp)}(PHTripp)][K(2,2,2-cryptand)] (59)	177.86	THF-*d*_8_	87
[Th(Cp*)_2_(PTripp)}(PHTripp)][K(2,2,2-cryptand)] (59)	−106.99	THF-*d*_8_	87
[{Th(Cp^tt^)_2_(PMes*}(ClK)}_2_](60)	108.79	C_6_D_6_	93
[Th(Cp^tt^)_2_(PMes*)(μ-Cl){K(18C6)}] (61)	133.5	C_6_D_6_	93
[U(Cp*)_2_(PMes*)(OPMe_3_)] (62)	71.06	C_6_D_6_	95
[U(Cp*)_2_(PMes*)(OPMe_3_)] (62)	−59.84	C_6_D_6_	95
[Th(Cp^ttt^)_2_(PMes*)] (63)	145.7	C_6_D_6_	97
[{U(Tren^TIPS^)(μ-PH)}{K(2,2,2-cryptand)}] (66)	2460.4	C_6_D_6_	82
[Th(Tren^TIPS^)(PH)][Na(12C4)_2_] (67)	198.8	C_6_D_6_	83
[Th(Tren^TCHS^)(PH)][Na(2,2,2-cryptand)] (69Th)	266.16	THF-*d*_8_	84
[U(Tren^TCHS^)(PH)][Na(2,2,2-cryptand)] (69U)	2628.50	THF-*d*_8_	84
[{Th(Tren^TIPS^)}_2_(μ-P)][Na(12C4)_2_] (70)	553.5	THF-*d*_8_	83
[{Th(Tren^TIPS^)}_2_(μ-PH)] (71Th)	145.7	C_6_D_6_	83
[Th(Tren^TIPS^)(OCP)] (74Th)	−339.91	C_6_D_6_	110
[U(Tren^TIPS^)(OCP)] (74U)	−319.96	C_6_D_6_	109
[{Th(Tren^TIPS^) }_6_(μ-OC_2_P_3_)_2_(μ-OC_2_P_3_H)_2_Rb_4_] (76)	217.99 to 261.14	C_6_D_6_	110
[{Th(Cp^tt^)_2_}_2_(μ^2^-η^4^-P_6_)] (77)	−41.9 to 125.3	Toluene-*d*_8_	111
[{Th(Cp^tt^)_2_}(μ^2^-η^3^-P_3_){Th(Cp^tt^)_2_Cl}] (78)	−94.5 to −69.7	CD_2_Cl_2_	111
[{U(Cp*)(C_8_H_6_(Si^i^Pr_3_)_2_)}_2_(μ^2^-η^4^-P_4_)] (79)	718	C_6_D_6_	112
[{Th(Cp′′)_3_}_2_(μ^2^-η^2^-P_4_)] (80)	−246.55 to 323	C_6_D_6_	113

## 2. Nomenclature

**Fig. 2:**
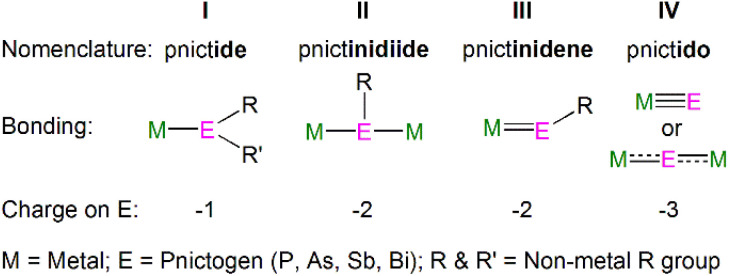
General nomenclature for pnictogen metal bonding.

Metal heavy pnictogen nomenclature depends upon the pnictogen identity, charge and binding mode. The prefix is determined by the pnictogen identity; the general prefix is ‘pnict-’, whilst bonds involving phosphorus, arsenic, antimony and bismuth begin with ‘phosph-’, ‘ars-’, ‘stib-’, and ‘bism-’, respectively. The suffix denotes the charge of the pnictogen and binding mode; the suffix ‘ide’ is used for a terminally bound pnictogen bearing a formal −1 charge, whereas a terminal pnictogen with a −2 charge ends with ‘-inidene’. A bridging pnictogen with a −2 charge has the suffix ‘-inidiide’, and lastly a pnictogen bearing a −3 charge ends with ‘-ido’, independent of the binding mode. This gives the four bonding types: pnictide (I), pnictinidiide (II), pnictinidene (III) and pnictido (IV), Fig. 2. Exceptions to these rules are seen for (As)^3−^ and (R_2_As)^−^ ligands, which are given the prefix ‘arsen-’ to give the respective terms arsenido and arsenide when bound to metal centres. An additional exception is made for the parent phosphide (H_2_P)^−^, which is given the unique moniker ‘phosphanide’. However, within some f-element pnictinidiide and pnictido examples, the ligands can be bridged by more than two metal centres to form more complex bonding modes which are not presented in Fig. 2, but will be discussed with specific examples in the following sections.

## 3. Synthetic methodologies for generating f-element pnictogen bonds

Precise synthetic strategies can vary depending on the type of pnictogen reagents and f-element precursors, but a pnictogen donor ligand is commonly installed on an f-element metal centre in one of the following general ways:

(1) Dative coordination of a neutral phosphorus or arsenic ligand to form an adduct with an f-element complex that has an available vacant coordination site; this tends to not be the case for antimony or bismuth, which need to be negatively charged to coordinate to an f-element metal centre.

(2) Salt elimination/metathesis of alkali metal pnictogen anions with an f-element halide (or halide equivalent) precursor to produce a polarised-covalent f-element pnictogen linkage.

(3) Alkane elimination between a primary or secondary pnictogen precursor and f-element alkyl (or cyclometallate) complex exploiting the acidic nature of the proton on the pnictogen atom.

(4) Oxidising highly reducing low-valent f-elements with pnictogen compounds.

(5) Combining salt and alkane elimination approaches using a primary pnictide alkali metal salt to react with an f-element alkyl and halide starting material (mainly used to produce metal–ligand multiple bonds).

## 4. Lanthanide phosphorus complexes

The past few decades have seen significant progresses in f-element phosphorus chemistry, with many novel f-element phosphorus motifs isolated and investigated.^20,24–29^ The neutral, soft phosphine donor tends to form weak dative bonding interactions to hard f-element metal ions, though this can be overcome by incorporating P-donor centres into polydentate ligands as demonstrated separately by Fryzuk and Lu,^32–34^ and such complexes were reviewed previously, so these compounds are not included here.^20,26,32–34^ This section discusses recent advances in f-element complexes containing phosphide/phosphanide, phosphorin, phosphinidiide/phosphinidene, phosphido, and inorganic polyphosphorus ligands.

### 4.1 Lanthanide phosphide complexes

**Fig. 3:**
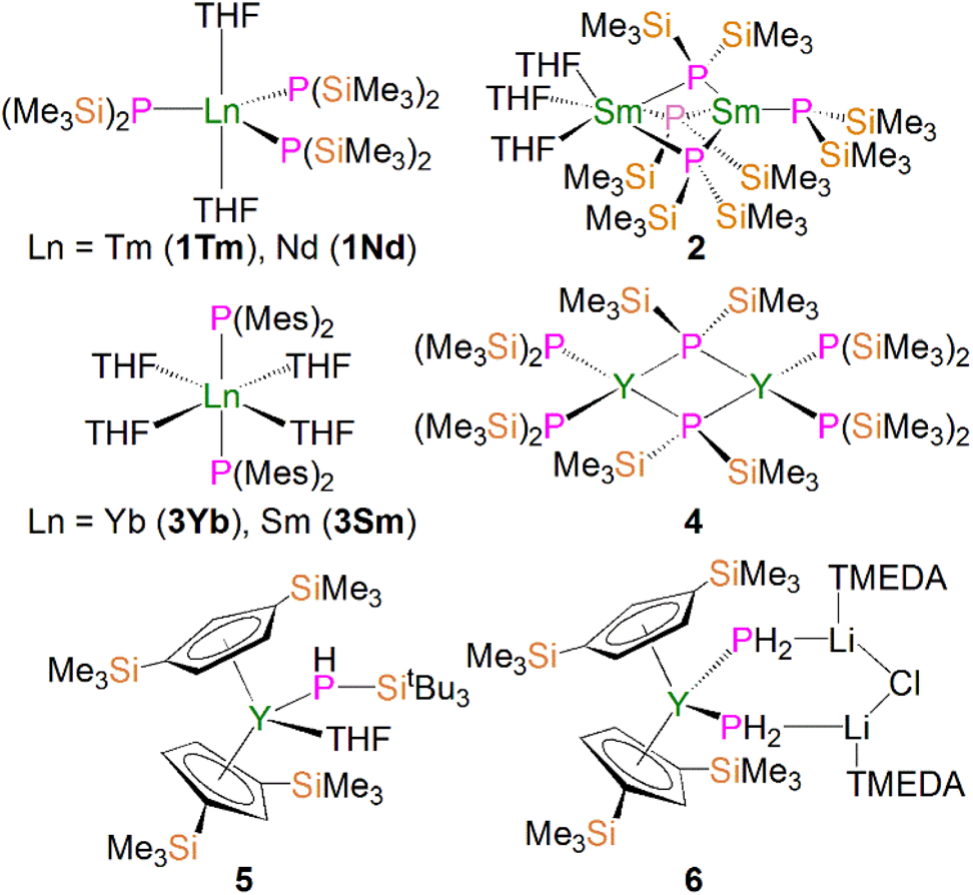
Examples of lanthanide–phosphide complexes 1–6.

Due to the large size of the metal ions, lanthanide phosphide complexes tend to form multi-nuclear species with bridging phosphide ligands; mono-nuclear complexes are therefore relatively rare and usually require bulky stabilising phosphorus substituents as demonstrated by Izod.^35–37^ The first mono-nuclear lanthanide phosphide complex [Tm{P(SiMe_3_)_2_}_3_(THF)_2_] (1Tm) was reported by Rabe and co-workers in 1995, Fig. 3; this was prepared from the reaction of [TmI_3_(THF)_3.5_] with three equivalents of KP(SiMe_3_)_2_ in THF *via* salt elimination.^38^ The molecular structure of 1Tm exhibits the five-coordinate thulium centre in a distorted trigonal bipyramidal geometry with two axial THF molecules and three equatorial bis(trimethylsilyl)phosphide ligands. The Tm–P bond distances of 2.709(1) and 2.701(2) Å are typical of single bonds. Subsequently, the isostructural neodymium analogue [Nd{P(SiMe_3_)_2_}_3_(THF)_2_] (1Nd) was isolated using the same synthetic approach, Fig. 3. The Nd–P bond lengths of 2.80(4) and 2.83(3) Å are slightly longer than those in 1Tm, attributed to the larger metal radii of Nd than Tm in the same coordination environment.^39^ However, because of the paramagnetic metal centres, no resonances were observed in their ^31^P NMR spectra. However, the similar reaction of the divalent samarium precursor [SmI_2_(THF)] with two equivalents of KP(SiMe_3_)_2_ in THF produced dinuclear and asymmetric [Sm{P(SiMe_3_)_2_}{μ-P(SiMe_3_)_2_}_3_Sm(THF)_3_] (2).^40^ Using a similar salt elimination method, Nief and co-workers showed that monomeric divalent lanthanide phosphide complexes could be accessed by isolation of [Ln{P(Mes)_2_}_2_(THF)_4_] (Ln = Yb, 3Yb, Sm, 3Sm; Mes = 2,4,6-Me_3_C_6_H_2_), Fig. 3, where the metal centres adopt octahedral geometries with two axial bis(mesityl)phosphide ligands and four equatorial THF molecules.^41,42^ According to the +2 oxidation state, the Sm–P bond of 3.034(2) Å in 3Sm is significantly longer than those of trivalent 1Ln. In 1997, Rabe and co-workers also reported the synthesis and molecular structures of the first examples of divalent lanthanide phosphide complexes containing primary phosphide ligands, [Ln{HP(Mes*)}_2_(THF)_4_] (Ln = Yb, Eu; Mes* = 2,4,6-^*t*^Bu_3_C_6_H_2_), which also exhibit octahedral metal centres.^43^

In 2002, Westerhausen and co-workers isolated a dinuclear yttrium phosphide complex [Y{P(SiMe_3_)_2_}_2_{μ-P(SiMe_3_)_2_}]_2_ (4) and a mono-nuclear yttrium phosphide complex [Y(Cp′′)_2_(THF)(PHSi^*t*^Bu_3_)] (5, Cp′′ = 1,3-(SiMe_3_)_2_C_5_H_3_), Fig. 3.^44^ The latter contains one primary phosphide ligand stabilised by the sterically demanding Cp′′ ligands paired with the bulky phosphorus substituents. With the convention of using a Cp′′ centroid as a ligating point, the molecular structure of 5 revealed that the metal centre adopts a pseudo-tetrahedral geometry and the Y–P bond distance is 2.770(1) Å. Complex 5 is diamagnetic and the ^31^P NMR spectrum exhibits a doublet of doublets resonance at −181.1 ppm with ^1^*J*_YP_ and ^1^*J*_PH_ values of 144.0 and 201.0 Hz, respectively. Phosphanide complexes featuring the (PH_2_)^−^ ligand are exceedingly rare for f elements. Although actinide phosphanide complexes with terminal An–PH_2_ linkages are known (see below), isostructural analogues still remain rare for lanthanides. The only relevant example is the yttrium complex [Y(Cp′′)_2_{(μ-PH_2_)(μ-Li[tmeda])}_2_(Cl)}] (6), Fig. 3, which contains two (PH_2_)^−^ groups that bridge to two lithium cations.^45^ It was found that 6 is unstable in both solid and solution states, and decomposes under argon atmosphere at room temperature to produce PH_3_ as well as a small amount of H_2_PSiMe_3_, reflecting the synthetic challenge of stabilising terminal lanthanide-PH_2_ species.

### 4.2 Lanthanide phosphorin and biphosphinine complexes

**Fig. 4:**
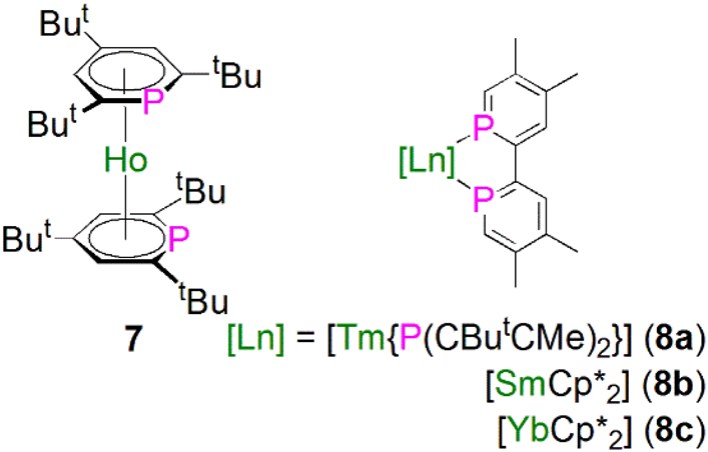
Lanthanide complexes containing phosphorin ligands 7 and 8.

In 1997, Cloke and co-workers reported the synthesis and molecular structure of the bis(2,4,6-tri-*tert*-butyl-phosphorin)holmium(0) complex [Ho(η^6^-Ttp)_2_] (7), Fig. 4, prepared by co-condensation of holmium vapor with an excess of 2,4,6-tri-*tert*-butyl-phosphorin at −196 °C followed by further work-up and recrystallisation.^46^ The remarkable thermostability of 7 (*T*_sublimation_ = 160 °C, 10^−5^ mbar, 90% recovery) arises from the better π-acceptor capability for phosphorin over arenes, which was confirmed by optical and magnetic data for 7. The structure of 7 was found to exhibit extensive disorder, with the P-atoms equally disordered over the three possible positions of each phosphorin ligand, so no preference for *syn* or *anti* conformations could be inferred.

During 2014 to 2016 Nocton, Clavaguéra, and co-workers reported biphosphinine complexes of the general formula [Ln(L)_2_{(PCHCMeCMeCHC)_2_}] (Ln = Tm, L = {P(CBu^*t*^CMe)_2_}, 8a; Ln = Sm or Yb, L = Cp*, C_5_Me_5_, 8b, 8c),^47,48^Fig. 4. In these complexes the biphosphinine ligands are formally radical anions but the extent of electron transfer for the Yb complex was ambiguous, with characterisation data intermediate to closed or fully open shell formulations. The Tm–P, Sm–P, and Yb–P distances were found to be 2.825/2.862(2), 2.909(2)/2.927(2), and 2.872(2)/2.938(2) Å, consistent with the radii of the lanthanide ions. We note that this work followed on from prior work on P-methylated phosphinine ligands with chelating side arms from Arliguie, Mézailles, and co-workers; however, in those complexes the anion charge partially delocalises into the C_5_P rings, resulting in rather long M–P bonds (∼3 Å for Ce, Nd, and U) that are between dative phosphines and covalent phosphides,^49^ thus we do not discuss them further.

### 4.3 Lanthanide phosphinidiide complexes

Unlike their d-transition metal counterparts, lanthanide–pnictogen multiple bonds are relatively rare as a result of the valence orbital spatial and energy mismatch of 4f metal ions and pnictogen ligands. Most lanthanide pnictinidenes form bridging dimeric pnictinidiide complexes where the “Ln=Pn” moiety is stabilised through additional interactions with adjacent rare earth metal centres or electropositive alkali metal cations.^26^ This section describes the progresses made in isolation of a handful of lanthanide pnictinidiide complexes before the terminal phosphinidene species was finally secured very recently.

**Scheme 1:**
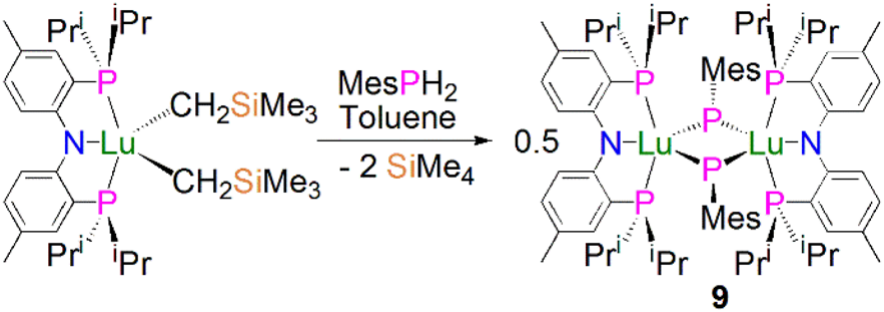
Synthesis of Lu–phosphinidiide complex 9.

In 2008, Kiplinger and co-workers synthesised the first bridging phosphinidiide lanthanide complex [Lu{(PNP^iPr^)(μ-PMes)}_2_] (9, PNP^iPr^ = [{2-(^i^Pr_2_P)C_6_H_4_}_2_N]^−^) by protonolysis of [(PNP^iPr^)Lu(CH_2_SiMe_3_)_2_] with MesPH_2_, in 52% yield, Scheme 1.^50^ Complex 9 exhibits an asymmetric Lu_2_P_2_ core, with two short [2.5973(15)/2.6031(16) Å] and two long [2.6527(16)/2.6724(14) Å] Lu–P bonds. The sum of the angles about the phosphorus atoms range from 358.9 to 356.5°, which indicates that the phosphorus lone pairs possibly π-donate to the Lu ions. The authors concluded that the structural data for 9 suggested that the complex formed *via* the dimerisation of a transient terminal phosphinidene species, [Lu(PNP^iPr^)(PMes)].

**Scheme 2:**
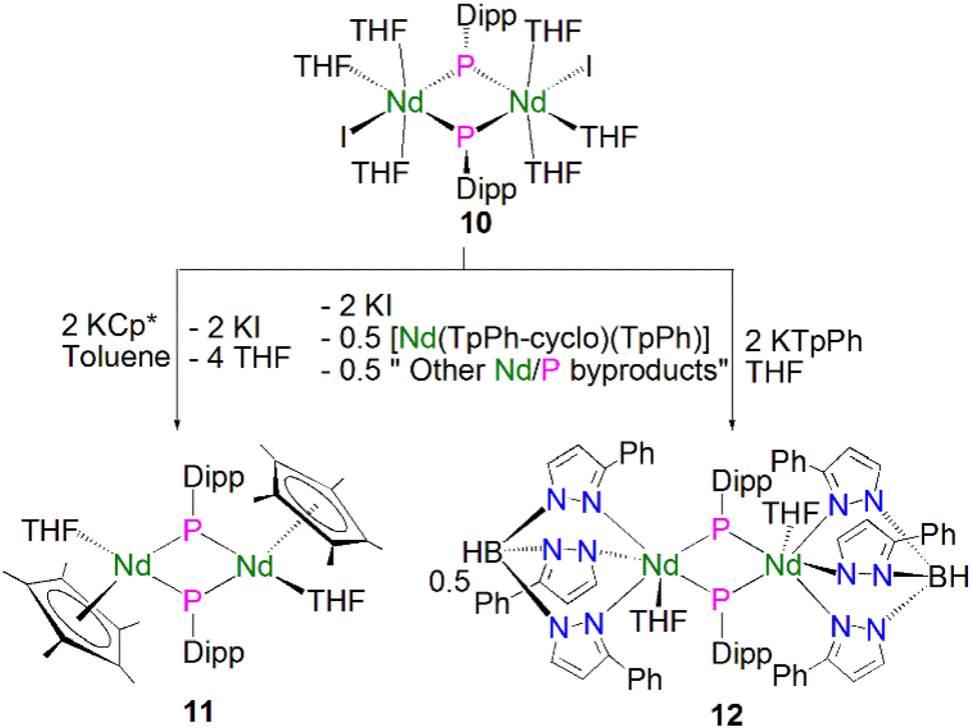
Synthesis of Nd–phosphinidiide complexes 10–12.

Soon afterwards, Chen and co-workers reported the synthesis of the first early lanthanide phosphinidiide complex, [{Nd(I)(μ-PDipp)(THF)_3_}_2_] (10, Dipp = 2,6-^i^Pr_2_C_6_H_3_), *via* the concomitant salt elimination and silyl redistribution reaction of [NdI_3_(THF)_3.5_] with two equivalents of KP(SiMe_3_)(Dipp) to eliminate one equivalent of P(SiMe_3_)_2_(Dipp) and two equivalents of KI, Scheme 2.^51^ The Nd(iii) ions in 10 exhibits pseudo-octahedral geometries, with the two bridging phosphinidiides forming an asymmetric Nd_2_P_2_ core that is analogous to the Ln_2_P_2_ core of 9, with Nd–P bond distances of 2.7314(15) and 2.7769(16) Å. In common with 9, the phosphorus atoms in 10 are trigonal planar, with the sum of bond angles equalling 359.1°. The authors carried out preliminary investigations into the reactivity of 10, establishing that it reacts in a similar fashion to carbenes with substrates such as benzophenone to give a phosphaalkene.

In 2010 Chen and co-workers later expanded the range of neodymium phosphinidiide complexes, utilising 10 in salt metathesis reactions with two equivalents of either KCp* or KTp^Ph^ (KHB(3-Ph-N_2_C_3_H_2_)_3_) to yield the phosphinidiide complexes [{Nd(Cp*)(μ-PDipp)(THF)}_2_] (11) and [{Nd(Tp^Ph^)(μ-PDipp)(THF)}_2_] (12), respectively, Scheme 2.^52^ Complex 11 was isolated as the major product in a yield of 52%. Complex 12, however, was initially isolated as a crystalline mixture with the cyclometallated complex [Nd(Tp^Ph^-*cyclo*)(TpPh)], with purification of the two complexes performed *via* the manual separation of crystals. The solid-state structure of 11 revealed that there is a loss of the trigonal planar geometry of the bridging phosphinidiides, indicated by the decrease of the sum of bond angles of the phosphorus atom to 349.2(3)° when compared to complexes 12 [358.9(4)°] and 10 [359.1°], which could be a result of the coordination of the sterically demanding Cp* ligand. Complex 11 exhibits an analogous Nd_2_P_2_ core to that of 10, with inequivalent Nd–P distances of 2.7456(11) and 2.7827(10) Å, whilst 12 demonstrates a more regular core geometry with Nd–P distances of 2.7808(16) and 2.7911(15) Å. The molecular structure of 12 exhibits one inverted pyrazolyl group on each Tp^Ph^ ligand, which is a result of isomerisation of the ligand *via* a 1,2-shift to relieve steric buttressing between the Tp^Ph^ ligand and the phosphinidiide Dipp group.

**Scheme 3:**
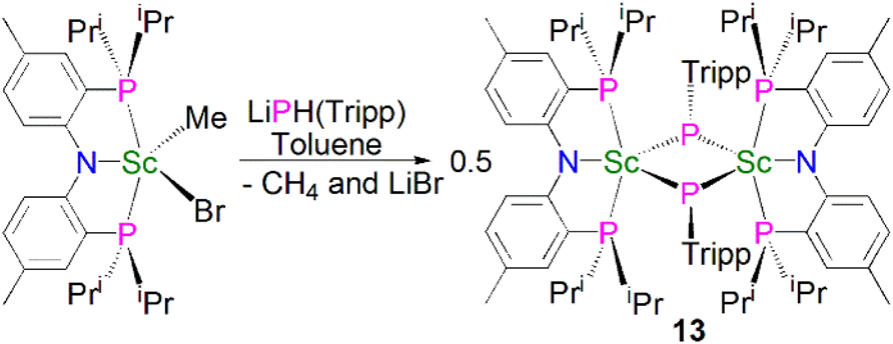
Synthesis of Sc–phosphinidiide complex 13.

In 2010, Mindiola and co-workers reported that the reaction of the sterically demanding phosphide precursor, LiPH(Tripp) (Tripp = 2,4,6-^i^Pr_3_C_6_H_2_), with [Sc(PNP^iPr^)(Me)(Br)] yielded the bridging phosphinidiide dinuclear scandium complex [{Sc(PNP^iPr^)(μ-PTripp)}_2_] (13), Scheme 3, again *via* the elimination of one equivalent each of methane and lithium bromide.^53^ The authors postulated that 13 formed *via* aggregation of a transient terminal scandium phosphinidene species, [Sc(PNP^iPr^)(PTripp)]. The molecular structure of 13 revealed an asymmetric four-membered Sc_2_P_2_ ring core, with similar Sc–P bond distances of 2.5446(8) and 2.5527(10) Å. Based on the crystallographic data for 13 the authors proposed that the phosphorus lone pairs are delocalised around the Sc_2_P_2_ core.

**Scheme 4:**
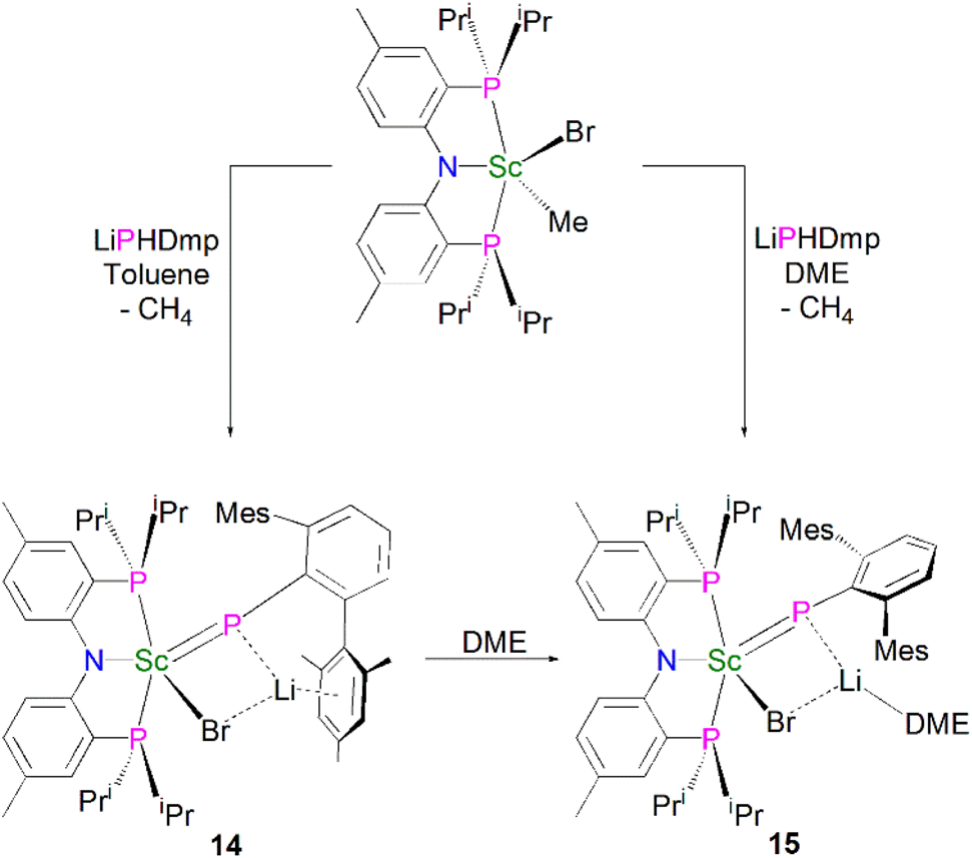
Synthesis of Sc–phosphinidiide complexes 14 and 15.

Mindiola and co-workers subsequently disclosed the first mononuclear rare earth phosphinidiide complexes. The Li-capped complexes [Sc(PNP^iPr^)(μ-PDmp)(μ-Br)Li(DME)_*n*_] (Dmp = 2,6-Mes_2_C_6_H_3_; *n* = 0, 14; *n* = 1, 15) were prepared from the concomitant salt metathesis and protonolysis reactions of the bulky primary phosphide lithium salt, LiPHDmp with [(PNP^iPr^)Sc(Me)(Br)], eliminating methane as the driving force, to give 14 (55%) and 15 (47%), Scheme 4.^53^ The exact product depends on the reaction solvent; complex 15 was also synthesised by the addition of a stoichiometric amount of DME to 14 (21%). Single crystal X-ray diffraction studies revealed that both 14 and 15 have short Sc–P bond lengths of 2.338(2) and 2.3732(18) Å, respectively. Calculations performed on 15 indicated that the Sc=P bond has significant multiple bond character with a Mayer bond order of 1.46. The authors varied the reaction conditions for the synthesis of 14 and 15 to investigate if the elimination of LiBr was possible. However, heating reaction mixtures up to 100 °C did not liberate the occluded LiBr.

**Scheme 5:**
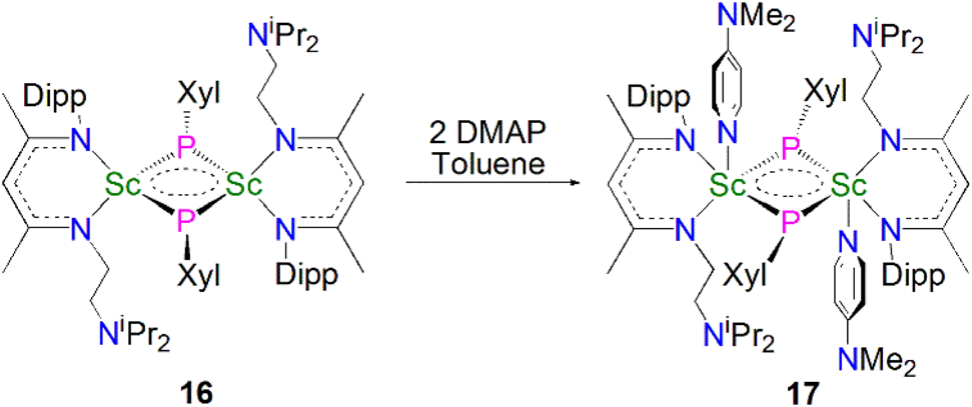
Synthesis of Sc–phosphinidiide complexes 16 and 17.

In 2013, Maron, Chen and co-workers utilised a similar one-pot salt metathesis and protonolysis methodology with the reaction of KPHXyl (Xyl = 2,6-Me_2_C_6_H_3_) with [Sc(NCCN^iPr^)(Me)(Cl)] (NCCN^iPr^ = MeC(NDipp)CHC(Me)(NCH_2_CH_2_N^i^Pr_2_) yielding the bridging phosphinidiide dinuclear scandium complex [{Sc(NCCN^iPr^)(μ-PXyl}_2_] (16, 74%), *via* the elimination of KI and methane, Scheme 5.^54^ In contrast with the previously reported Sc–phosphinidiide complex 13, the Sc_2_P_2_ core in 16 is more symmetric with Sc–P distances of 2.522(1) and 2.528(1) Å. They also attempted to synthesise a terminal Sc–phosphinidene complex by reacting 16 with a strongly donating ligand, DMAP (DMAP = 4-NMe_2_-C_5_H_4_N) to break the dimer up; however, this resulted in the formation of the adduct [{Sc(NCCN^iPr^)(μ-PXyl)(DMAP)}_2_] (17) in a 90% yield. The authors noted that 17 was the major product if an excess of DMAP (four equivalents) was added to 16. Whilst 17 retains its Sc_2_P_2_ core, the Sc–P bonds are longer than 16 at 2.540(1) and 2.589(1) Å, reflecting the increased Sc coordination numbers.

**Scheme 6:**
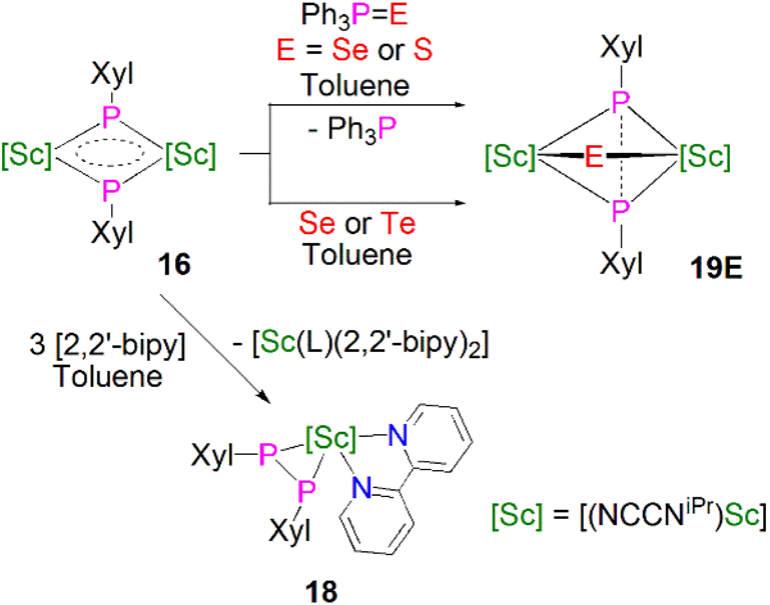
Selected redox reactions of 16.

Maron, Chen and co-workers showed that 16 exhibits notable redox chemistry; for example, the addition of three equivalents of 2,2′-bipyridine (2,2′-bipy) results in the oxidative coupling of two phosphinidiide fragments to form a diphosphanide Sc-complex [Sc(NCCN^iPr^)(2,2′-bipy){η^2^-P_2_(Xyl)_2_}] (18), with the single electron reduction of two molecules of 2,2′-bipy affording [Sc(NCCN^iPr^)(2,2′-bipy·)_2_] as a side product, Scheme 6. Similarly, the oxidative coupling of the two (PXyl)^2−^ ligands to (P_2_(Xyl)_2_)^2−^ promotes the reduction of elemental selenium/tellurium or Ph_3_P=E (E = Se or S) to S^2−^, Se^2−^ or Te^2−^, yielding the bridging chalcogenido complexes [{Sc(NCCN^iPr^)}_2_(μ-E){μ-η^2^-P_2_(Xyl)_2_}] (19E, E = S, Se or Te). The authors also found that 16 readily undergoes nucleophilic addition chemistry with a range of unsaturated allene, nitrile, isocyanide, and CS_2_ substrates to generate the corresponding organophosphorus scandium complexes. In follow-up work they reported that 16 could readily cleave the boron–oxygen bonds in pinacol–borane and catecholborane to give complexes featuring a newly-generated ligand (HB{P(Xyl)}_2_)^2−^, demonstrating the reactive nature of the Sc-phosphinidiide species due to the highly ionic Sc–P bonding interaction.^54^

**Scheme 7:**
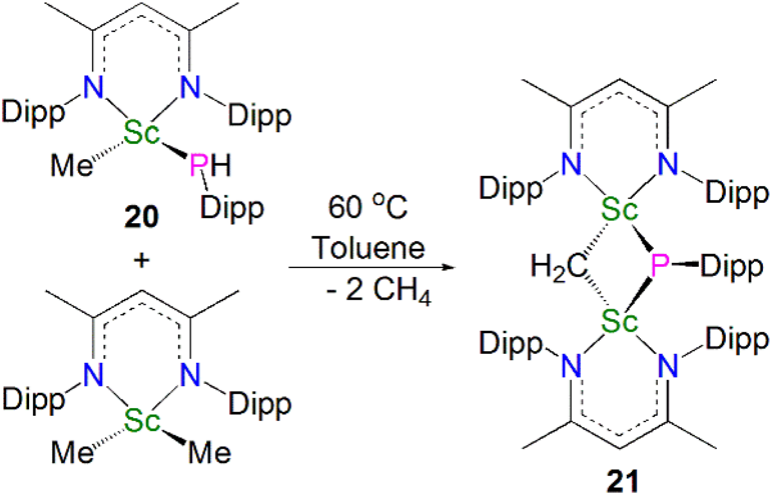
Synthesis of the Sc–phosphinidiide complex 21.

In 2015, Maron, Chen and co-workers found that thermal decomposition of the scandium phosphide precursor [Sc(NCCN^Dipp^)(Me){P(H)Dipp}] (20, NCCN^Dipp^ = {MeC(NDipp)}_2_CH) supported by the NCCN^Dipp^ scaffold afforded the scandium phosphinidiide complex [{(NCCN^Dipp^)Sc}_2_(μ-CH_2_)(μ-PDipp)] (21). However, this reaction also gave a diphosphide by-product [(NCCN^Dipp^){P(H)Dipp}_2_] in a 1 : 1 ratio with 21. An optimal route was then implemented by reacting one equivalent of 20 with [Sc(NCCN^Dipp^)(Me)_2_], giving 21 in a 77% yield, Scheme 7.^55^ Complex 21 exhibits Sc–P bond lengths of 2.495(1) and 2.508(1) Å, which are shorter than other scandium bridging phosphinidiide complexes. Interestingly, there is an up-field shift of the phosphinidiide moiety resonances in the ^31^P NMR spectrum of 21 to 84.1 ppm, *cf.*13 (227.4 ppm) and 16 (183.8 ppm), which is presumably due to one of the bridging phosphinidiide units being replaced by a methylidene fragment.

**Scheme 8:**
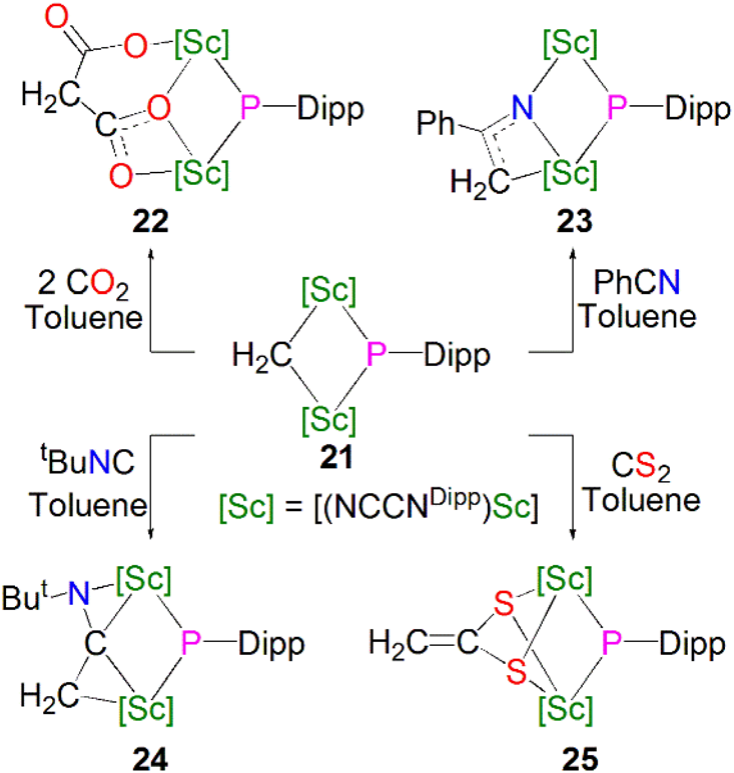
Reactivity of Sc–phosphinidiide complex 21.

In the same publication Maron, Chen and co-workers conducted a reactivity study of 21; this revealed that, in contrast with the previously reported complex 16, the phosphinidiide ligand in 21 is relatively unreactive, with all small molecules reacting at the adjacent methylidene centre, Scheme 8. The reaction of 21 with either CO_2_, PhCN, ^*t*^BuNC or CS_2_ resulted in insertion of the unsaturated functional group into the Sc–CH_2_ bonds to give the bridging phosphinidiide complexes [{Sc(NCCN^Dipp^)}_2_(L){μ-P(Dipp)}] (L = μ-O-COCH_2_CO_2_, 22; μ-N-C(Ph)(CH_2_), 23; μ-C(CH_2_)(N^*t*^Bu), 24; or (μ-S)_2_CCH_2_, 25). Complexes 22–25 all demonstrate similar Sc–P distances between 2.4922(13) and 2.588(2) Å. DFT studies of 21 indicated that the lack of reactivity of the Sc–P bond was possibly due to its increased covalency when compared to the Sc–C bonds in the same complex.^55^

**Scheme 9:**
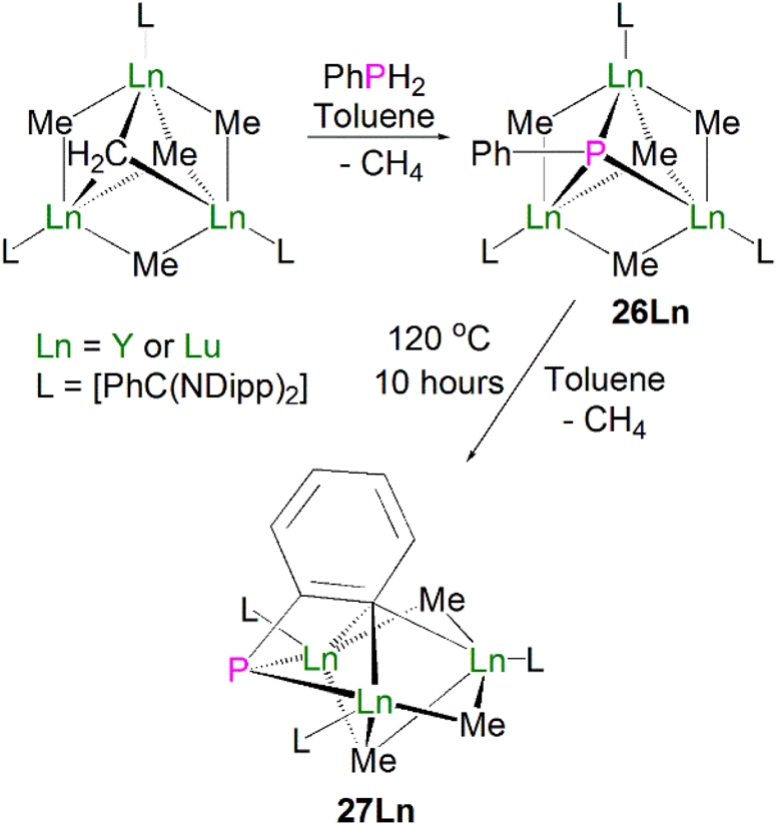
Synthesis of Lu- and Y–phosphinidiide complexes 26Ln–27Ln.

Zhou, Luo, Zhang and co-workers later expanded the number of structurally characterised lanthanide phosphinidiide complexes *via* the protonolysis reactions of [{Ln[PhC(NDipp)_2_](μ_2_-Me)}_3_(μ_3_-Me)(μ_3_-CH_2_)] (Ln = Y, Lu) and one equivalent of PhPH_2_ to give the clusters [{Ln[PhC(NDipp)_2_](μ_2_-Me)}_3_(μ_3_-Me)(μ_3_-PPh)] (Ln = Lu, 26Lu, 92%; Ln = Y, 26Y, 90%), Scheme 9.^56^ These clusters exhibit a rare μ^3^-bridging mode of the phosphinidiide between three rare earth metal ions, which is in contrast to most other rare earth phosphinidiide complexes where a μ^2^-bridging mode is observed. Complex 26Y exhibits one long [2.9432(12) Å] and two short [2.7142(11) and 2.7317(12) Å] Y–P bond lengths. The longer distance is within the range of previously reported dative R_2_P:→Ln(iii) interactions. Once the change in metal radii is accounted for, this rationale can be applied to 26Lu, which exhibits similar asymmetric bonding between the phosphinidiide and three bonded Ln(iii) ions, with Lu–P distances of 2.902(2), 2.684(2) and 2.639(2) Å. Both complexes contain P–C bonds that are bent out of the Ln_3_ plane (Lu: 49.3°; Y: 59.1°); this differs from analogous transition metal bridging phosphinidiide complexes where the P–C bond is approximately perpendicular to the M_3_ plane. In common with other rare earth phosphinidiide complexes, the authors reported that complexes 26Ln exhibited reactivity towards unsaturated molecules such as ketones, thiones, or isothiocyanates, where the complex undergoes phospha–Wittig chemistry, with the exchange of the phosphinidene for an oxo or a sulfido group. Upon heating complexes 26Ln in toluene, an additional molecule of methane was eliminated to yield the μ_2_-bridging phosphinidiide complexes [{Ln[PhC(NDipp)_2_]}_3_(μ_2_-Me)_2_(μ_3_-Me)(μ_2_,η^2^:η^3^-PC_6_H_4_)], 27Ln, in high yields (Ln = Lu, 94%; Y, 91%). Complex 27Lu exhibits Lu–P distances of 2.649(4)/2.644(4) Å, which is a similar range to that of the two shorter Lu–P bonds in 26Lu, whilst 27Y displays a range of Y–P distances [2.698(2)/2.692(2) Å] that are shorter than those seen for 26Y. The authors have additionally reported the crystal structure of the yttrium bridging phosphinidiide complex [{Y(PhC[NDipp]_2_)(μ-Me)}_2_(μ_3_-CCCPh)(μ,η^2^:η^3^-PC_6_H_4_)],^57^ which exhibits Y–P bond distances of 2.701(3) and 2.700(3) Å; these are statistically indistinguishable from the corresponding distances seen in 27Y. However, the synthetic route and additional characterisation data for this complex have not been reported to date.

**Scheme 10:**
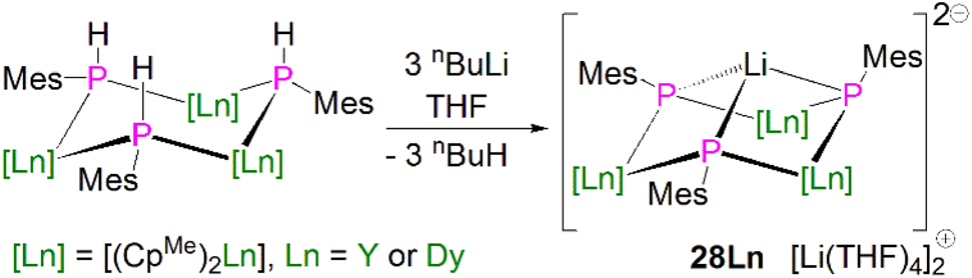
Synthesis of Y- and Dy–phosphinidiide complexes 28Ln.

In 2015, Layfield and co-workers synthesised the bridging phosphinidiide lanthanide complexes [{Ln(Cp^Me^)_2_}_3_(μ-PMes)_3_Li] [Li(THF)_4_]_2_ (28Ln; Ln = Y or Dy; Cp^Me^ = C_5_H_4_Me), Scheme 10.^58^ The deprotonation reactions of the bridging lanthanide phosphides, [{Ln(Cp^Me^)_2_(μ-PHMes}_3_] (Ln = Y or Dy), with three equivalents of *n*-butyl-lithium gave 28Y and 28Dy, in yields of 56% and 64%, respectively, in addition to three equivalents of butane gas. The solid-state structures of 28Ln exhibit central Ln_3_P_3_ cores in a chair-like configuration, and the phosphinidiide units are capped with a single lithium ion. The authors reported longer Y–P bond distances for 28Y [2.7869(12)–2.8268(13) Å] than the yttrium complex 27Y, which is possibly due to the coordination a lithium ion to the three phosphinidiide units. Complex 28Dy exhibits Dy–P bond distances of 2.7850(15)–2.8249(15) Å, which are shorter than the range of distances seen for the phosphide precursor [{Dy(Cp^Me^)_2_(μ-PHMes}_3_] [2.926(6)–2.951(6) Å], which is likely due to increasing negative charge localised at the phosphorous atom resulting in stronger electrostatic bonding.

**Scheme 11:**
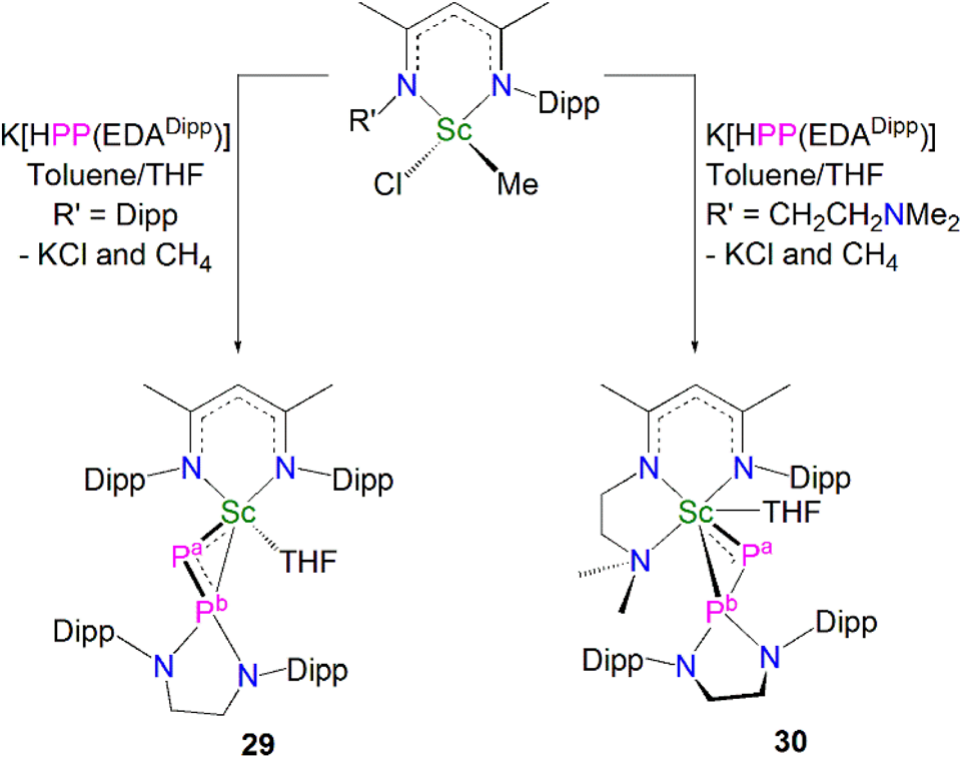
Synthesis of the Sc–phosphinophosphinidene complexes 29 and 30.

In 2020, Maron, Chen and co-workers utilised the phosphinophosphinidene ligand [PP{N(Dipp)CH_2_CH_2_N(Dipp)}]^2−^ (PP{EDA^Dipp^}^2−^) in the one-pot salt metathesis and protonolysis reactions of [Sc(NCCN^R^)(Me)(Cl)] (NCCN^R^ = DippNC(Me)CHC(Me)NR′; R = Me (see 33 below) R′ = CH_2_CH_2_NMe_2_ or Dipp) with one equivalent of K[HPP{EDA^Dipp^}] to afford the Sc phosphinophosphinidene complexes [Sc(NCCN^R^){PP(EDA^Dipp^)}] (R = Dipp, 29, 54%; R = Me, 30, 67%), with elimination of one equivalent each of methane and potassium chloride, Scheme 11.^59^ Due to the poor stability of the K[HPP{EDA^Dipp^}] ligand precursor, it was synthesised *in situ* from the phosphine derivative and benzyl potassium. Complex 29 exhibits a shorter Sc–P phosphinidene bond distance [2.448(1) Å] when compared to 30 [2.484(1) Å], which the authors attributed to the increase in coordination number of the scandium centre from five to six.

**Fig. 5:**
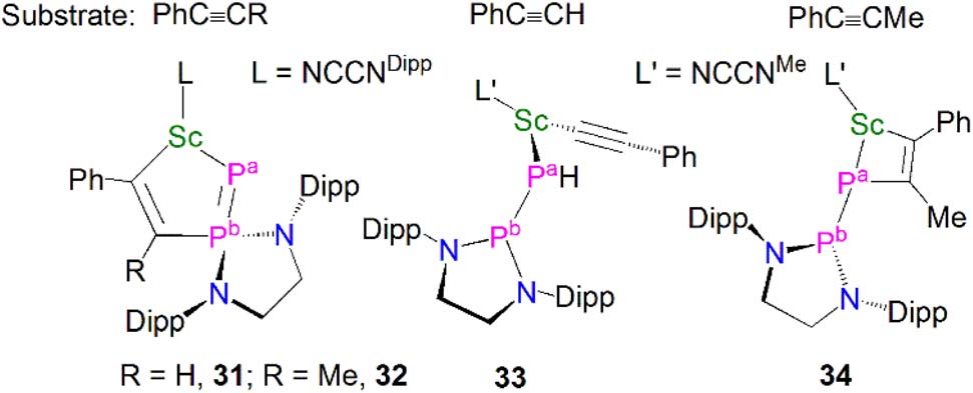
Reactivity of Sc–phosphinophosphinidene complexes 29 and 30 to give 31–34.

Complex 29 displays interesting reactivity towards unsaturated small molecules such as alkynes when compared to 30.^59^ The reaction of 29 with one equivalent of PhC≡CR (R=H or Me) gave [Sc(NCCN^Dipp^){η^2^-P=P(EDA^Dipp^)CR = CPh}], Fig. 5, (R = H, 31; R = Me, 32), which was surprising as reactions with unsaturated molecules typically occur at the more nucleophilic centre, *i.e.* the phosphinidene (P^a^-phosphorus). Indeed, the authors reported that 30 reacts as expected at the phosphinidene centre with the same alkynes PhC≡CR to yield [(NCCN^Me^)Sc{P(H)P(EDA^Dipp^)}(C≡CPh)] (33) and [(NCCN^Me^)Sc{η^2^-PP(EDA^Dipp^)MeC = CPh}] (34). The calculated reaction pathways for 29 and 30 with PhC≡CH illustrated that the interesting reactivity at the P^b^-phosphorus in the case of 29 was a consequence of the coordinated THF blocking the access of reactants to the P^a^-phosphinidene centre.

### 4.4 Lanthanide phosphinidene complexes

**Scheme 12:**
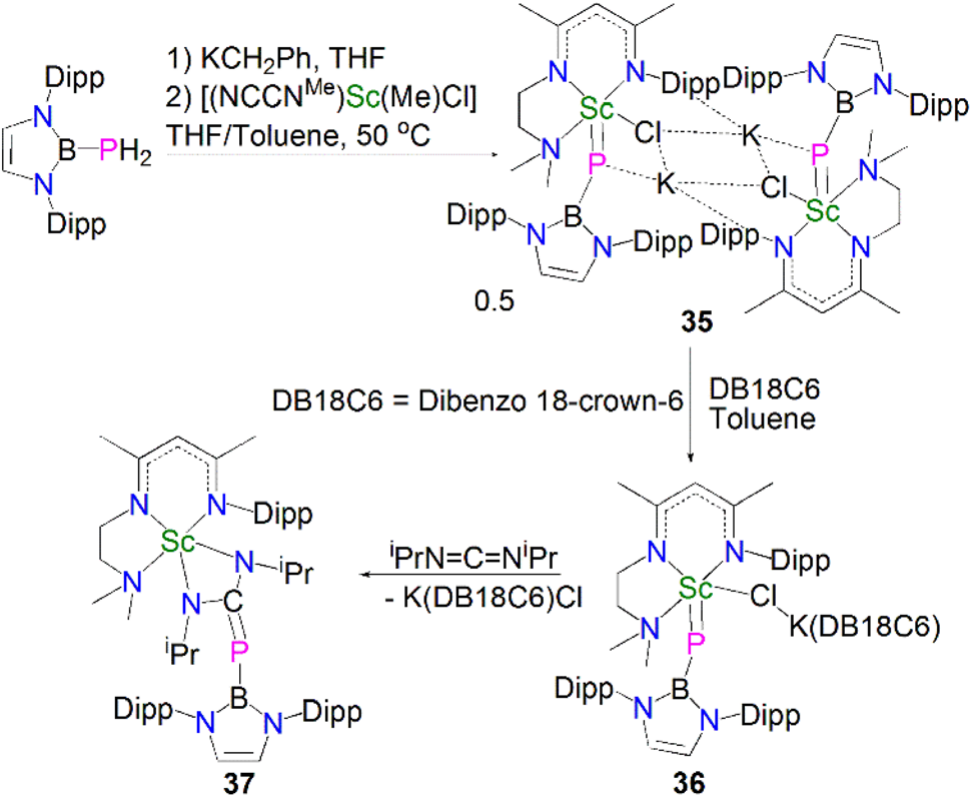
Synthesis and reactivity of a terminal Sc–phosphinidene complexes 36.

Whilst there were many lanthanide metal phosphinidiide complexes reported in the past two decades, a bona fide terminal phosphinidene complex for any lanthanide metal remained elusive for decades. A breakthrough in this field was achieved very recently; in 2021, Maron, Chen and co-workers reported the synthesis and molecular structure of the first terminal scandium phosphinidene complex, Scheme 12.^60^

Building on their Sc–phosphinophosphinidene work, Maron, Chen and co-workers reacted the boronylphosphine H_2_PB{N(Dipp)CHCHN-(Dipp)} with KCH_2_Ph to give *in situ*-generated K[HPB{N(Dipp)CHCHN(Dipp)}], which was treated with [Sc(NCCN^Me^)(Me)(Cl)] in a THF/toluene mixture to afford a K/Sc heterometallic phosphinidiide complex 35 after heating at 50 °C for 24 h. Dimeric 35 could be converted to a terminal monomeric boronylphosphinidene complex 36 as a dark purple solid in 83% yield by reacting with dibenzo-18-crown-6 in toluene. The solid-state structure confirmed the boronylphosphinidene ligand of 36 adopts an end-on coordination even though the chloride ligand is still bridged between the Sc and K metal centres. The Sc–P bond length in 36 (2.381(1) Å) is close to that in 35 (2.397(2) Å) but shorter than that in the scandium phosphinophosphinidene complex 30 (2.484(1) Å). The ^31^P{^1^H} NMR spectrum for 36 at 25 °C shows a very broad signal, but data recorded at −30 °C gave a sharper resonance at 19.6 ppm. DFT studies on 36 revealed a three-centre two-electron (3c-2e) Sc–P–B σ bond with a strong Sc–P π-interaction. In line with the nucleophilic nature of the phosphinidene ligand, a preliminary reactivity study showed that 36 reacted with *N*,*N*′-diisopropylcarbodiimide at room temperature *via* a [2 + 2]-addition fashion to give a four-membered scandium metallaheterocycle complex, [Sc(NCCN^Me^){N(^i^Pr)C(PB{N(Dipp)CHCHN(Dipp)})N(^i^Pr)}] (37) in 82% yield.

**Scheme 13:**
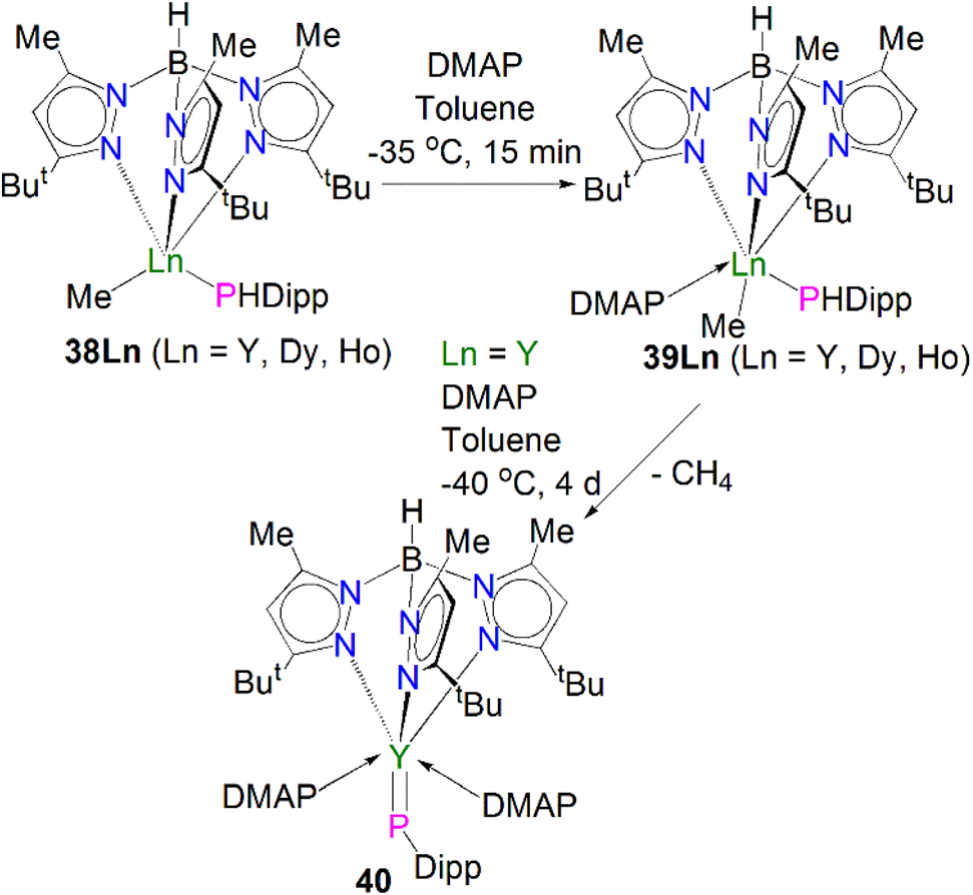
Synthesis of a terminal Y–phosphinidene complex 40.

Very recently, Sirsch, Anwander and co-workers reported the synthesis and molecular structure of the first terminal yttrium phosphinidene complex, Scheme 13.^61^ The reactions of H_2_PDipp with the lanthanide dimethyl complexes [Ln(Tp^*t*Bu,Me^)(Me)_2_] (Ln = Y, Dy, Ho; Tp^*t*Bu,Me^ = HB(2-Me-4-^*t*^Bu-N_2_C_3_H)_3_) afforded the corresponding phosphide complexes [(Tp^*t*Bu,Me^)Ln(Me)(HPDipp)] (38Ln, Ln = Y, Dy, Ho). Addition of DMAP to 38Ln gave the corresponding DMAP adducts [(Tp^*t*Bu,Me^)Ln(Me)(HPDipp)(DMAP)] (39Ln, Ln = Y, Dy, Ho); addition of a further equivalent of DMAP to 38Y produced [Y(Tp^*t*Bu,Me^)(PDipp)(DMAP)_2_] (40). Overall, 40 was prepared *via* a double-deprotonation approach previously established by Anwander for terminal lanthanide imido complexes. However, attempts to synthesise the Dy and Ho phosphinidene analogues using the similar strategy were unsuccessful. Moreover, the Lewis acid-stabilised yttrium phosphinidiide [Y(Tp^*t*Bu,Me^)[(μ-PDipp)(μ-Me)AlMe_2_] was prepared by addition of H_2_PDipp to the solution of [Y(Tp^*t*Bu,Me^)(Me)(AlMe_4_) in toluene. However, cleavage of trimethylaluminum within this phosphinidiide complex to target a terminal phosphinidene was unsuccessful as well. The solid-state structure of 40 revealed a short Y–P distance of 2.4855(7) Å, which is in between the sum of single and double covalent bond radii of Y and P (2.74 and 2.32 Å, respectively), indicating multiple bonding character. The ^31^P NMR spectrum of 40 exhibits a very weak signal at −5.5 ppm with a maximum ^1^*J*_YP_ coupling constant of 282 Hz, which is shifted to lower frequency compared to that of [Y(Tp^*t*Bu,Me^)[(μ-PDipp)(μ-Me)AlMe_2_] at −52.2 ppm, suggesting the increase of electron density at the yttrium centre in 40. A computational study of 40 confirmed the highly polarised covalent multiple bond of Y=PDipp linkage and revealed one σ- and two π-type Y–P interactions with a Wiberg bond index (WBI) of 1.37. The successful isolation and characterisation of terminal phosphinidenes 36 and 40 indicates that it should be possible to access terminal phosphinidene species with other lanthanides with the right supporting ligands combined with suitable phosphinidene group transfer methodologies and reagents.

### 4.5 Lanthanide phosphido complexes

In common with their pnictinidene derivatives, there are relatively few examples of f-element, and hence lanthanide, heavy pnictido complexes. To date the only structurally authenticated complexes to feature f-element pnictido bonding are with phosphorus or arsenic, with the pnictogen bridging between two or more metal centres.^20^ There have been no structurally authenticated f-element stibido or bismuthido derivatives to date, therefore they are highly sought-after synthetic targets. For lanthanide metal complexes, only a few clusters were reported containing phosphido ligand.

**Scheme 14:**
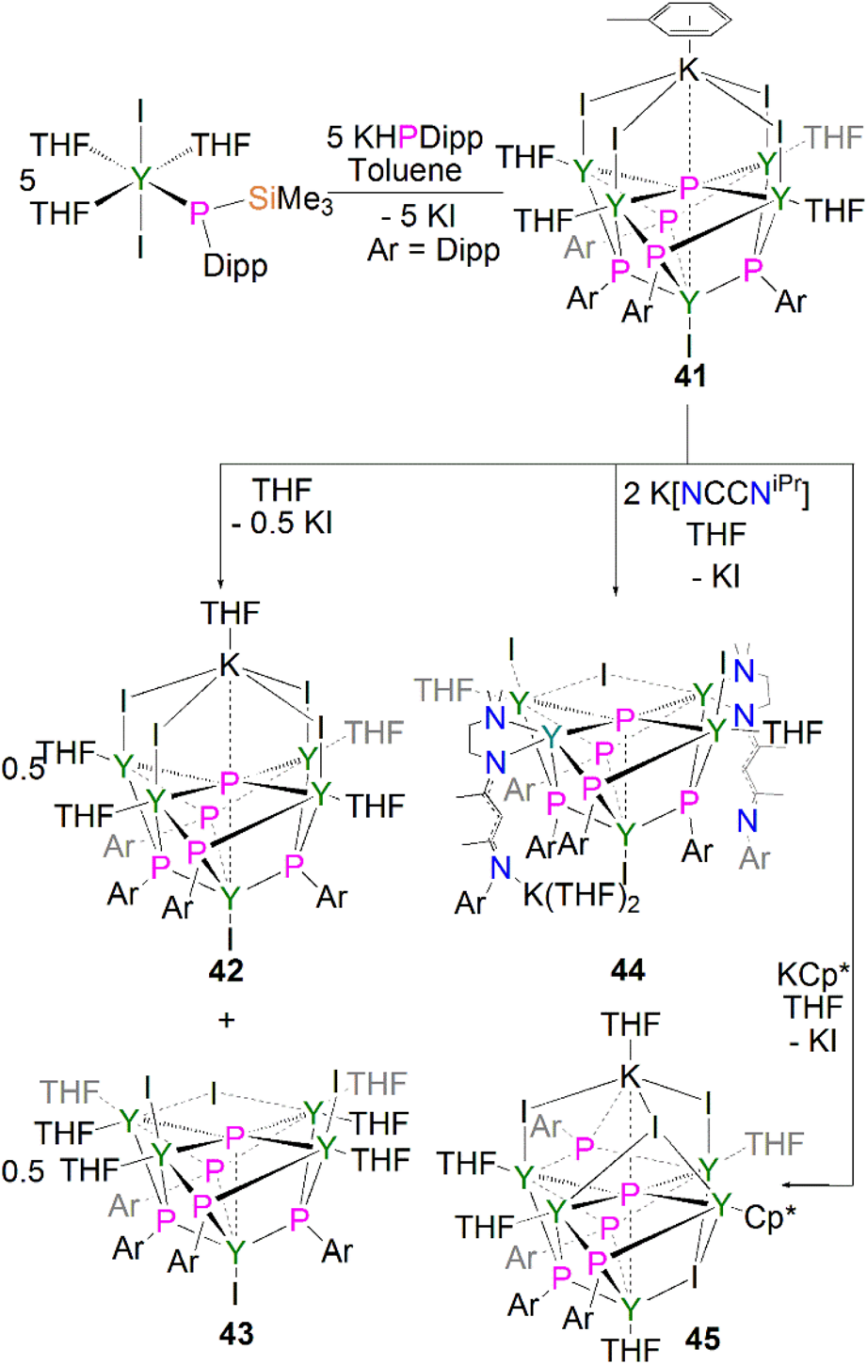
Synthesis of yttrium phosphido complexes 41–45.

In 2011, Chen and co-workers reported the first structurally authenticated rare earth phosphido complex, [{Y(I)}{Y[μ_3_-P(Dipp)](μ-I)(THF)}_4_(μ_6_-P){K(C_7_H_8_)}] (41) *via* a salt elimination/protonolysis protocol, Scheme 14.^62^ The reaction of [Y{P(SiMe_3_)(Dipp)}(I)_2_(THF)_3_] with one equivalent of K[P(H)Dipp] gave 41 in a 33% yield. The product formed depends upon the reaction solvent; dissolution of 41 in THF results in the loss of half an equivalent of potassium iodide and the conversion of 41 to half an equivalent each of the THF adduct, [{Y(I)}{Y[μ_3_-P(Dipp)](μ-I)(THF)}_4_(μ_6_-P){K(THF)}] (42), and the potassium–free complex [{Y(I)}{Y(I)(THF)}_2_{Y(THF)_2_}_2_(μ-I)[μ_3_-P(Dipp)]_4_(μ_5_-P)] (43). The authors attempted to displace the yttrium-bound THF molecules by exposing 41 to potassium salts of NCCN^iPr^ and Cp*, yielding the corresponding phosphido complexes [{Y(I)}{Y(I)(THF)}_2_{Y(NCCN^iPr^)}_2_(μ-I)[μ_3_-P(Dipp)]_4_(μ_5_-P)] (44) and [{Y(THF)}{Y(μ-I)(THF)}_2_{Y(THF)}{Y(Cp*)}(μ_3_-I)(μ-I)[μ_3_-P(Dipp)]_4_(μ_6_-P){K(THF)}] (45), respectively.^62^ The phosphido centres in complexes 41–45 are stabilised through interactions with five yttrium ions where bridging phosphinidiide ligands are present as well. In the cases of 41, 42 and 45 an additional interaction with a potassium ion is present, rendering the phosphido bridging modes as either (μ_5_-P)^3−^ or (μ_6_-P)^3−^. The phosphido ions in 41–45 lie in the plane of the four equatorial yttrium ions with near-linear mean Y_eq_–P–Y_eq_ bond angles [164.89(6)–175.6(2)°], giving the phosphido centres octahedral or square pyramidal geometries (depending upon the coordination of a potassium ion). The four equatorial Y_eq_–P bond distances found in 41–45 [range: 2.6720(11)–2.8787(14) Å] are shorter than the axial Y_ax_–P distances [range: 2.885(2)–3.179(2) Å]. The phosphido cores in 41–45 exhibit ^31^P NMR resonances between 300.9 and 400.7 ppm, which are shifted downfield compared to their phosphinidiide counterparts in these clusters [72.8–176.3 ppm].

### 4.6 Lanthanide polyphosphorus complexes

**Fig. 6:**
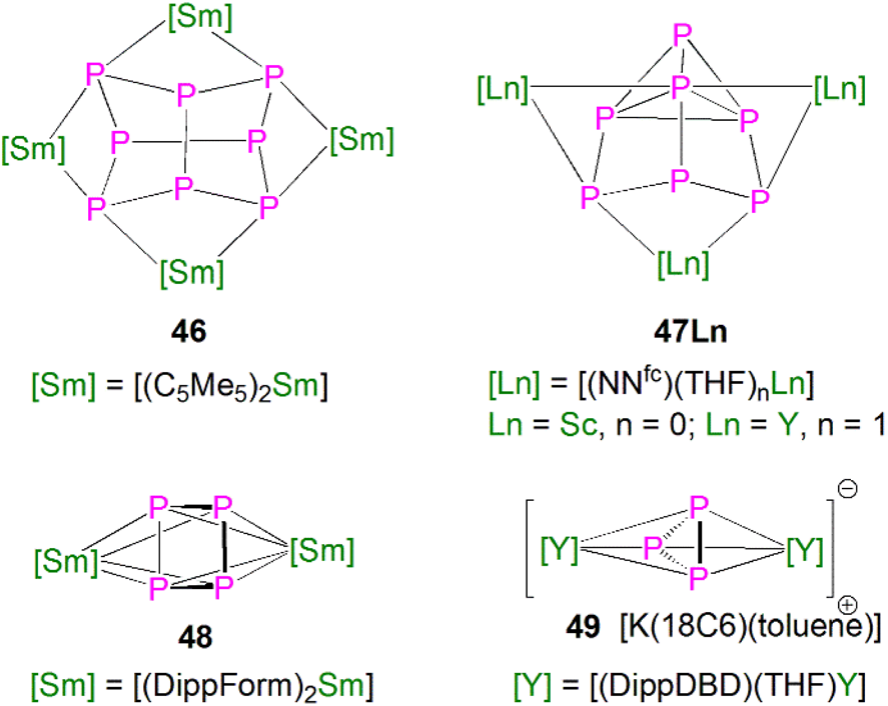
Rare earth metal polyphosphorus complexes 46–49.

This class of lanthanide complexes are normally synthesised by the reactions of reducing low valent lanthanide precursors with P_4_ or transition metal polyphosphorus compounds *via* redox methods.^26^ In 2009, Roesky and co-workers reported the synthesis and molecular structure of the first molecular lanthanide metal polyphosphorus complex [{Sm(Cp*)_2_}_4_P_8_] (46)^63^ prepared by diffusion of P_4_ vapour into a toluene solution of solvent-free samarocene involving a four electron transfer process, Fig. 6. The solid-state structure of 46 revealed a very rare structure that can be seen as a realgar-type P_8_^4−^ ligand, which is isoelectronic with the P_4_S_4_ molecule, trapped in a cage of four [Sm(Cp*)_2_] cations. The Sm–P bond distances of 46 range from 2.997(2) Å to 3.100(2) Å.

A few years later, Diaconescu and co-workers reported another rare earth metal P_8_ cluster [{Sc(NN^fc^)}_4_P_8_] (NN^fc^ = 1,1-fc(NSi^*t*^BuMe_2_)_2_, fc = ferrocenylene) prepared by the reaction of the scandium naphthalendiide complex [{Sc(NN^fc^)}_2_(μ-C_10_H_8_)] with P_4_.^64^ The core P_8_^4−^ ligand in this molecule is very similar to that in 46. Interestingly, using excess P_4_ in the reactions with [{Sc(NN^fc^)}_2_(μ-C_10_H_8_)] and [{Y(NN^fc^)(THF)}_2_(μ-C_10_H_8_)] yielded the P_7_ complexes [{(NNfc)Ln(THF)_*n*_}_3_P_7_] (47Ln, Ln = Sc, *n* = 0; Ln = Y, *n* = 1), respectively, Fig. 6. Complexes 47Ln are the first examples of rare earth metal Zintl P_7_ compounds prepared from P_4_ activation directly. The solid-state structures of 47Sc and 47Y revealed that the P_7_^3−^ units in both cases are similar to the molecular structure of the inorganic salt Li_3_P_7_. The ionic interactions between the rare earth metals and the Zintl P_8_^4−^ or P_7_^3−^ units above was confirmed by DFT calculations.

Although actinide-P_5_ and P_6_ complexes are known,^20^ and indeed *cyclo*-P_5_ complexes are well-known for transition metals, such species remain rare for the lanthanides.^26^ Examples of lanthanide-P_5_/P_6_ complexes have been stabilised by transition metals, resulting in 3d/4d-4f clusters. For instance, the first lanthanide complex containing *cyclo*-P_5_ bridged by lanthanide and transition metals, [{Fe(Cp*)}(μ-P_5_){Sm(DPIP)(THF)_2_}] (DPIP = 2,5-bis{*N*-(2,6-diisopropylphenyl)iminomethyl}pyrrolyl), was prepared from the reaction of [Sm(DPIP)(I)(THF)_3_] with one equivalent of [Fe(Cp*)(μ-P_5_)] in THF in the presence of potassium-naphthalenide.^65^ Interestingly, whilst dinuclear [{Fe(Cp*)}(μ-P_5_){Sm(DPIP)(THF)_2_}] was obtained when the product was recrystallised from THF and toluene, recrystallisation from toluene and pentane gave tetranuclear [{Fe(Cp*)(μ-P_5_)Sm(DPIP)}_2_]. In contrast, reacting samarocenes with [Fe(Cp*)(μ-P_5_)] produced [{Fe(Cp*)}_2_(μ-P_10_){Sm(Cp*)_2_}_2_] (R = Me or ^*n*^Pr) containing a [P_10_]^4−^ unit, which was also the first example of a 3d-P_10_-4f complex.^66^ Well-defined *cyclo*-P_3_ and -P_4_ lanthanide complexes have been reported more recently. For example, in 2018, Roesky and co-workers reported the lanthanide *cyclo*-P_4_ complex [{Sm(DippForm)_2_}_2_(μ^2^-η^4^:η^4^-P_4_)] (48, DippForm = {(NDipp)_2_CH}) by the reduction of P_4_ with the divalent precursor [Sm(DippForm)_2_(THF)_2_], Fig. 6.^67^ In 2019, Zhang, Zhou and co-workers reported the lanthanide *cyclo*-P_3_ complex [K(18C6)(toluene)][{Y(DippDBD)(THF)}_2_(P_3_)] (49, DippDBD = *N*,*N*′-2,6-diisopropylphenyl-1,4-diazabutadiene), with the central P_3_^3−^ trianion bridging two Y(iii) metal ions, prepared by alkyl migration of an organosubstituted *cyclo*-P_4_R_2_ precursor followed by encapsulation of K^+^ cation with 18-crown-6 reagent, Fig. 6.^68^ Very recently, using a redox synthetic strategy, Roesky and co-workers reported another two *cyclo*-P_3_ and -P_4_ inverse sandwich complexes for lanthanides supported by a xanthene-diamide ligand.^69^ Lastly, diphosphorus (P_2_) complexes, which are heavy N_2_ analogues, remain elusive for lanthanides because of the synthetic challenges of making and stabilising P_2_.^69^

## 5. Actinide phosphorus complexes

Although actinide–nitrogen chemistry is well-developed over several decades, this is not the case for actinide–phosphorus chemistry.^16,20^ Nevertheless, actinide–phosphorus chemistry is the most developed compared with the analogous lanthanide chemistry, likely because actinides can deploy 5f and 6d orbitals in bonding to form more covalent chemical bonds than lanthanides. In this section, seminal examples in actinide phosphorus chemistry are highlighted, noting that after initial work on phosphine derivatives that remains contemporaneous,^70,71^ binding phosphines to amides has supported metal–metal bonds.^72–75^

### 5.1 Actinide phosphide complexes

In terms of actinide complexes containing metal–phosphorus single bond interactions, monoanionic charged phosphide ligands can have stronger interactions with actinide metal centres than neutral phosphine ligands, which only form dative bonds to actinide ions.^20^ Furthermore, some actinide phosphide complexes are useful precursors to novel linkages such as actinide–metal and -ligand multiple bonds (see below), low-valent U–P bonds,^76^ and hydrophosphination catalysts.^77,78^

**Scheme 15:**
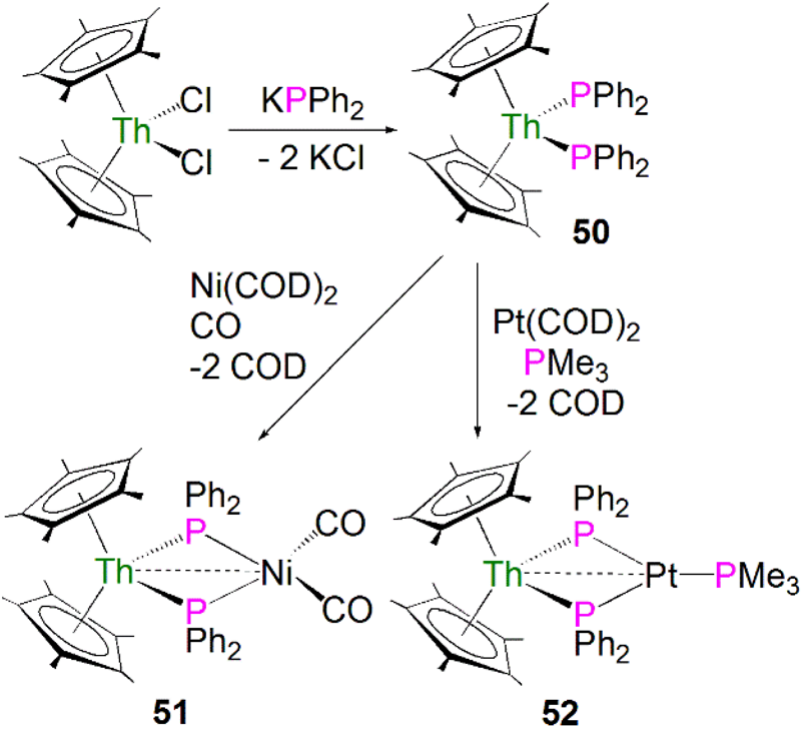
Synthesis of Th phosphide complexes 50–52.

The first examples of actinide phosphide complexes were reported in 1985 by Ryan and co-workers. The mononuclear complex [Th(Cp*)_2_(PPh_2_)_2_] (50) was prepared by a salt metathesis reaction between [Th(Cp*)_2_(Cl)_2_] and potassium diphenylphosphide, Scheme 15.^79^ Importantly, the phosphide ligands are able to coordinate to transition metals as well, supporting actinide and transition metal interactions, making it possible to investigate actinide–metal bonding. Treatment of 50 with [Ni(COD)_2_] (COD = 1,5-cyclooctadiene) under a CO atmosphere, or [Pt(COD)_2_] in the presence of PMe_3_, led to the formation of the heterobimetallic compounds [Th(Cp*)_2_(μ-PPh_2_)_2_Ni(CO)_2_] (51)^79^ and [Th(Cp*)_2_(μ-PPh_2_)_2_Pt(PMe_3_)] (52)^80^ in moderate yields, respectively, Scheme 15. The ^31^P{^1^H} NMR spectrum for 51 exhibits a signal at 177 ppm, which is shifted downfield from the resonance for 50 found at 143 ppm. In contrast, the ^31^P NMR spectrum for 52 shows a doublet at 149.3 ppm, and a triplet at −3.3 ppm. These were attributed to the [PPh_2_]^−^ and PMe_3_ ligands, respectively. Both resonances contain coupling to Pt, suggesting a direct interaction between the phosphorus atoms of both ligands and the transition metal. The solid-state structure of 51 revealed the Th–P bond lengths to be 2.869(4) and 2.900(4) Å, which are close to the Th–P distance of 2.866(7) Å found in the mononuclear starting material 50. The Th–P bond lengths in 52 are unexceptional and are similar to those in 50. The distance between the thorium and nickel atoms in 51 was found to be 3.206(2) Å, which is longer than the sum of the covalent single bond radii of Th and Ni (2.85 Å). In contrast, in 52 the distance between the thorium and platinum centres was found to be 2.984(1) Å, which is similar to the sum of the covalent single bond radii of Th and Pt (2.98 Å). The metal–metal bonding interactions in 51 and 52 were interpreted as a weak, donor–acceptor dative bonds from the low-valent electron-rich Ni(0) and Pt(0) ions to the electron-poor thorium(iv) ions.

**Fig. 7:**
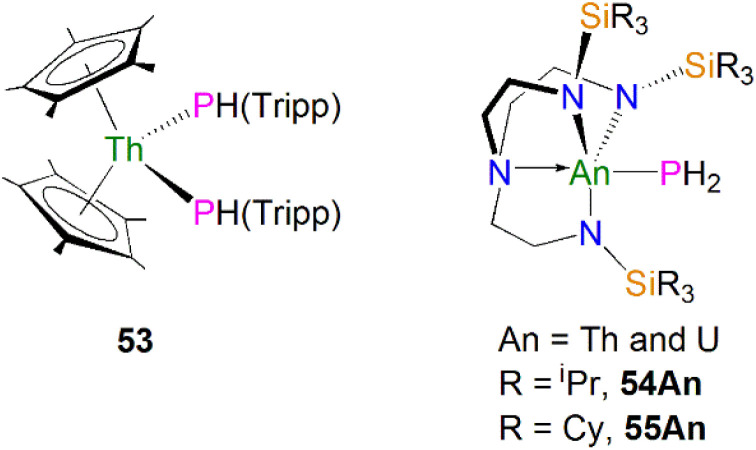
Actinide phosphide and phosphanide complexes 53-55An.

As well as actinide phosphide complexes with secondary phosphide ligands, there are also some actinide complexes containing primary phosphide ligands, which could potentially be used to access actinide phosphorus multiple bonds. For example, in 2015 Walensky and co-workers reported the thorium bisphosphide complex [Th(Cp*)_2_(PHTripp)_2_] (53, Tripp = 2,4,6-^i^Pr_3_C_6_H_2_), prepared by the reaction of H_2_PTripp with the thorium dimethyl precursor [Th(Cp*)_2_(CH_3_)_2_] in a 2 : 1 stoichiometric ratio *via* an alkane elimination method, Fig. 7.^81^ The Th–P bond distances in 53 (2.8754(6) and 2.8830(6) Å) are very close to those in 50–52, indicative of typical single bond interactions. However, the ^31^P{^1^H} NMR spectrum of 53 has a resonance at 1.66 ppm, whereas the ^31^P{^1^H} resonances in 50–52 range from 143 to 177 ppm. Complex 53 is a useful precursor to thorium phosphinidiide and phosphinidene complexes (see below). Unlike complexes 50–53, which contain bulky phosphide substituents, by using bulky triamidoamine ligand frameworks Liddle, Scheer, and co-workers synthesised the first actinide parent phosphanide complexes [An(Tren^R^)(PH_2_)] (Tren^R^ = {N(CH_2_CH_2_NR)_3_}^3−^, R = TIPS, triisopropylsilyl, An = Th and U, 54An; R = TCHS, tricyclohexylsilyl, An = Th and U, 55An), Fig. 7.^82–84^ Complexes 54An were prepared by salt metathesis reactions of the corresponding actinide precursors with NaPH_2_,^82,84^ whilst 55An were synthesised by protonation of their respective terminal parent phosphinidene complexes due to the lack of suitable actinide precursors for salt metathesis reactions.^84^ These four terminal phosphanide complexes are the only known examples to date for any f-element. The solid-state structures of 54An and 55An revealed trigonal-bipyramidal metal geometries with the parent phosphide group well-protected by the bulky silyl substituents. The An–P bond distances are 2.982(2), 2.883(2), 3.0360(15), and 2.8725(13) Å for 54Th, 54U, 55Th, and 55U, respectively, which are slightly longer than the respective sum of the single bond covalent radii for thorium and phosphorus (2.86 Å) and uranium and phosphorus (2.81 Å). Due to the P–H coupling, the ^31^P NMR spectra for 54Th, 54U, 55Th, and 55U exhibit triplet resonates at −144.1, 595.0, −133.0 and 605.9 ppm, respectively. As expected, the resonances for uranium complexes are significantly shifted owing to the paramagnetic nature of 5f^2^ uranium(iv). These phosphanide complexes have proven to be key precursors to access actinide–phosphorus multiple bonds (see below).

### 5.2 Actinide phosphinidiide complexes

Phosphinidiide complexes can be viewed as polynuclear forms of phosphinidene complexes where the phosphinidene ligands are bridged between two or more electropositive metal ions to enhance the stability of the reactive moiety. As discussed above, phosphinidiide complexes are also more common than terminal phosphinidenes for both lanthanides and actinides, but some phosphinidiide complexes can still have metal–phosphorus multiple bonding character.

**Scheme 16:**
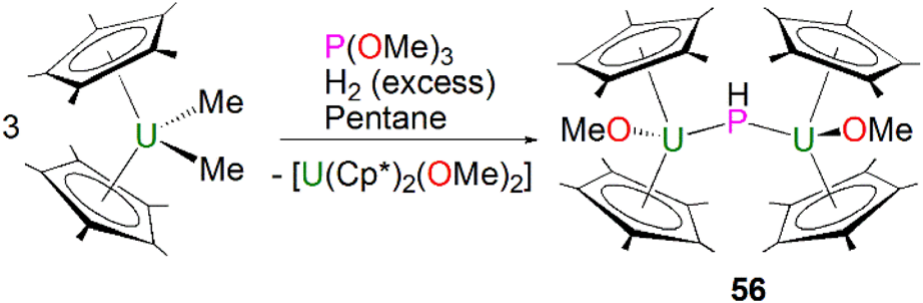
Synthesis of the U–phosphinidiide complex 56.

The first crystallographically characterised actinide phosphinidiide complex was isolated in 1984 by Marks, Day and co-workers, as a parent (HP)^2−^ unit bridging two uranium(iv) centres. Mixing three equivalents of [U(Cp*)_2_(Me)_2_] with one equivalent of P(OCH_3_)_3_ and excess hydrogen gave [{U(Cp*)_2_(OMe)}_2_(μ-PH)] (56) in yields of 42%, Scheme 16.^85^ The authors additionally prepared [{Th(Cp*)_2_(OMe)}_2_(μ-PH)] in an analogous manner. Marks and Day postulated that the mechanism for the synthesis of 56 proceeded *via* an actinide hydride. To confirm this, additional reactions were conducted in the absence of hydrogen or utilising [An(Cp*)_2_(H)_2_]_2_ as starting materials. The former showed no detectable reaction, whilst the latter gave 56. Complex 56 exhibits two U–P distances of 2.743(1) Å and a U–P–U angle of 157.7(2)°. The IR spectrum of 56 confirmed the presence of the parent phosphinidiide, with a PH stretching mode at 2193 cm^−1^ (*υ*_P–H_/*υ*_P–D_ = 1.39). Liddle, Scheer, and co-workers also reported diactinide parent phosphinidiide complexes supported by triamidoamine ligands,^86^ which will be discussed in the phosphido section below for comparison purposes.

**Scheme 17:**
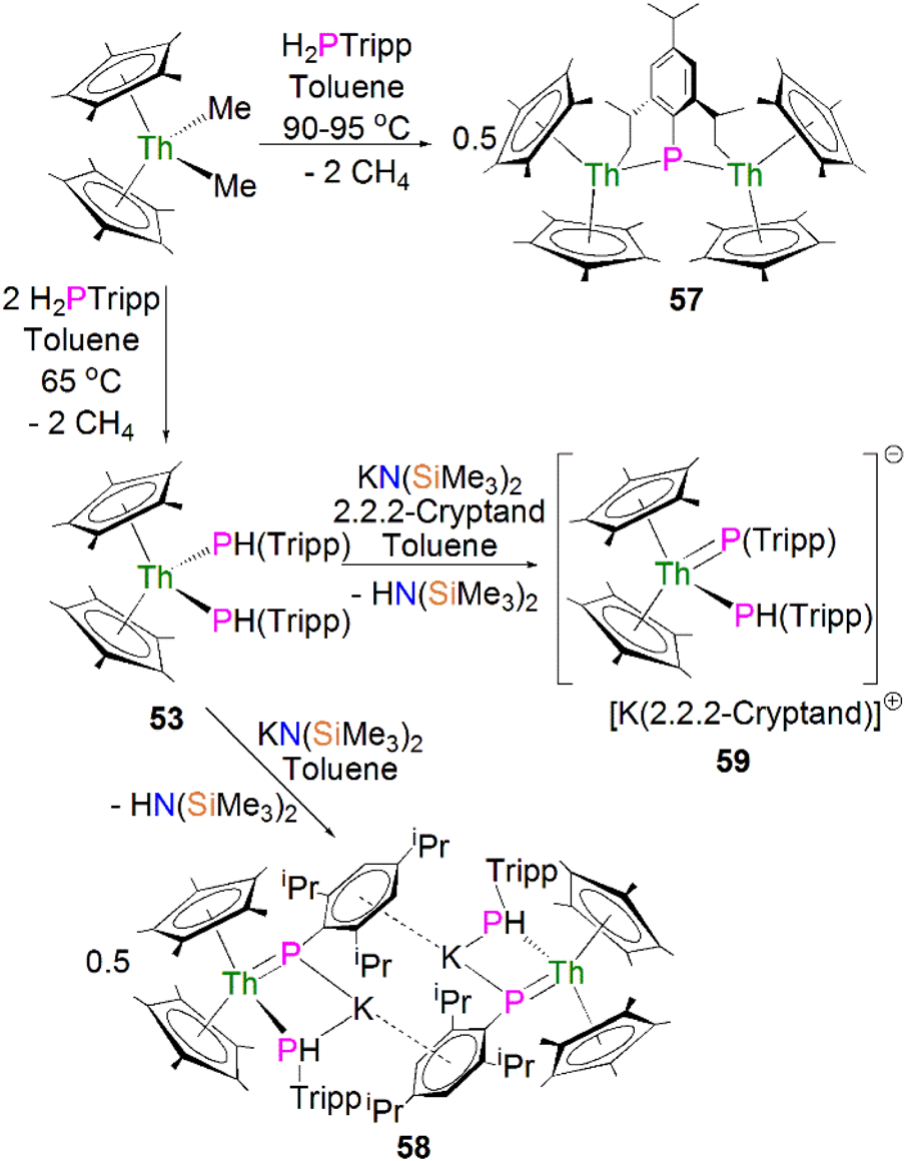
Synthesis of Th–phosphinidiide complexes 57 and 58 and phosphinidene 59.

In 2015 Walensky and co-workers utilised the bis-Cp* framework to synthesise the bridging thorium phosphinidiide complex [{Th(Cp*)_2_}_2_{μ-P[(2,6-CH_2_CHCH_3_)_2_-4-^i^PrC_6_H_2_]}] (57, 63%) by the protonolysis of two equivalents of [Th(Cp*)_2_(Me)_2_] with one equivalent of H_2_PTripp at 90–95 °C, Scheme 17.^81^ Complex 57 exhibits Th–P bond distances of 2.8083(9) and 2.8186(9) Å, which are marginally shorter than those observed for the thorium phosphide complex 53 (2.8755(6) and 2.8829(7) Å) due to the bridging mode of the phosphinidiide.

Walensky and co-workers also derivatised 53 to synthesise the bridging phosphide/phosphinidiide thorium complex [{Th(Cp*)_2_(μ-PTripp)(μ-PHTripp)(K)}_2_] (58), Scheme 17,^87^ which paved the way to a number of derivatives.^88–92^ The reaction of 53 with one equivalent of KN(SiMe_3_)_2_ gave 58 in a yield of 64% *via* the elimination of one equivalent of HN(SiMe_3_)_3_. It was found that if the deprotonation reaction was conducted in the presence of 2.2.2-cryptand the monomeric phosphide/phosphinidene complex [K(2.2.2-cryptand)][Th(Cp*)_2_(PTripp)}(PHTripp)] (59) was obtained.^87^ Complex 58 exhibits Th–P distances of 2.6957(10) Å, whilst 59 demonstrates a shorter Th–P bond length of 2.6024(9) Å, which is a result of the encapsulation of the potassium ion allowing more electron density to be donated from the phosphorous atom to the thorium centre.

**Scheme 18:**
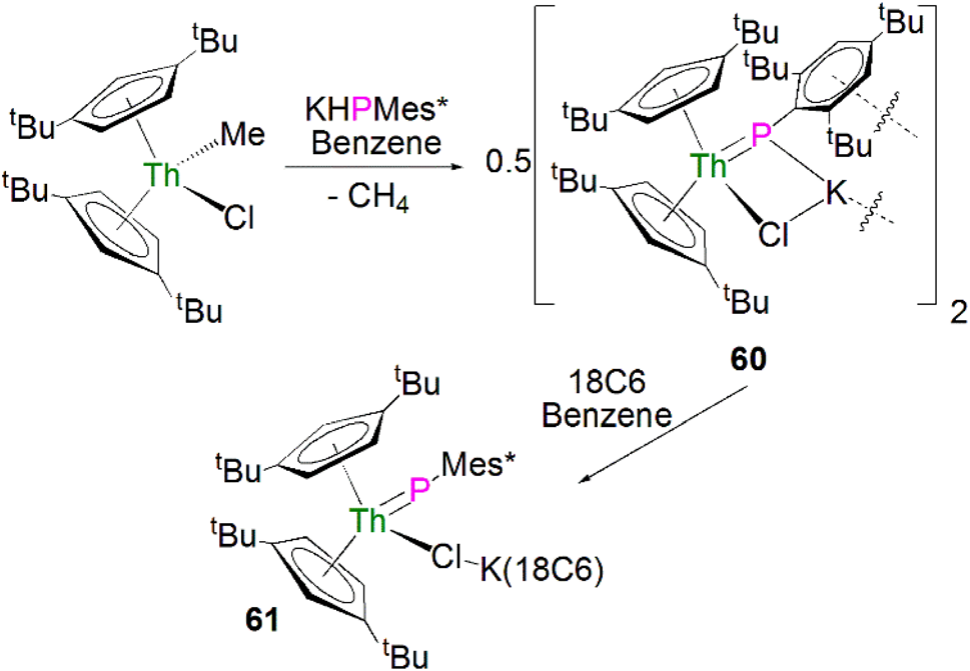
Synthesis of thorium phosphinidiide and phosphinidene complexes 60 and 61.

Recently, Walter, Ding, Zi and co-workers reported the salt metathesis/protonolysis reaction of one equivalent each of [Th(Cp^tt^)_2_(Me)(Cl)] (Cp^tt^ = 1,3-^*t*^Bu_2_-C_5_H_3_) and KPH(Mes*) to synthesise the dinuclear phosphinidiide complex [{Th(Cp^tt^)_2_(PMes*}(ClK)}_2_] (60) in 78% yield, Scheme 18, with elimination of one equivalent each of methane and potassium chloride.^93^ The authors reported that 60 could be converted to the mononuclear terminal phosphinidene complex [Th(Cp^tt^)_2_(PMes*)(μ-Cl){K(18C6)}] (61, 92%) by the addition of 18-crown-6.^93^ The solid-state structure of 60 and 61 revealed that 60 exhibits a slightly shorter Th–P distance of 2.560(1) Å and a more acute Th–P–C^Ar^ angle of 162.6(2)° than found in 61 (2.582(1) Å; Th–P–C^Ar^ angles = 171.3(1)°) which is most likely a result of the removal of the interaction between phosphorus and the potassium cation from 60 to 61. The authors reported that all four complexes 58–61 exhibit ^31^P{^1^H} NMR resonances in the range 108.8 to 177.85 ppm.

### 5.3 Actinide phosphinidene complexes

Although uranium imido chemistry is well-developed,^18^ actinide complexes containing heaver pnictogen An = PnR (An = actinide; Pn = P, As, Sb, Bi) multiple bonds are scarce.^20^ To date, there are few complexes containing An=PnR double bonds outside of cryogenic matrix isolation conditions, but only with P or As ligands, not for Sb and Bi ligands. Also, there is no example of a terminal actinide heavy pnictido An≡Pn triple bond isolated under ambient conditions. Even for d-block metals there are relatively few structurally characterised terminal metal pnictinidene complexes (∼50), far fewer than their imido counterparts (>3000).^94^ Actinide pnictinidene complexes normally require kinetic stabilisation by sterically demanding ancillary ligands, coupled with bulky pnictinidene substituents (*cf.*59 and 61).

**Scheme 19:**
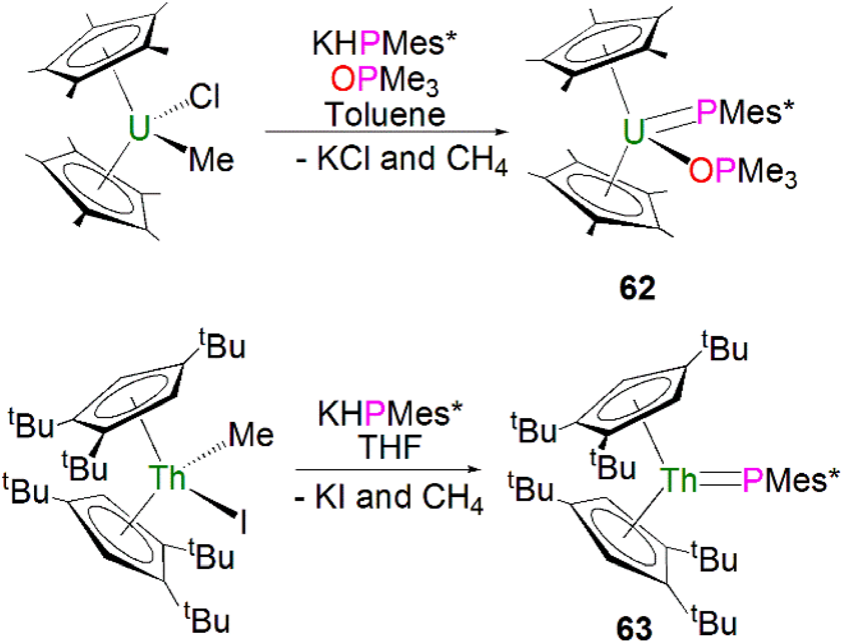
Synthesis of terminal uranium and thorium phosphinidene complexes 62 (top) and 63 (bottom).

In 1996, Burns and co-workers reported the first example of a terminal uranium phosphinidene complex utilising the sterically demanding Cp* supporting ligand. The salt elimination/protonolysis reaction of one equivalent each of [U(Cp*)_2_(Me)(Cl)] and KHP(Mes*) in the presence of OPMe_3_ gave [U(Cp*)_2_(PMes*)(OPMe_3_)] (62), in yields of up to 62%, Scheme 19 (top).^95^ Complex 62 exhibits a U–P distance of 2.562(3) Å, which is shorter than the bridging phosphinidiide U–P distance in 56 or, for example, the U–P distance in the phosphide complex [U(Cp*)_2_{P(SiMe_3_)_2_}(Cl)] (2.789(4) Å).^96^ The authors postulated that the U–P–C^Ar^ angle of 143.7(3)° in 62 was due to crystal packing forces, or a result of the combination of heavy main group elements generally adopting bent geometries in addition to the preferred linear geometry required to minimise P–U π-overlap.

In 2018, using sterically demanding cyclopentadienyl ligands, Walter, Ding, Zi and co-workers reported a base-free terminal thorium phosphinidene [Th(Cp^ttt^)_2_(PMes*)] (63, Cp^ttt^ = 1,2,4-^*t*^Bu_3_-C_5_H_2_), Scheme 19 (bottom).^97^ Complex 63 was prepared by a similar salt metathesis/protonolysis reaction that was employed to prepare 62; reaction of one equivalent each of [Th(Cp^ttt^)_2_(Me)(I)] and KPH(Mes*) produced 63 in high yield (80%), with elimination of one equivalent each of methane and potassium iodide. The molecular structure of 63 further confirmed a base-free terminal phosphinidene species with a short Th–P bond distance (2.536(2) Å). The ^31^P{^1^H} NMR spectrum of 63 shows one resonance at 145.7 ppm, which is close to those for 59 and 61. Calculations probing the Th=P bonding of 63 and the theoretical monomeric model of 63 suggest more covalency in this linkage than related imido complexes. The authors additionally treated the thorium complexes 63 with a wide range of unsaturated substrates to probe the reactivity of Th–P double bond. In general, the actinide metallocene phosphinidene motif has proven practicable to effect with a range of cyclopentadienyl substituents.^98–105^

From the above examples, it is clear that sterically demanding cyclopentadienyl ligands have proven effective at stabilising actinide phosphinidiide and phosphinidene complexes, but sterically demanding triamidoamine ligands have in parallel extended this chemistry into a new regime. Following the successful isolation of terminal uranium nitrides supported by the Tren^TIPS^ ligand (Tren^TIPS^ = {N(CH_2_CH_2_NSi^i^Pr_3_)_3_}^3−^) in 2012 and 2013,^106,107^ Liddle, Scheer, and co-workers utilised this bulky ligand framework to stabilise the first terminal uranium parent phosphinidene in 2014, Scheme 20. The reaction of 54U with one equivalent of benzyl potassium and two equivalents of benzo-15-crown-5 ether (B15C5) yielded the terminal parent uranium(iv) phosphinidene complex [K(B15C5)_2_][U(Tren^TIPS^)(PH)] (64K).^82^ The synthesis of the sodium analogue [Na(12C4)_2_][U(Tren^TIPS^)(PH)] (64Na) was achieved by reaction of the uranium cyclometallate complex [U{N(CH_2_CH_2_NSi^i^Pr_3_)_2_(CH_2_CH_2_NSi^i^Pr_2_C(H)MeCH_2_)}] (65U) with one equivalent of NaPH_2_ and two equivalents of 12-crown-4 ether (12C4).^86^ The authors reported that the treatment of 54U with one equivalent each of KCH_2_Ph and 2.2.2-cryptand gave the contact ion pair phosphinidiide complex [{U(Tren^TIPS^)(μ-PH)}{K(2.2.2-cryptand)}] (66).^82^ The solid-state structures revealed that the terminal phosphinidene complexes 64M and phosphinidiide 66 exhibit U–P distances ranging from 2.613(2) to 2.685(2) Å, which are shorter than that in the parent phosphide precursor 54U (2.883(2) Å). The authors reported that the IR spectrum of 64K exhibits a P–H stretch of 2360 cm^−1^. The U–P bond lengths in 64M are longer than those observed for 62 (2.562(3) Å) lying between the sum of the covalent single and double bond radii for uranium and phosphorus (2.81 Å and 2.36 Å, respectively); this reflects the sterically demanding nature of Tren^TIPS^ and indicates polarised covalent U=P interactions that is confirmed by DFT calculations.

**Scheme 20:**
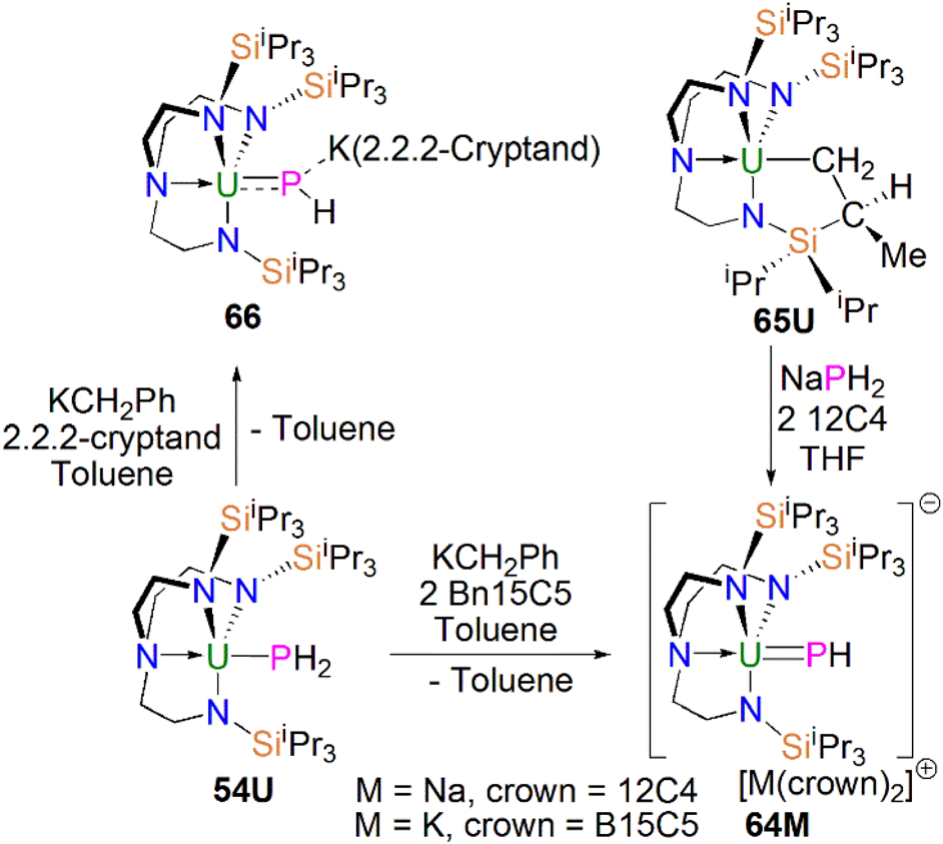
Synthesis of uranium phosphinidene and phosphinidiide complexes 64M and 66.

**Scheme 21:**
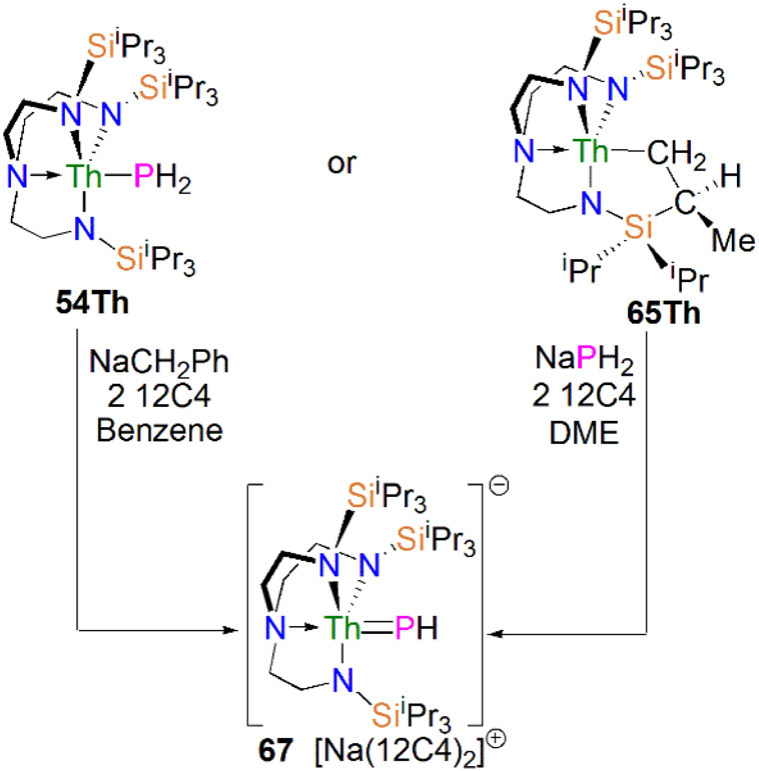
Synthesis of thorium phosphinidene complex 67.

In 2016, Liddle, Scheer, and co-workers utilised the Tren^TIPS^ ligand framework to stabilise terminal thorium parent phosphinidene analogues. The two methodologies used to synthesise the uranium complexes 64M were adapted to prepare the terminal phosphinidene thorium complex [Na(12C4)_2_][Th(Tren^TIPS^)(PH)] (67), Scheme 21.^83^ Complex 67 could be synthesised either through deprotonation of 54Th with one equivalent of NaCH_2_Ph and two equivalents of 12C4, or by the reaction of the thorium cyclometallate complex [Th{N(CH_2_CH_2_NSi^i^Pr_3_)_2_(CH_2_CH_2_NSi^i^Pr_2_C(H)MeCH_2_)}] (65Th) with one equivalent of NaPH_2_ and two equivalents of 12C4 in yields of up to 38%. Complex 67 is isostructural with the uranium phosphinidene complex 64Na.

The Th–P bond length of 2.758(2) Å in 67 is *ca.* 0.22 Å shorter than that of the Th–PH_2_ bond in 54Th (2.982(2) Å), but is longer than the U=P double distance of 2.613(2) Å in 64K, suggesting a more polarised double bond interaction for Th=PH linkage. This is in accord with a smaller Th–P Mayer bond order of 1.67 in 67 than that of 1.92 in 64K. The Th=P–H angle of 67.45(8)° in 67 indicates an ‘agostic-type’ interaction between the metal ion and the electron density of the P–H bond, whereas this interaction was not observed in 64K, which has a U=P–H angle of 118.8(9)°. The ^31^P NMR spectrum for 67 has a doublet resonance at 198.8 ppm due to P–H coupling, further confirming the presence of [PH]^2−^ group at thorium. By contrast, because of the strong paramagnetic shielding from the uranium(iv) centre, no resonance was observed in the ^31^P NMR spectra for 64M.

**Scheme 22:**
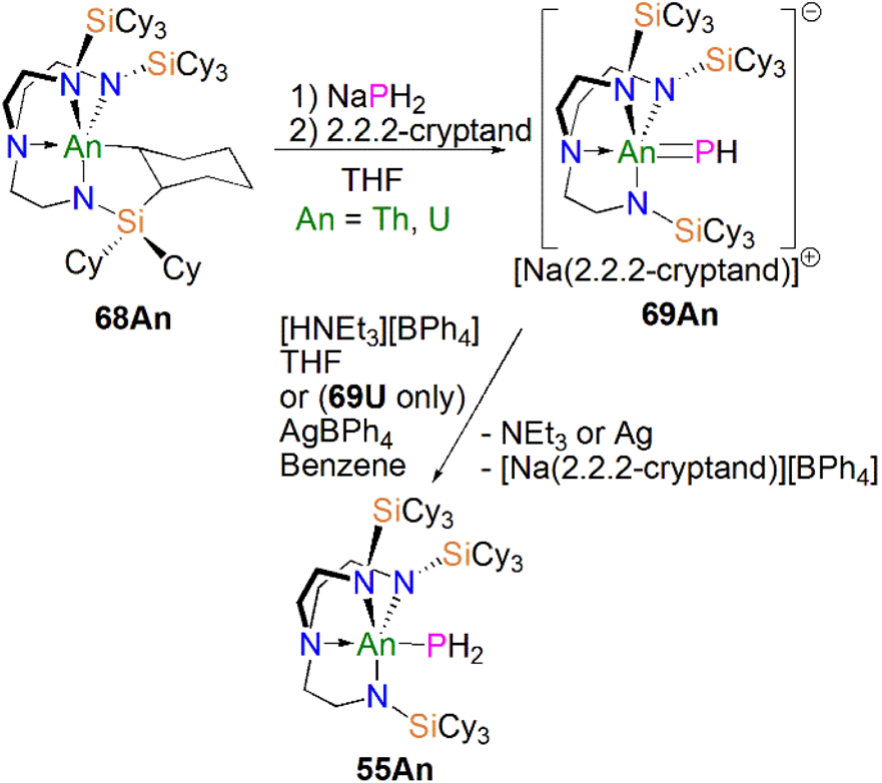
Synthesis of thorium and uranium phosphinidene complexes 69An and phosphanide complexes 55An.

Protonation of reactive actinide–carbon bonds has proven to be an effective strategy for constructing actinide–pnictogen multiple bonds. In 2022, Liddle, Scheer, and co-workers developed a bulky Tren^TCHS^ ligand ({N(NCH_2_CH_2_NSiCy_3_)_3_}^3−^) to prepare two new terminal actinide phosphinidene complexes, Scheme 22. Reaction of the cyclometallate actinide complexes [An{N(CH_2_CH_2_NSiCy_3_)_2_(CH_2_CH_2_NSiCy_2_[CHCH_2_CH_2_CH_2_CH_2_CH])}] (68An, An = Th, U) with NaPH_2_ in the presence of 2.2.2-cryptand in THF afforded [Na(2.2.2-cryptand)][An(Tren^TCHS^)(PH)] (69An, An = U, Th).^84^ The molecular structures of 69An confirmed the presence of terminal phosphinidenes, with the (HP)^2−^ ligand well-protected by the super bulky tricyclohexylsilyl groups. The An–P bond distances of 2.7237(9) and 2.6381(12) Å for 69Th and 69U, respectively are statistically invariant to those in 67 and 64M, indicating multiple bonding interactions in these An=PH linkages with polarised covalent interactions supported by DFT studies. The ‘agostic-type’ interaction between the An ion and the phosphinidene ligand was observed in both structures, with An–P–H angles of 65.83(17)° and 65.23(13)°, respectively. In addition, absorptions corresponding to P–H stretches at 2072 and 2070 cm^−1^ for 69Th and 69U, respectively, were observed in their ATR-IR spectra. The An=P vibrations are also observed in the Raman spectra of 69Th and 69U at 306 and 296 cm^−1^, respectively. The ^31^P NMR spectrum for 69U exhibits a broad resonance at 2629 ppm due to the paramagnetic uranium(iv) centre, while this is not observed in 64M. Similar to 67, 69Th exhibits a doublet resonance at 266.2 ppm in its ^31^P NMR spectrum due to P–H coupling.

Reflecting the basic and nucleophilic nature of 69An, Liddle, Scheer, and co-authors additionally found that treatment of 69An with [HNEt_3_][BPh_4_], as a proton source, in THF resulted in the isolation of the phosphanide complexes 55An in good yields, Scheme 22,^84^ which have similar bond metrics to 54An.^82^ Alternatively, 55U could be prepared by oxidation of 69U with AgBPh_4_ in benzene. The formation of 55U in these oxidation reactions may involve a transient U(v)=PH species (or valence isomer, *e.g.* U(iv)=P˙H), which then abstracts a proton (or H˙) in the reaction mixture due to the HSAB mismatch of U and P. This reactivity contrasts to the disproportionation observed for Tren^TIPS^-supported U(v)=NH chemistry, which produces U(iv)–NH_2_ amide and U(vi)≡N nitride products.^108^ These reactivity outcomes reflect the periodic differences between nitrogen and phosphorus, and that when the latter is paired with electropositive metals P-based electrons can become involved in redox reactions as found in the reactivity of 16.

### 5.4 Actinide phosphido complexes

Actinide phosphido complexes remain exceeding rare. There are only a few bridging dinuclear complexes isolated in recent years with no examples of terminal actinide heavy pnictido An≡Pn triple bond isolated under ambient conditions to date.^20^

**Scheme 23:**
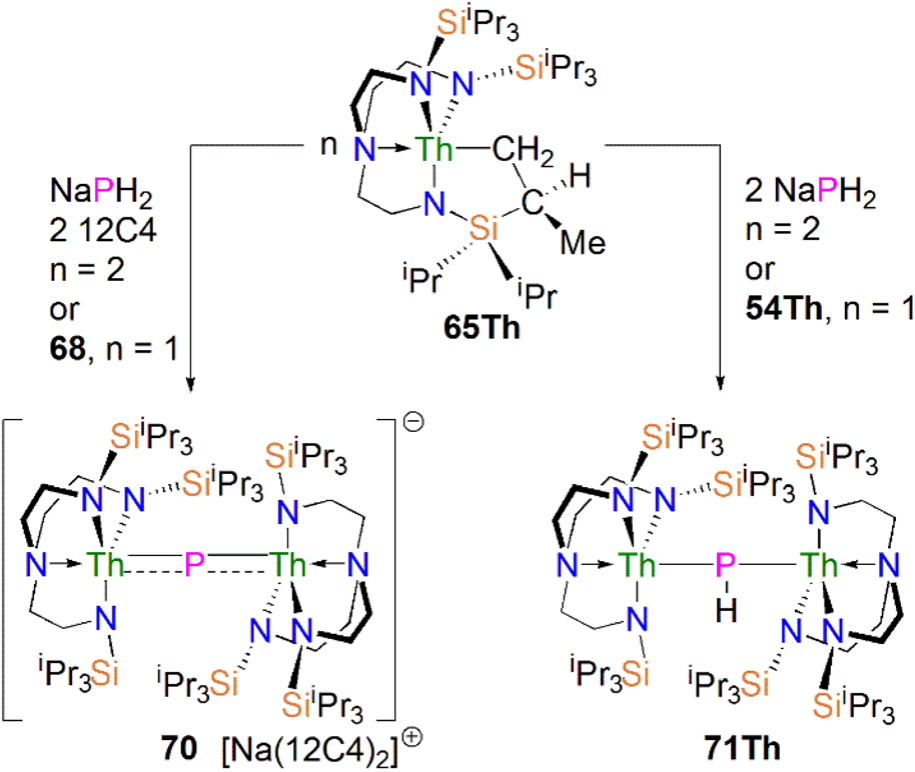
Synthesis of bridging thorium phosphinidiide and phosphido complex 70 and 71Th.

In 2016, Liddle, Scheer, and co-workers reported the first example of an actinide–phosphido complex [Na(12C4)_2_][{Th(Tren^TIPS^)}_2_(μ-P)] (70), where the phosphido ligand bridges two thorium centres, Scheme 23.^83^ Complex 70 was also the first such f-element phosphido species, and was prepared by the reaction of two equivalents of 65Th with NaPH_2_ in the presence of two equivalents of 12C4 in up to 57% yield. Alternatively, 70 can be synthesised by the stoichiometric reaction of 67 with 65Th. The authors additionally reported the synthesis of the bridging phosphinidiide complex [{Th(Tren^TIPS^)}_2_(μ-PH)] (71Th) in a 40% yield, either by treatment of 54Th with one equivalent of 65Th, or the reaction of two equivalents each of 65Th with NaPH_2_, eliminating one equivalent of ‘Na_2_PH’. However, attempts to prepare 70 by deprotonation of 71Th were unsuccessful. Complex 70 has a symmetrical ThPTh core with Th–P bond distances of 2.740(2) and 2.735(2) Å, which are shorter than those in 71Th (2.898(2) Å) but compares well to that of the terminal phosphinidene 67 (2.7584(18) Å), and lies between the sum of the covalent single and double bond radii for thorium and phosphorus (2.86 and 2.45 Å, respectively), suggesting multiple bonding interactions in the ThPTh linkage. The ^31^P NMR spectra for 71Th and 70 exhibit doublet and singlet resonances at 145.7, and 553.5 ppm, confirming the presence of (HP)^2−^ and P^3−^, respectively.

**Scheme 24:**
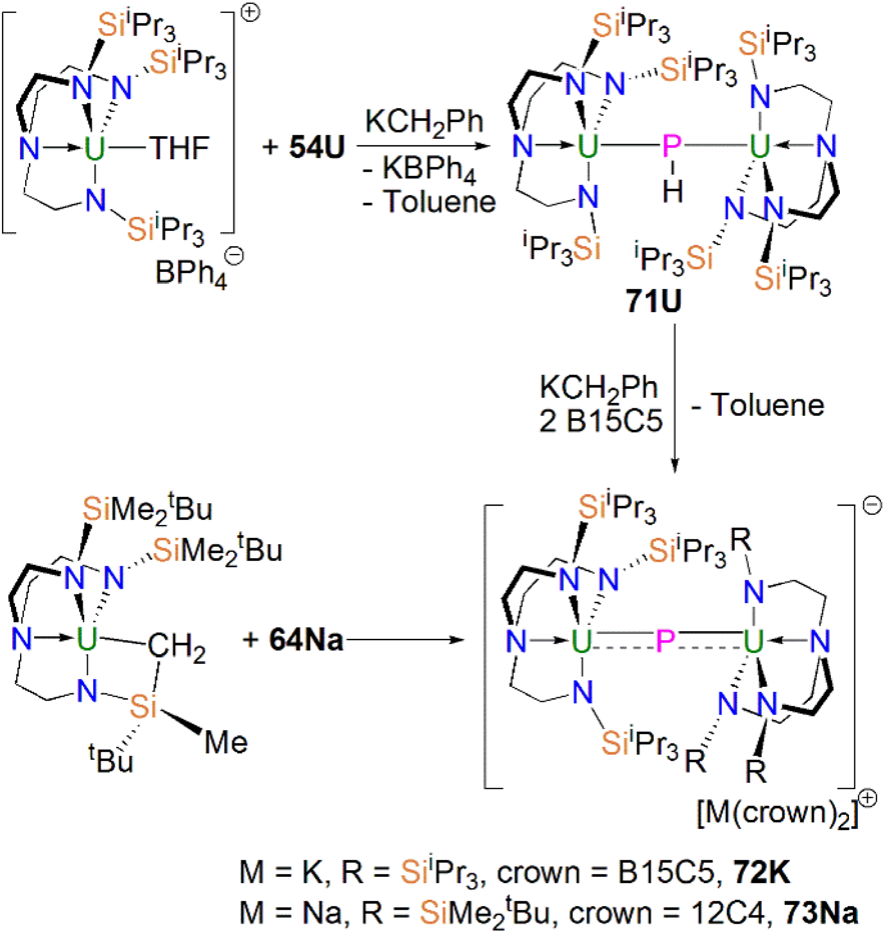
Synthesis of bridging uranium phosphinidiide and phosphido complexes 71U-73Na.

Subsequently, in 2017 Liddle, Scheer, and co-authors reported that addition of one equivalent of KCH_2_Ph to a mixture of 54U and [U(Tren^TIPS^)(THF)][BPh_4_] gave the bridging diuranium phosphinidiide complex [{U(Tren^TIPS^)}_2_(μ-PH)] (71U)^86^ that is isostructural to 71Th, Scheme 24. Importantly, the authors found that deprotonation of 71U with benzyl potassium in the presence of two equivalents of B15C5 produced the diuranium(iv/iv) phosphido compound [K(B15C5)_2_][{U(Tren^TIPS^)}_2_(μ-P)] (72K), Scheme 24;^86^ recall that this method did not work for 71Th.^84^ Complex 72K was found to rapidly decompose, which was attributed to steric overload, and so, seeking a more stable combination, the slightly less bulky and asymmetric sodium analogue of 72K, [Na(12C4)_2_][{U(Tren^TIPS^)}{U(Tren^DMBS^)}(μ-P)] (73Na; Tren^DMBS^ = {N(CH_2_CH_2_NSiMe_2_^*t*^Bu)_3_}^3−^) was prepared^86^ by the reaction of one equivalent each of 64Na with the cyclometallate complex [U{N(CH_2_CH_2_NSiMe_2_^*t*^Bu)_2_(CH_2_CH_2_NSi(Me)(CH_2_)(^*t*^Bu))}], which is a less sterically demanding Tren ligand compared to Tren^TIPS^.

Unlike 70 which is stable, 72K and 73Na are to various extents unstable in solution, in line with the paucity of such phosphido species, which resulted in both complexes being isolated in relatively low yields of 29% and <5% for 73Na and 72K, respectively. Interestingly, 73Na exhibits an asymmetric UPU core, with two U–P bond distances of 2.657(2) and 2.713(2) Å, and the shorter U–P distance is the Tren^TIPS^ ligated unit. In contrast, 72K has a symmetric core with statistically indistinguishable U–P bond lengths of 2.653(4) and 2.665(4) Å, which are shorter than those in the phosphinidiide 71U (2.8187(12) and 2.8110(12) Å). Calculations on 72K and 73Na suggested that the U–P bond are less covalent than the terminal phosphinidene species 64M. This is reflected by smaller U–P Mayer bond orders of 1.41/1.43 and 1.44/1.66 for 72K and 73Na, respectively, when compared to [64K]^−^ (1.92). DFT calculations also suggest that the Th–P bonds in 70 are more polarised and ionic than the U–P bonds in 72K and 73Na, as evidenced by smaller Th–P Mayer bond orders of 1.26 and 1.28 for [70]^−^. However, these An–P Mayer bond orders are nearly twice that of the single U–N_amide_ bonds (∼0.71), suggesting that these An–P bonds are polarised multiple bond interactions.

### 5.5 Actinide 2-phosphaethynolate complexes

**Scheme 25:**
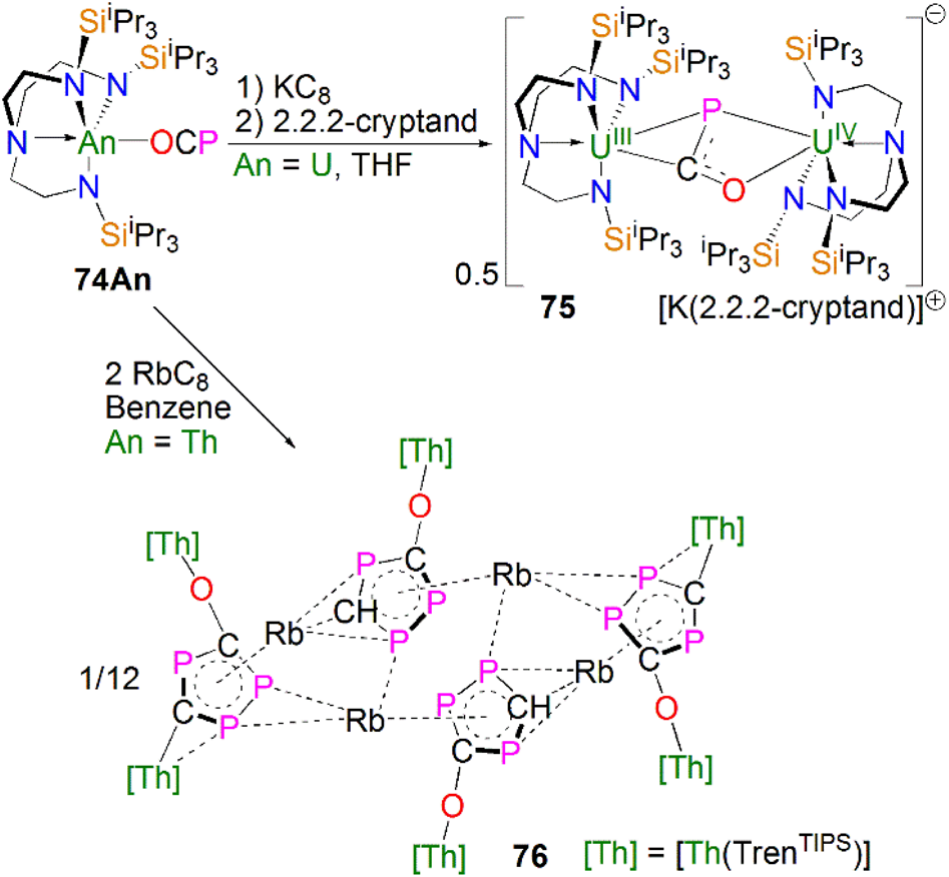
Reduction of actinide–OCP complexes 74An to produce actinide complexes 75 and 76.

Transition metal 2-phosphaethynolate (OCP)^−^ complexes have proven to be useful precursors to prepare phosphido complexes *via* redox or photolysis process.^31^ Recently, Liddle, Scheer, and co-workers reported the two new actinide–OCP complexes [An(Tren^TIPS^)(OCP)] (74An, An = Th and U) and their reduction chemistry.^109,110^ Reduction of 74U with KC_8_ in the presence of 2.2.2-cryptand gives [K(2.2.2-cryptand)][{U(Tren^TIPS^)}_2_{μ-η^2^(OP):η^2^(CP)-OCP}] (75), Scheme 25.^109^ Although there was no phosphido species isolated from this reduction, the coordination mode of this trapped OCP-ligand is unique, and derives from a novel highly reduced and bent carbene-like form of this ligand with a bridging P-centre and the most acute P–C–O angle of ∼127° in any complex to date. The mixed valence diuranium(iii/iv) formulation is supported by the characterisation data and DFT calculations, where back-bonding from uranium gives a highly reduced form of the OCP unit that is perhaps best described as a uranium stabilised (OCP)^2−^ radical dianion. In contrast, reduction of 74Th with KC_8_ or CsC_8_ produced the phosphinidiide C–H bond activation product [{Th(Tren^TIPS^)}Th{N(CH_2_CH_2_NSiPr^i^_3_)_2_[CH_2_CH_2_SiPr^i^_2_CH(Me)CH_2_C(O)μ-P]}] and the oxo complex [{Th(Tren^TIPS^)(μ-OCs)}_2_]. Surprisingly, using RbC_8_ for the reduction afforded a hexathorium complex, [{Th(Tren^TIPS^)}_6_(μ-OC_2_P_3_)_2_(μ-OC_2_P_3_H)_2_Rb_4_] (76), which contains four five-membered [C_2_P_3_] phosphorus heterocycles *via* a [2 + 2 + 1] cycloaddition, Scheme 25.^110^ In addition, this hexathorium complex can be converted to the oxo complex [{Th(Tren^TIPS^)(μ-ORb)}_2_] and the known cyclometallated complex 65Th at 80 °C, *via* an otherwise hidden example of reductive cycloaddition reactivity in the chemistry of 2-phosphaethynolate. From the above examples it can be seen that the reduction chemistry of 2-phosphaethynolate for actinides can be quite complicated.

### 5.6 Actinide polyphosphorus complexes

**Fig. 8:**
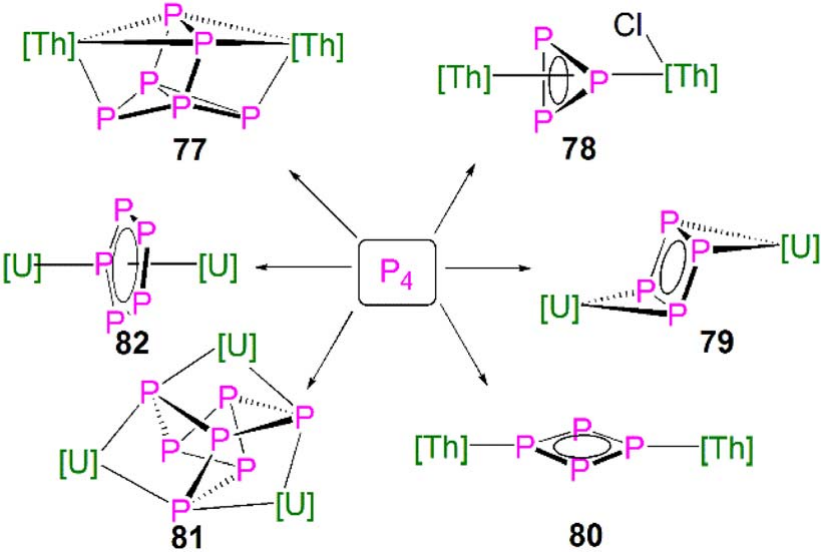
Core structures for reported actinide polyphosphorus complexes 78–82 prepared from P_4_.

Similar to most polyphosphorus complexes, actinide polyphosphorus complexes are most often prepared from P_4_ and low-valent actinide compounds.^20^ In 1991, Scherer and co-workers reported the first examples of actinide polyphosphorus complexes, [{Th(Cp^tt^)_2_}_2_(μ^2^-η^4^-P_6_)] (77) and [{Th(Cp^tt^)_2_}(μ^2^-η^3^-P_3_){Th(Cp^tt^)_2_Cl}] (78), prepared by treatment of [Th(Cp^tt^)_2_(η^4^-C_4_H_6_)] with P_4_ at 100 °C in the absence, or presence of, MgCl_2_, respectively, Fig. 8.^111^ The solid-state structures of 77 and 78 revealed distinct structural differences: a bicyclic P_6_^4−^ ligand is bridged by two thorium(iv) centres in 77, whilst *cyclo*-P_3_^3−^ is bridged in 78, with Th–P bond distances between 2.840(7) and 2.921(7) Å. The ^31^P NMR spectrum for 77 exhibits resonances at 125.3, 18.4 and −41.9 ppm. In contrast, the ^31^P NMR spectrum for 78 shows temperature-dependent features; at room temperature, there is one resonance at −75.7 ppm, however, at 193 K, two resonances at −69.7 and −94.5 ppm were observed, indicating two unique phosphorus environments at low temperature.

This area progressed little over 20 years until in 2011 Cloke, Green and co-workers isolated the *cyclo*-P_4_ uranium complex [{U(Cp*)(C_8_H_6_(Si^i^Pr_3_)_2_)}_2_(μ^2^-η^4^-P_4_)] (79), Fig. 8, from the reaction of the U(iii) complex [U(Cp*)(C_8_H_6_(Si^i^Pr_3_)_2_)(THF)] with half an equivalent of P_4_.^112^ The molecular structure of 79 revealed a diuranium structure where each of the uranium metal centres interacts with the *cyclo*-P_4_ ligand in an η^2^-fashion. The P_4_ ligand forms a chair-like structure with the two uranium atoms, with U–P bond distances of 2.9763(12) and 2.9773(12) Å, respectively, which are typical single bond character. In addition, the P–P bond distances of 2.152(2) and 2.149(2) Å within the bridging *cyclo*-P_4_ unit suggest the dianionic charge of the P_4_ ligand. There is only a single resonance at 718 ppm in the ^31^P{^1^H} NMR spectrum of 79. In 2016, Mills and co-workers reported another actinide *cyclo*-P_4_ complex, [{Th(Cp′′)_3_}_2_(μ^2^-η^2^-P_4_)] (80), Fig. 8, by reacting the Th(iii) complex [Th(Cp′′)_3_] with P_4_.^113^ The solid-state structure of 80 revealed a planar (P_4_)^2−^ ligand bridging two thorium centres *via* two individual η^1^ bonding interactions. The Th–P bond distances of 2.919(4) Å indicates Th–P single bond interactions and the short P–P bond distances of only 2.051(9) Å are 0.1 Å shorter than those in 79, suggesting that the bonding within the *cyclo*-P_4_ ligand is closer to double bond character, which might be due to the η^1^ coordination mode and the lack of Th–P π-bonding. The ^1^H NMR spectrum was diagnostic of a diamagnetic complex, reflecting that the reduction of the P_4_ arises from the oxidation of the Th(iii) precursor to two Th(iv) centres; the ^31^P NMR spectrum of 80 suggests the presence of two different phosphorus environments with two triplet signals at 227.59 and 328.86 ppm, respectively, with a P–P coupling constant of *ca*. 400 Hz.

In 2013, Liddle and co-workers reported that reaction of the diuranium(v) arene complex [{U(Ts^Tol^)}_2_(μ_2_-η^6^:η^6^-C_6_H_5_CH_3_)] (Ts^tol^ = HC(SiMe_2_NAr), Ar = 4-MeC_6_H_4_) with one equivalent of P_4_ resulted in the formation and isolation of the triuranium Zintl cluster [{U(Ts^Tol^)}_3_(μ^3^-η^3^-P_7_)] (81),^114^Fig. 8. The solid-state structure of 81 revealed a P_7_^3−^ trianion cluster capped on three of its faces by three TS^Tol^-uranium cation fragments. The U–P bond lengths of 81 are in the range of 2.949(2)–3.031(2) Å, which are comparable to the U–P cluster distances discussed above. Interestingly, 81 reacts with a variety of halide reagents, such as Me_3_SiCl, LiCl, MeI, and PhI, to afford P_7_R_3_ products with subsequent reduction of the U component closing the synthetic cycle.

Extending the reactivity of P_4_ with other U(iii) complexes, Liddle and co-workers reported that treatment of [U(Tren^TIPS^)] with 0.25 equivalents of P_4_ reproducibly gives the actinide inverted sandwich *cyclo*-P_5_ complex [{U(Tren^TIPS^)}_2_(μ-η^5^:η^5^-*cyclo*-P_5_)] (82),^115^Fig. 8. All previous examples of *cyclo*-P_5_ complexes were stabilised by transition metals, but the isolation of 82 indicates that *cyclo*-P_5_ can also be stabilised by hard actinide ions. Moreover, the characterisation data are consistent with 82 being a diuranium(iv) complex, and thus the *cyclo*-P_5_ unit in 82 is formally a radical dianion rather than the usual monoanion form. The molecular structure of 82 revealed quite long U–P bond distances spanning the range 3.250(6)–3.335(6) Å, which are longer than the sum of the single bond covalent radii of U and P (2.81 Å), perhaps owing to the sterically demanding nature of Tren^TIPS^ ligands combined with the bridging η^5^-bound (per U) nature of the *cyclo*-P_5_ unit in 82. DFT studies on 82 indicates the principal bonding in the U(P_5_)U unit is polarised δ-bonding, whilst the isolobal cyclopentadienyl ligand normally interacts with metals *via* σ- and π-bonding interactions with minimal δ-interaction. In a related study, Zhu, Maron, and co-workers investigated the reactivity of P_4_ with U(iii) supported by a tertiary phosphine-appended Tren ligand, resulting in a diuranium product containing a P_4_ chain.^116^

**Scheme 26:**
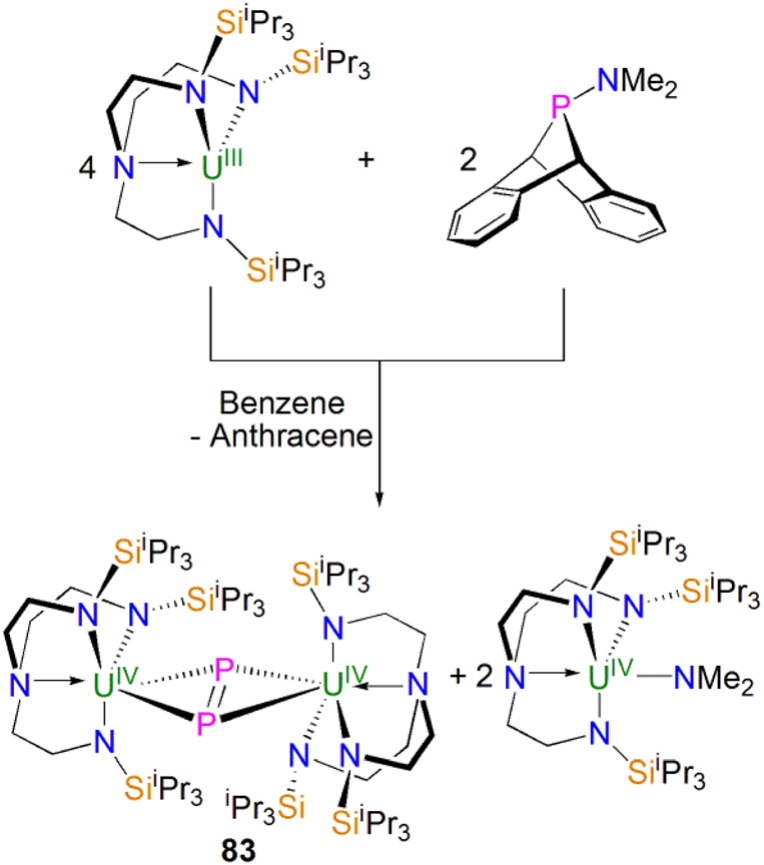
Synthesis of uranium diphosphorus complex 83.

In 2021, Liddle and co-workers reported the synthesis and structure of a side-on bound diphosphorus U(iv) complex, [{U(Tren^TIPS^)}_2_(μ-η^2^:η^2^-P_2_)] (83), Scheme 26, by reacting the 7λ^3^-(dimethylamino)phosphadibenzonorbornadiene P-atom transfer reagent (anthracene-PNMe_2_) with [U(Tren^TIPS^)].^117^ The by-product, [U(Tren^TIPS^)(NMe_2_)], was isolated from the reaction mixture by fractional crystallisation, accounting for the fate of the NMe_2_ unit. Complex 83 is the first diphosphorus complex for any f-element complex, coming after the first f-element dinitrogen complex in 1988.^118^ The molecular structure of 83 revealed P–P bond distances of 2.036(2) Å, indicative of P=P double bond character and hence a dianionic P_2_^2−^, which is consistent with a diuanium(iv) formulation confirmed by the characterisation data. The U–P distances of 2.9441(12) and 2.9446(12) Å are longer than the sum of the single bond covalent radii of U and P (2.81 Å), reflecting the side-on bridging mode of the P_2_ unit in 83. Computational results indicated that within the UP_2_U motif the in-plane U–P π-bonding dominates with a very weak δ-interaction. It was subsequently found that oxidation of 64M with AgBPh_4_ also produces 83 (along with 54U), which suggests the formation of a transient U(v)=PH linkage that disproportionates to 54U and U(vi)≡P, the latter of which could dimerise to give the more stable P–P coupled 83. In addition, a preliminary reactivity study demonstrated that 83 can be converted to uranium *cyclo*-P_3_ complexes [M(arene)_4_][{U(Tren^TIPS^)_2_(μ-η^3^:η^3^-P_3_)] (M = K, Rb, Cs; arene = toluene or benzene); these reactions are low yielding, implying the presence of reactive phosphido intermediates.^107^ DFT calculations indicate that uranium moves from π-bonding to P_2_ and *cyclo*-P_3_ to δ-bonding with *cyclo*-P_5_, highlighting the flexibility of the chemical bonding of uranium.

## 6. Lanthanide heavier pnictogen complexes

Upon descending group 15, the number of f-element heavier pnictogen bonds falls away rapidly, and lanthanide complexes containing heavier pnictogen ligands from As to Bi are much rarer than P analogues. Furthermore, such complexes tend to form multi-centre clusters with bridging pnictogen ligands. Thus, well-defined mononuclear species are sparse. To the best of our knowledge, there are no lanthanide complexes isolated to date with multiple bonding to As, Sb, or Bi.

### 6.1 Lanthanide arsenic complexes

**Fig. 9:**
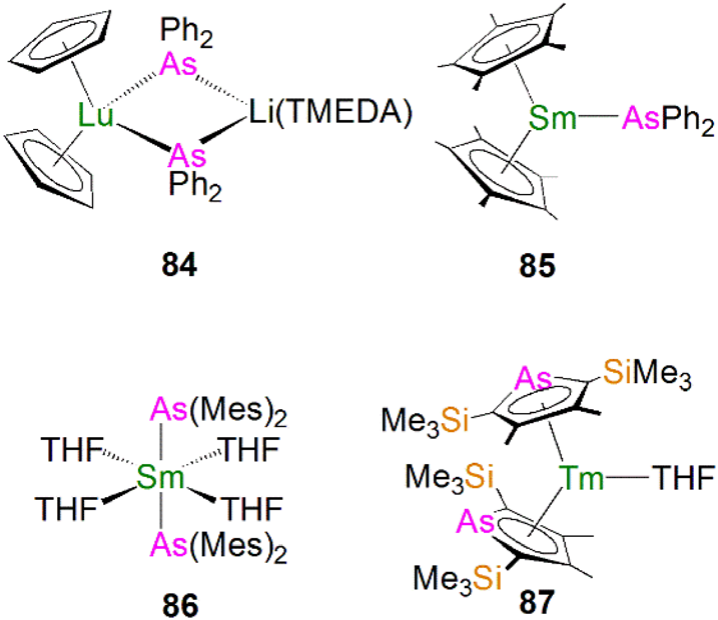
Selected examples of lanthanide complexes containing metal–arsenide bonds 84–87.

The first crystallographically characterised complex containing a lanthanide–arsenic bond was reported in 1988 by Schumann and co-workers. It was found that [Lu(Cp)_2_(μ-CH_3_)_2_{Li(TMEDA)}] (TMEDA = *N*,*N*,*N*′,*N*′-tetramethylethylenediamine) reacted with diphenylarsine in benzene to afford the lanthanide–arsenide complex [Lu(Cp)_2_(μ-AsPh_2_)_2_{Li(TMEDA)}] (84), Fig. 9, *via* methane elimination.^119^ The solid-state structure of 84 revealed the Lu–As bond distances are 2.896(2) and 2.870(2) Å, with a As–Lu–As bond angle of 81.14(6)°. By using the reducing nature of Sm(ii), Evans and co-worker prepared [Sm(Cp*)_2_(AsPh_2_)] (85), Fig. 9, by the reaction of two equivalents of [Sm(Cp*)_2_] with Ph_2_AsAsPh_2_*via* reductive cleavage.^120^ This strategy also works for making the phosphide analogue. When dissolved in THF, 85 converts to the THF adduct [Sm(Cp*)_2_(AsPh_2_)(THF)], which can ring open THF to produce [Sm(Cp*)_2_{O(CH_2_)_4_AsPh_2_}(THF)] under thermolysis conditions. In the molecular structure of 85, there are two independent molecules in unit cell, and the Sm–As bond distances are 2.973(3) and 2.966(3) Å, respectively, which are slightly longer than those seen in 84. Apart from these Ln(iii) arsenide complexes, there are two Ln(ii) complexes containing metal–arsenic bonds, where Nief and co-workers reported the preparation of [Sm(AsMes_2_)_2_(THF)_4_] (86)^36^ and [Tm(Dsas)_2_(THF)] (87, Dsas = 2,5-bis(trimethylsilyl)-3,4-dimethylarsolide)^121^*via* salt metathesis, Fig. 9.

**Scheme 27:**
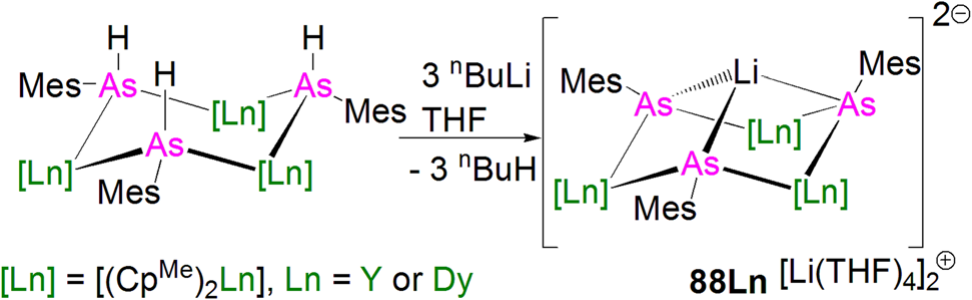
Synthesis of the yttrium and dysprosium arsinidiide complexes 88Ln.

During 2015 and 2016, Layfield and co-workers synthesised the bridging arsinidiide lanthanide complexes [{Ln(Cp^Me^)_2_}_3_(μ-AsMes)_3_Li][Li(THF)_4_]_2_ (88Ln; Ln = Y, Dy; Cp^Me^ = C_5_H_4_Me; Mes = mesityl), Scheme 27, when investigating the effects of arsinidiide ligands on SMM properties.^122,123^ Deprotonation of the bridging lanthanide arsenides [{Ln(Cp^Me^)_2_}_3_(μ-AsHMes)_3_] (Ln = Y or Dy), with three equivalents of *n*-butyl-lithium gave 88Y and 88Dy, in yields of 73% and 77%, respectively, in addition to three equivalents of butane gas. Complexes 88Ln feature bridging pnictide units, similar to the phosphinidiide clusters 28Ln. The central Ln_3_As_3_ core shows a chair-like arrangement that is analogous to the Ln_3_P_3_ cores in 28Ln. The authors reported that 88Ln exhibit Ln–As bond distances (Y: 2.8574(6)–2.8893(7) Å; Dy: 2.8515(6)–2.8908(7) Å) that are shorter than in their arsenide counterparts [{Ln(Cp^Me^)_2_}_3_(μ-AsHMes)_3_](Ln = Y, 2.977(2)–3.019(2) Å; Dy, 2.984(2)–3.009(2) Å). Computational studies of 88Y indicated that the Y–As bonding is ionic with a small amount of covalent character, which is greater than the component seen for the yttrium arsenide precursor. The authors reported that 88Dy demonstrated SMM behaviour at low temperatures (<5 K) with a U_eff_ value of 23(2) cm^−1^ and magnetic hysteresis observed up to 1.8 K. Although the structurally authenticated terminal phosphinidene complex 40 was reported very recently, an analogous lanthanide terminal arsinidene complex still remains elusive.

**Scheme 28:**
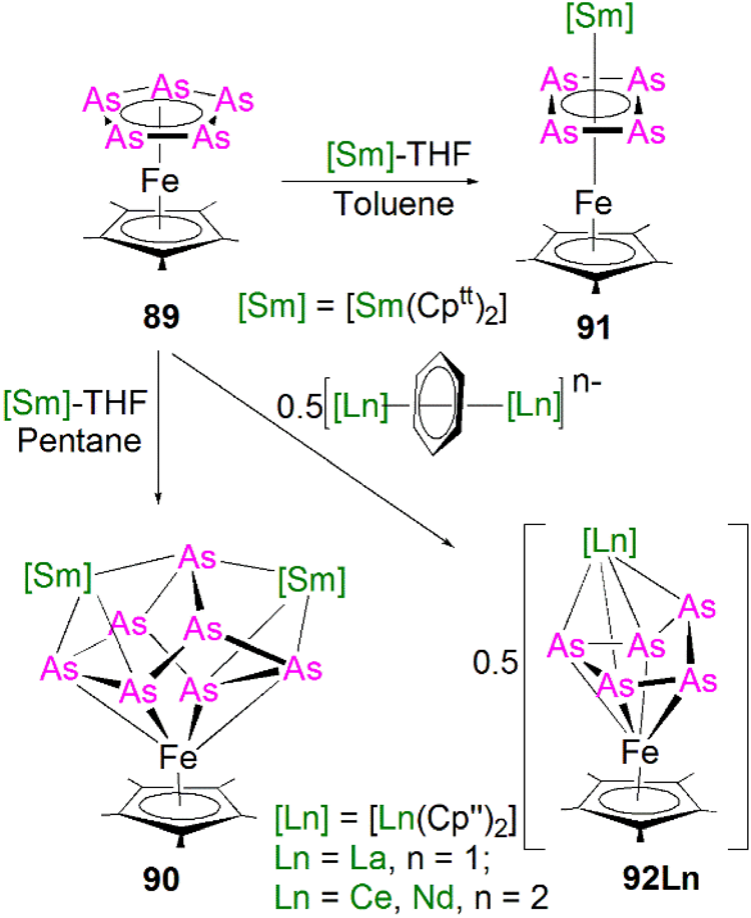
Synthesis of lanthanide polyarsenide complexes 90-92Ln.

Due to synthetic difficulties, the chemistry of lanthanide polyarsenide complexes progressed rather slowly until the last decade. In 2016 Roesky and co-workers showed that treatment of [Fe(Cp*)(η^5^-As_5_)] (89) with [Sm(Cp^tt^)_2_(THF)] gave the first examples of lanthanide polyarsenide complexes, [Sm(Cp′′)_2_(μ-As_7_)Fe(Cp*)] (90) and [Sm(Cp′′)_2_(μ-η^4^-η^4^-As_4_)Fe(Cp*)] (91) by using different solvents, Scheme 28.^124^ The solid-state structures of 90 and 91 revealed hetero-trinuclear and -dinuclear 3d/4f clusters with the As_7_ and As_4_ ligands bridged between multiple metal centres. Complex 90 exhibits a norbornadiene-like structure with two short As–As bonds in the scaffold, whilst 91 is also the first 3d/4f-triple decker sandwich complex with a purely inorganic ligand middle deck. DFT calculations and physical characterisation data indicated that the central As_4_ ligand is dianionic, which is isolobal with the 6p-aromatic cyclobutadiene dianion [C_4_H_4_]^2−^. This is consistent with the As–As bond distances within the *cyclo*-As_4_ ligand in 91, which were found to be in between an As–As single and double bond.

More recent work from the Roesky group has proved that a redox strategy, using [Fe(Cp*)(η^5^-As_5_)] as an arsenic ligand source to react with low-valent lanthanide starting materials, is an effective way to synthesise new lanthanide polyarsenide complexes. Three new 3d/4f polyarsenide complexes in the separate ion pair form [K(18C6)][Ln(Cp′′)_2_(μ-η^4^:η^4^-As_5_)Fe(Cp*)] (92Ln, Ln = La, Ce, Nd) were prepared by the reduction of [Fe(Cp*)(η^5^-As_5_)] with formally low-valent bridging arene lanthanide compounds in moderate yields, Scheme 28.^125^ The molecular structures of 92Ln are very similar to each other, containing highly reduced As_5_ units with an envelope shape. In 92Ln the Fe centre is η^4^-coordinated by the *cyclo*-As_5_ unit and the shortest As–As bond distance of 2.3781(6) Å is observed, while the rest of the As–As bonds range from 2.3928(5) Å to 2.4325(5) Å. As expected, Ln–As bond distances vary over a wide range because of the steric constraints and anisotropic charge distribution with in the As_5_ ligand. In other work, Roesky and co-workers found that reacting the arsenic source [{Co(Cp′′′)}_2_(μ-η^2^:η^2^-As_2_)_2_] with samarocenes produced two new mixed 3d/4f polyarsenic complexes [{Co(Cp^ttt^)}_2_(μ-As)_4_Sm(Cp^Me4R^)_2_] (R = Me, *n*-propyl), which represent the first examples of lanthanide complexes with open chain-like polyarsenic ligands.^126^

**Scheme 29:**
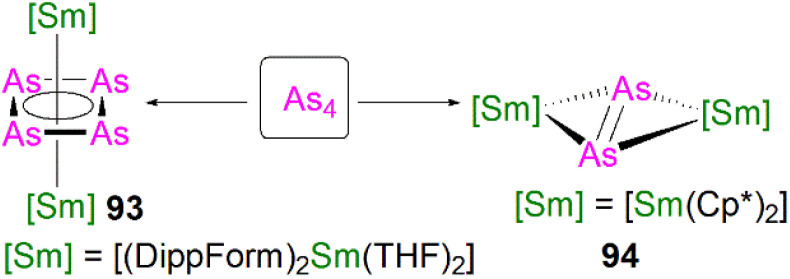
Synthesis of lanthanide polyarsenide complexes 93 and 94.

More recently, Roesky and co-workers found that yellow arsenic (As_4_) is also a useful source to introduce polyarsenic ligands to lanthanides. Due to the unstable nature of this As_4_ allotrope under ambient conditions, the authors used freshly-prepared As_4_ in solution to react with the divalent precursor [Sm(DippForm)_2_(THF)_2_]; after workup, red crystals of the *cyclo*-As_4_ complex [{Sm(DippForm)_2_}_2_(μ-η^4^:η^4^-As_4_)] (93),^61^Scheme 29, were obtained as a minor product, which is essentially isostructural to the lanthanide *cyclo*-P_4_ complex 48. Unfortunately, the presence of non-removable impurities hampered further characterisation on 93 because of the instability of As_4_ in solution. In parallel work, the authors also explored the reactivity of As_4_ with [Sm(Cp*)_2_]. As both reagents are highly reactive and light sensitive, this reaction was performed with the exclusion of light; after work-up, a few single crystals of [{Sm(Cp*)_2_}_2_(μ-η^2^:η^2^-As_2_)] (94), Scheme 29, with inseparable side products were isolated.^127^ The solid-state structure of 94 revealed a rare diarsenic lanthanide species with the As_2_ ligand side-on bound to two samarium centres. The As–As bond distance of 2.278(2) Å indicates As=As double bond character, and hence a dianionic charge on the As_2_^2−^ unit. The Sm–As distances of 3.014(1) Å are statistically the same because of the presence of an inversion centre in 94.

**Scheme 30:**
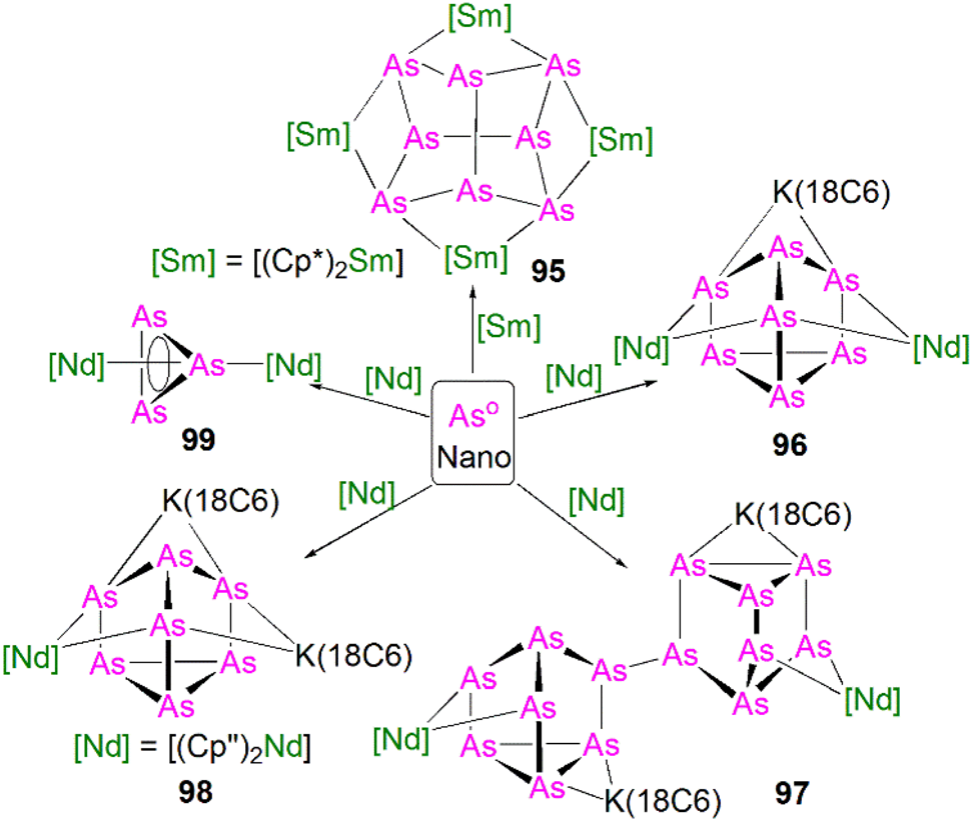
Synthesis of lanthanide polyarsenic complexes 95–99.

The practical difficulties of working with As_4_ solutions has spurred the development of new arsenic starting materials for construction of polyarsenic complexes. Roesky and co-workers reported the preparation for arsenic nanoparticles using a reductive synthetic method developed by Feldmann and co-workers.^128^ The elemental As^0^ nanoparticles (*nano*-As^0^, *d* = 7.2 ± 1.8 nm) can be formed by the reduction of AsI_3_ with a freshly prepared solution of lithium naphthalenide at 0 °C in THF. The *nano*-As^0^ can be isolated in a pure form as the LiI by-product is sufficiently soluble in THF and Et_2_O to be washed away; this has proven very effective at introducing As oligomers to f-elements, Scheme 30. It was reported that 94 could be prepared by the reaction of [Sm(Cp*)_2_] with *nano*-As^0^ at 60 °C, but again with inseparable byproducts and in low yield. Interestingly, under harsh conditions, the tetrasamarium polyarsenide cluster [{Sm(Cp*)_2_}_4_(μ-As_8_)] (95) was reproducibly isolated in yields of 22% as a crystalline solid after work-up.^128^ In the molecular structure of 95, the Sm–As bond distances fall into the small range of 3.0814(10)–3.1734(10) Å, which are slightly longer than those of 94, and the As–As bond distances are between 2.4044(12) Å and 2.5003(12) Å, suggesting single bonds with angles of 93.52(4)° to 103.68(4)° within the [As_8_]^4−^ tetraanionic cage. Very recently, the authors expanded this chemistry, reacting *nano*-As^0^ with the dilanthanide inverted arene complexes [K(18-crown-6)][{Ln(Cp′′)_2_}_2_(μ-η^6^:η^6^-C_6_H_6_)] (Ln = La, Ce) and [[K(18-crown-6)]_2_[{Ln(Cp′′)_2_}_2_(μ-η_6_:η_6_-C_6_H_6_)] (Ln = Ce, Nd) to give a range of lanthanide Zintl anions 96–99 containing As_3_^3−^, As_7_^3−^, and As_14_^4−^ ligands, respectively, that were previously not accessible in molecular lanthanide chemistry, Scheme 30.^129^ The As_14_^4−^ unit in 97 is the largest organo-lanthanide-polyarsenic complex to date. The synthesis and characterisation of these diverse polyarsenic lanthanide complexes demonstrated the great utility of *nano*-As^0^ for accessing novel molecular polyarsenic clusters.

### 6.2 Lanthanide antimony complexes

**Scheme 31:**
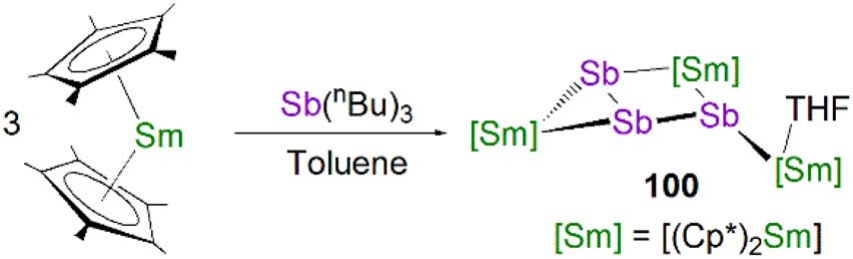
Synthesis of samarium polyantimony complex 100.

Although there have not been any reports of structurally authenticated f-element-antimony or -bismuth multiple bonds, a relatively small number of lanthanide complexes featuring single bond interactions with these heavy pnictogens have been crystallographically characterised. Lanthanide–antimony or -bismuth bonds are commonly synthesised as clusters with the polyantimony or polybismuth ligands stabilised between multiple metal centres. Complexes containing metal–antimony or -bismuth multiple bonding interaction are also rare for d-block metals; to date, there has been only one single example of a terminal metal stibido complex, [W^vi^(Tren^TMS^)(Sb)] (Tren^TMS^ = {N(CH_2_CH_2_NSiMe_3_)_3_}^3−^) with a W≡Sb triple bond (2.526(2) Å), prepared by Scheer and co-workers.^130^ No structurally authenticated metal–bismuth multiple bonds are known both for d-block and f-block metals. In 1992, Evans and co-workers reported the synthesis of a complex featuring Sm–Sb interactions. The reaction of one equivalent each of [Sm(Cp*)_2_] and Sb(^*n*^Bu)_3_ afforded the samarium antimony Zintl ion complex [{Sm(Cp*)_2_}_3_(μ-η^2^:η^2^:η^1^-Sb_3_)(THF)] (100), Scheme 31.^131^ Complex 100 exhibits five Sm–Sb bonds in the range of 3.162(1)–3.205(1) Å.

**Scheme 32:**
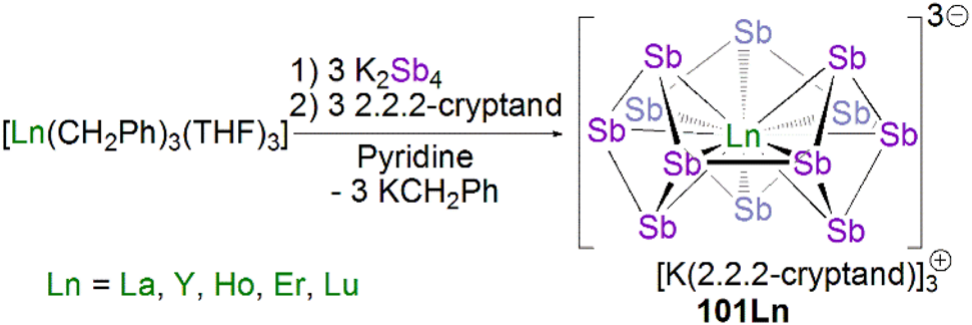
Synthesis of lanthanide Zintl polyantimony complexes 101Ln. Ln–Sb interactions are omitted for clarity.

Surprisingly, there were no further reports of lanthanide–antimony bonds until in 2016 a wide range of lanthanide–polyantimony clusters emerged. Sun, Boldyrev and co-workers reported the synthesis of lanthanide(iii) complexes with three Zintl Sb_4_^2−^ units [K(2.2.2-cryptand)]_3_[Ln(η^4^-Sb_4_)_3_] (101Ln; Ln = La, Y, Ho, Er, Lu).^132^ Complexes 101Ln were synthesised *via* the reaction of [Ln(CH_2_C_6_H_5_)_3_(THF)_3_] (Ln = La, Y, Ho, Er, Lu) with three equivalents each of K_2_Sb_4_ and 2.2.2-cryptand in pyridine, Scheme 32. The five complexes exhibit two types of Ln–Sb bond distances, with equatorial Ln–Sb_eq_ distances of ∼3.4 Å and shorter non-equatorial Ln–Sb_ne_ distances of ∼3.2 Å. The authors noted that the Ln–Sb bond distances decrease from lanthanum to lutetium due to the lanthanide contraction (Ln–Sb_ne_: 3.2461(5), 3.0932(10), 3.0843(9) and 3.0643(11) Å for La, Ho, Er and Lu respectively). Calculations on 101Ln indicated that the Sb_4_ units are aromatic in nature, similar to cyclobutadienyl.

**Scheme 33:**
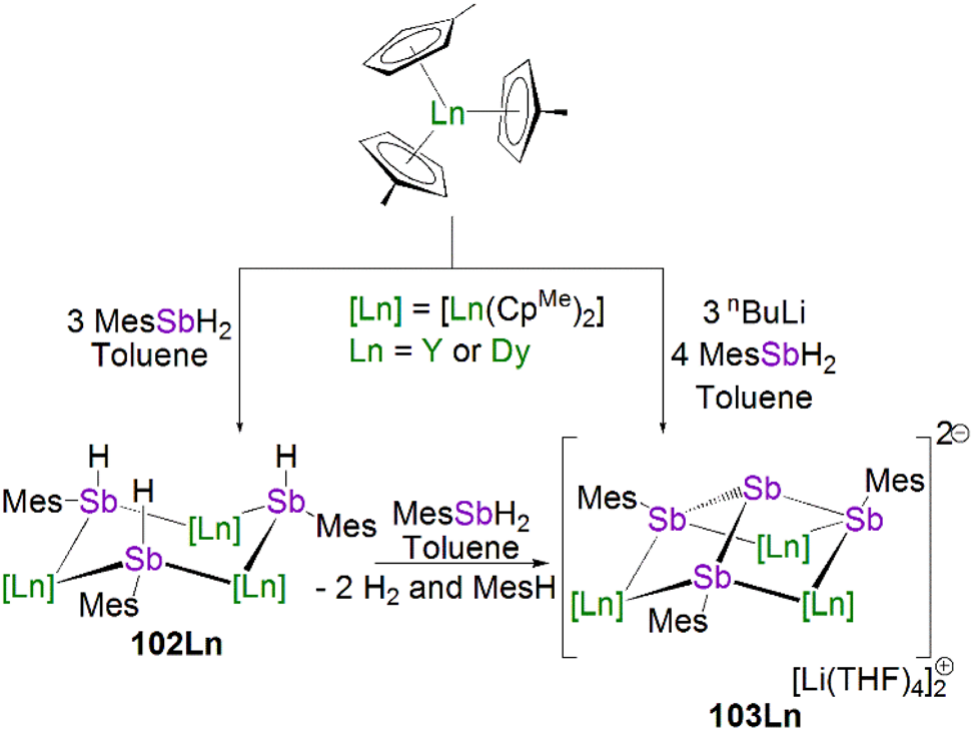
Synthesis of lanthanide polyantimony complexes 102Ln and 103Ln.

In 2017, Layfield and co-workers reported the synthesis of lanthanide stibide complexes which contain either a bridging stibide or a Zintl-like [Sb_4_Mes_3_]^3−^ moiety. The bridging stibide [{Ln(Cp^Me^_2_)}_3_(μ-SbHMes}_3_)] (102Ln, Ln = Y, Dy) was synthesised *via* the reaction of three equivalents each of [Ln(Cp^Me^)_3_] and MesSbH_2_, Scheme 33.^133^ Complexes [{Ln(Cp^Me^_2_)}_3_{μ-(SbMes)_3_Sb}] (103Ln, Ln = Y, Dy) were synthesised *via* two different routes, the first of which involved the reaction of three equivalents each of [Ln(Cp^Me^)_3_] and *n*-butyl lithium with four equivalents of MesSbH_2_; however, stibine dehydrocoupling to give the distibane (Sb_2_H_2_Mes_2_) and tetrastibetane (Sb_4_Mes_4_) can occur. The second reported route to 103Ln involved the cross-dehydrocoupling reaction of one equivalent of the stibide precursor 102Ln with one equivalent of MesSbH_2_ to give one equivalent each of 103Ln (Ln = Y, quantitative; or Dy, 45%) and mesitylene, and two equivalents of hydrogen gas. Complexes 103Ln exhibit solid state structures with Ln_3_Sb_3_ cores akin to the arsinidiide 88Ln and phosphinidiide 28Ln analogues, however in 103Ln the Sb_3_ unit is capped with a Sb^3−^ fragment to form a Zintl-like moiety. Complexes 103Ln feature mean Y–Sb and Dy–Sb bond distances of 3.1211(15)–3.1420(16) Å, and 3.119(1)–3.138(1) Å, respectively. These bond distances are very similar to that of the stibide precursors 102Ln, which exhibit Ln–Sb bond distances of 3.0987(11)–3.2008(6) and 3.092(6)–3.212(3) Å for Y and Dy, respectively. This observation differs from the analogous pnictinidiide complexes 28Ln and 88Ln, where the Ln–Pn distances are shorter than the pnictide precursors; this is likely due to 103Ln exhibiting [Sb_4_Mes_3_]^3−^ units as opposed to discrete RSb^2−^ stibinidene ligands. The authors reported that both 102Dy and 103Dy show SMM behaviour, with U_eff_ values of 345 and 270 cm^−1^, respectively, and both complexes exhibiting magnetic hysteresis at 1.8 K.

**Scheme 34:**
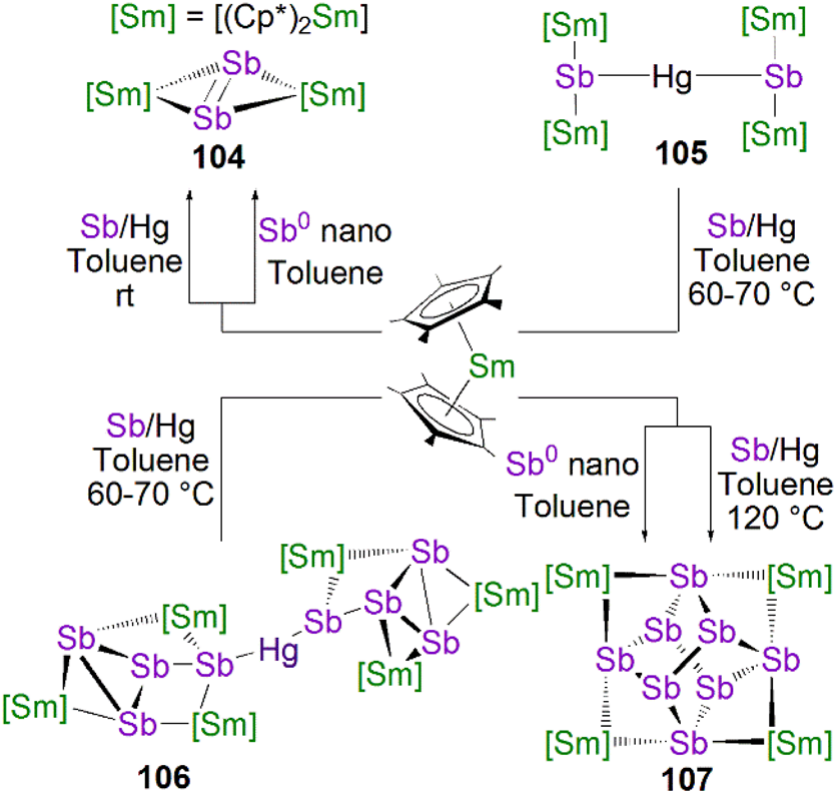
Synthesis of samarium polyantimony complexes 104–107.

In 2018, Roesky and co-workers utilised [Sm(Cp*)_2_] to stabilise a range of samarium polyantimony complexes, Scheme 34.^134^ The reaction of [Sm(Cp*)_2_] with an Sb/Hg amalgam in toluene yielded a mixture of the polyantimony complexes [{Sm(Cp*)_2_}_2_(μ-η^2^:η^2^-Sb_2_)] (104) and [{[Sm(Cp*)_2_]_2_Sb}_2_(μ-Hg)] (105), Scheme 34. The product was dependent upon the temperature of the reaction; whilst at room temperature the primary products were 104 and 105, if the reaction mixture was heated at 60–70 or 120 °C, the polyantimony complexes [{[Sm(Cp*)_2_]_3_(Sb_4_)}_2_Hg] (106) or [{Sm(Cp*)_2_}_4_(Sb_8_)] (107) were isolated, respectively. Using a Sb/Hg amalgam however resulted in difficult to separate antimony products, so the authors reported an alternate synthetic route to both 104 and 107*via* the reaction of [Sm(Cp*)_2_] with antimony nanoparticles. Complex 104 exhibits an Sm–Sb bond distance of 3.2141(9) Å, which is comparable to complex 100, whereas complex 105 demonstrates significantly shorter Sm–Sb distances of 3.0052(14)–3.0158(13) Å. Complexes 106 and 107 exhibit longer Sm–Sb bond distances of 3.2238(8)–3.3694(8) Å and 3.3134(8)–3.4119(7) Å, respectively. More recently, Roesky and co-workers have shown that [Sm(Cp*)_2_] can also be used to make 4d/4f polyantimony clusters containing a planar Sb_4_-unit that is similar to that seen in 91.^135^

### 6.3 Lanthanide bismuth complexes

**Fig. 10:**
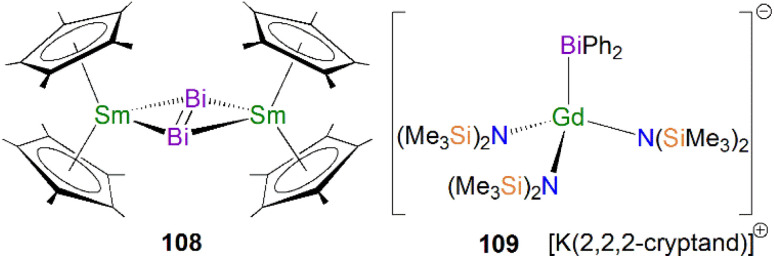
Lanthanide–bismuth complexes 108 and 109.

The first example of a structurally authenticated lanthanide bismuth interaction was reported in 1991 by Evans and co-workers. The reaction of two equivalents of [Sm(Cp*)_2_] with one equivalent of BiPh_3_ in toluene afforded [{Sm(Cp*)_2_}_2_(μ-η^2^:η^2^-Bi_2_)] (108) in yields of up to 60%,^136^Fig. 10. The complex is isostructural to the diantimony complex 104 and exhibits Sm–Bi distances ranging from 3.2645(10) to 3.3108(11) Å, which are longer than that the Sm–Sb distances in 104 (3.2141(9) Å). More recent work from the Evans group has shown that the reaction of BiPh_3_ with the Gd(ii) complex [K(2.2.2-cryptand)][Gd{N(SiMe_3_)_2_}_3_] afforded a monomeric Gd(iii)-bismuthide complex [K(2.2.2-cryptand)][Gd{N(SiMe_3_)_2_}_3_(BiPh_2_)] (109),^137^Fig. 10, which is the only example of a mononuclear lanthanide–bismuth compound. In the molecular structure of 109, the Gd–Bi bond distance of 3.3516(5) Å is very close to the Sm–Bi bond in 108.

**Scheme 35:**
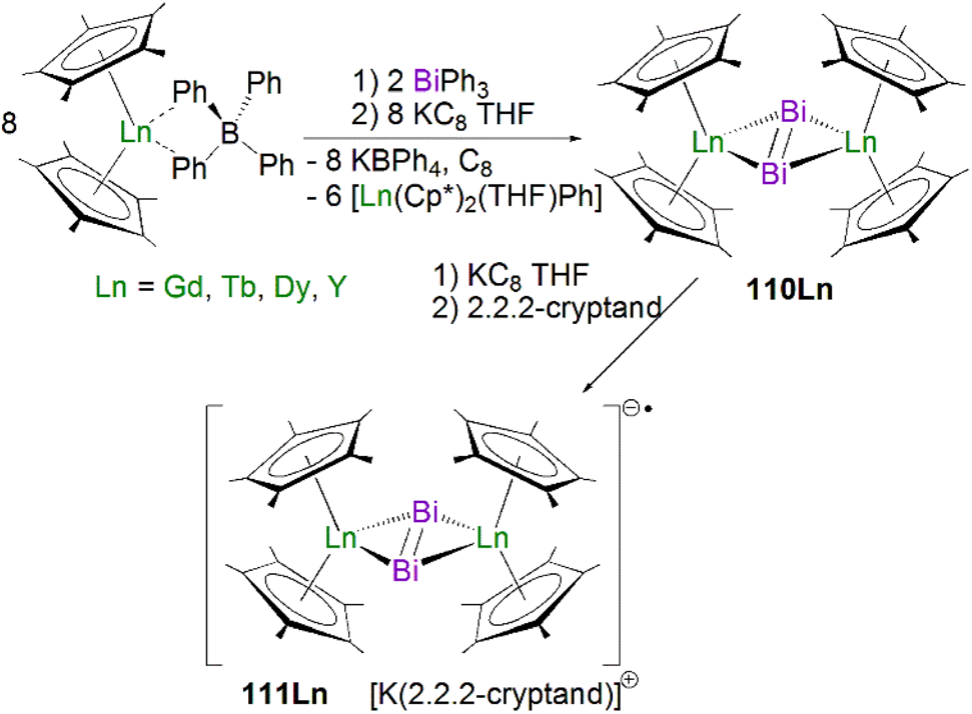
Synthesis of lanthanide dibismuth complexes 110Ln–111Ln.

Very recently, Chilton, Demir and co-workers reported two new sets of lanthanide dibismuth complexes, Scheme 35. The neutral complexes [{Ln(Cp*)_2_}_2_(μ-η^2^:η^2^-Bi_2_)] (110n, Ln = Gd, Tb, Dy, Y) were prepared by the one-pot reactions of eight equivalents of [Ln(Cp*)_2_(BPh_4_)] (Ln = Gd, Tb, Dy, Y), two equivalents of triphenylbismuth, and eight equivalents of KC_8_ at room temperature under argon.^138^ Complexes 110Ln are quite soluble in toluene, thus the poor atom-efficiency of the reaction is overcome by separation from the poorly soluble byproducts. Reduction of 110Ln using KC_8_ in the presence of 2.2.2-cryptand in THF afforded the radical complexes [K(2.2.2-cryptand)][{Ln(Cp*)_2_}_2_(μ-η^2^:η^2^-Bi_2_˙)] (111Ln, Ln = Gd, Tb, Dy, Y).^138^ Complexes 111Ln are the first examples containing the heaviest dinitrogen radical analogues of dibismuth for any d- or f-block metal. The radical nature of 111Ln are confirmed by the characterisation data and DFT calculations. Reflecting the radical electron in the π-antibonding orbital of the Bi_2_ ligand in 111Ln, the Bi–Bi bond distances of (2.9310(11) to 2.9450(13) Å) are longer than those in neutral 110Ln (2.8418(10) to 2.8549(9) Å), and the Ln–Bi bond distances are shorter in 111Ln (3.1865(8) to 3.2064(5) Å) *vs.*110Ln (3.2335(2) to 3.2611(8) Å). Magnetic studies have shown that the Bi_2_^3−^ radical-bridged 111Tb and 111Dy are SMMs with magnetic hysteresis where the Bi_2_^3−^ radical as the bridge engenders antiferromagnetic exchange coupling with the paramagnetic lanthanide ions, leading to a ferrimagnetic ground state.

**Scheme 36:**
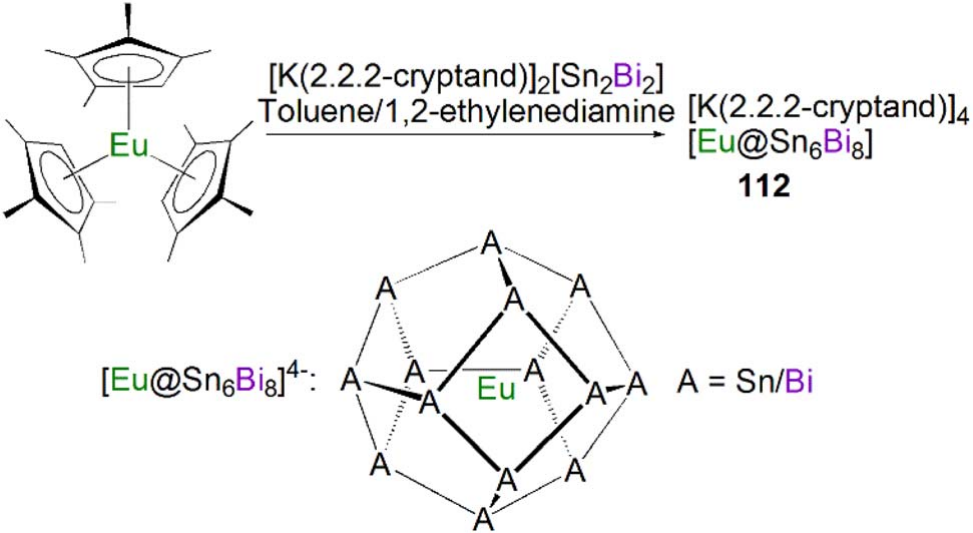
Synthesis of europium polybismuth Zintl anionic clusters 112. Eu–A interactions are omitted for clarity.

Similar to antimony chemistry there have been a range of lanthanide–bismuth interactions stabilised though the isolation of Zintl anions bound to the lanthanide metal ions. In 2011, Dehnen and co-workers utilised this method to synthesise a mini-fullerene-type Zintl-lanthanide complex, Scheme 36. The reaction of [K(2.2.2-cryptand)]_2_[Sn_2_Bi_2_]·en (en = 1,2-ethylenediamine) and [Eu(C_5_Me_4_H)_3_] in toluene gave [K(2.2.2-cryptand)]_4_[Eu@Sn_6_Bi_8_] (112) in a 11% yield.^139^ The solid-state structure of 112 indicates each position of the cage is occupied by either a Sn or Bi atom in a ratio of 0.76–0.11 and 0.24–0.89, respectively. The Eu-(Sn/Bi) bond distances exhibited by 112 span a wide range of 3.3515(8)–3.5770(9) Å. The Sn/Bi atoms in the non-equatorial positions exhibit shorter mean Eu–Sn/Bi distances (3.4743(11) Å) compared to the equatorial distance (3.5208(15) Å). Complex 112 exhibits a short mean axial Eu–Sn/Bi distance of 3.3418(10) Å.

**Scheme 37:**
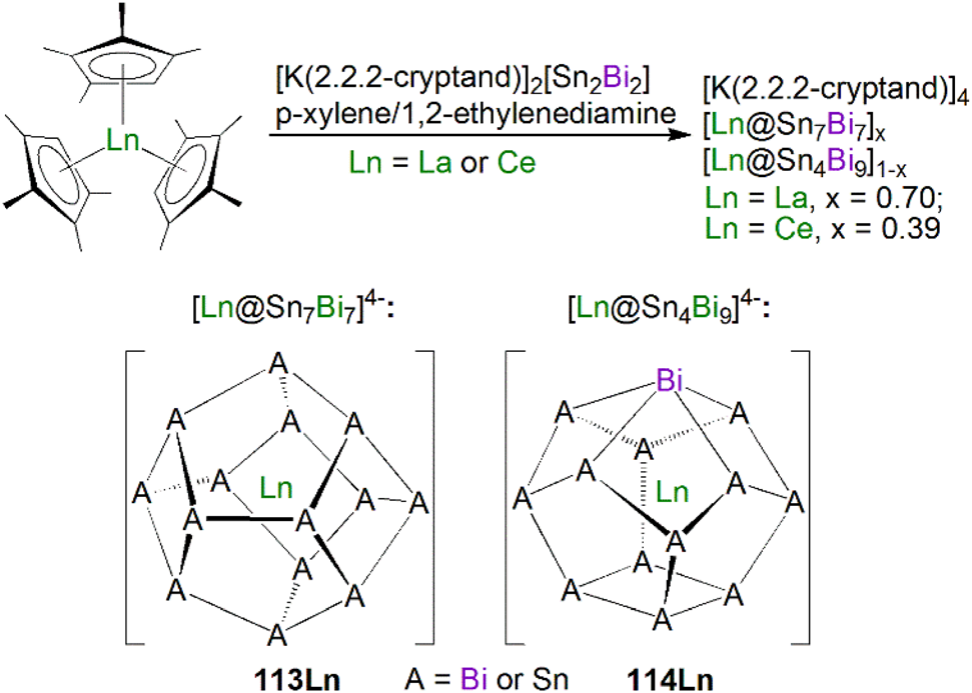
Synthesis of lanthanide polybismuth Zintl anionic clusters 113Ln and 114Ln and representations of the polyhedral architectures. Ln–A interactions are omitted for clarity.

In 2012, Dehnen and co-workers further expanded the range of lanthanide bismuth clusters, Scheme 37; the separate reactions of [K(2.2.2-cryptand)]_2_[Sn_2_Bi_2_]·en with [Ln(C_5_Me_4_H)_3_] (Ln = La or Ce) in *p*-xylene gave mixtures of tin–bismuth clusters surrounding lanthanide centres in [K(2.2.2-cryptand)]_4_[Ln@Sn_7_Bi_7_]_*x*_[Ln@Sn_4_Bi_9_]_1−*x*_ (Ln = La, *x* = 0.70; Ln = Ce, *x* = 0.39).^140^ The crystallographic data for both complexes demonstrate two types of cluster, the 14-vertex anion [Ln@Sn_7_Bi_7_]^4−^ (113Ln) and 13-vertex anion [Ln@Sn_4_Bi_9_]^4−^ (114Ln). Like previous Sn/Bi clusters, anions 113Ln and 114Ln exhibit disorder of the Sn and Bi atoms occupying the same atomic positions in the cluster. The clusters 113Ln have similar structures to 112, exhibiting longer mean Ln–Sn/Bi equatorial distances of 3.539(2) (La) and 3.493(3) Å (Ce) when compared to the non-equatorial distances of 3.433(2) (La) and 3.416(2) Å (Ce). Clusters 113La and 113Ce exhibit mean Ln–Sn/Bi axial distances of 3.426(2) and 3.436(2) Å, respectively. The second cluster type, 114Ln, features Ln–Sn/Bi bond distances over the range of 3.107(4)–3.557(2) and 3.046(12)–3.543(3) Å for La and Ce, respectively. The authors postulated that the differences in Ln–Sn/Bi distances between the La and Ce analogues of 114La and 114Ce is most likely a result of the different ionic radii of La(iii) and Ce(iii).

**Scheme 38:**
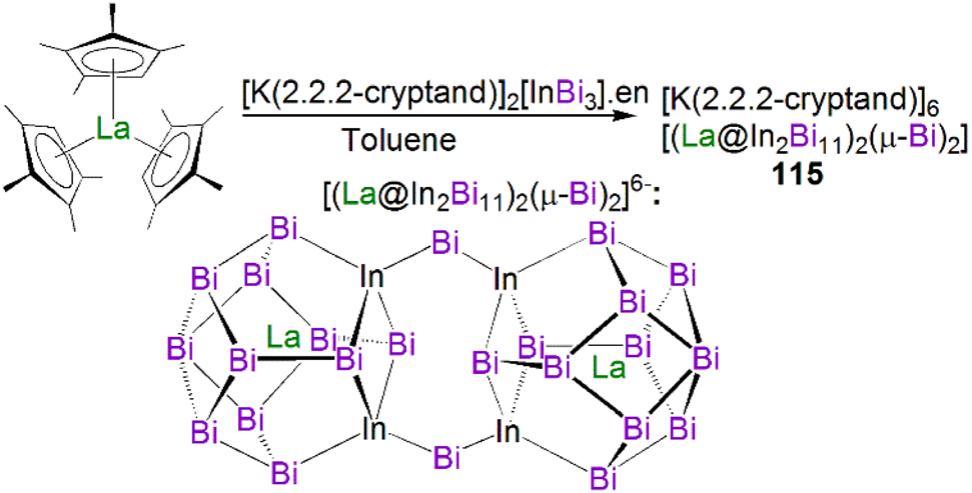
Synthesis of lanthanum polybismuth Zintl anionic clusters 115 and representation of the polyhedral architecture. La–Bi interactions are omitted for clarity.

In parallel work in 2012, Dehnen and co-workers reported the synthesis of the indium metalloid bismuth Zintl cluster, [K(2.2.2-cryptand)]_6_[(La@In_2_Bi_11_)_2_(μ-Bi)_2_] (115) *via* the reaction of [K(2.2.2-cryptand)]_2_[InBi_3_]·en with [La(C_5_Me_4_H)_3_] in toluene in a yield of 11%,^141^Scheme 38. Complex 115 features two 13-vertex anions connected *via* two bridging bismuth atoms. The La–Bi distances exhibited by 115, 3.1343(8)–3.5018(9) Å, are similar to that of the anion 114Ln [3.106(4)–3.557(2) Å].

**Scheme 39:**
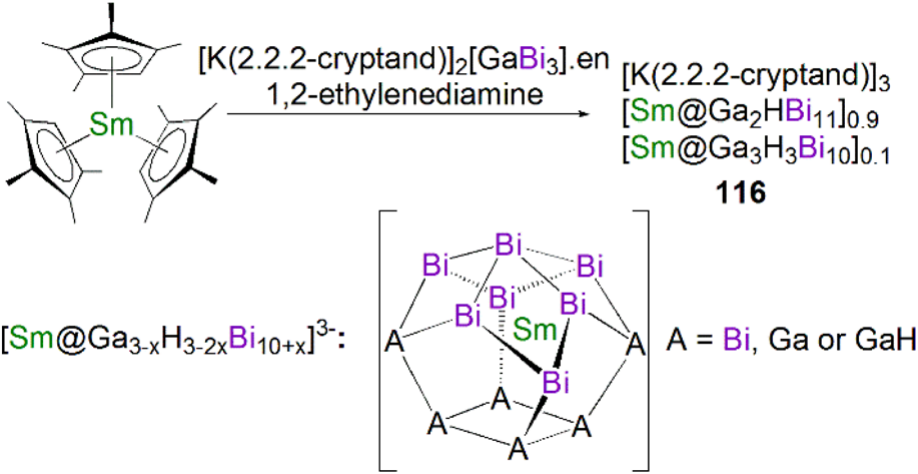
Synthesis of samarium polybismuth Zintl anionic clusters 116. Sm–Bi/A interactions are omitted for clarity.

In 2014, Dehnen and co-workers utilised similar protocols to expand the number of 13-vertex lanthanide–bismuth metalloid clusters to include the gallium-substituted cage [K(2.2.2-cryptand)]_2_[Sm@Ga_2_HBi_11_]_0.9_[Sm@Ga_3_H_3_Bi_10_]_0.1_ (116) *via* the reaction of [K(2.2.2-cryptand)]_2_[GaBi_3_].en with [Sm(C_5_Me_4_H)_3_], Scheme 39.^142^ The authors reported that the two different trianionic fragments feature disorder similar to the tin analogue 113Ln, with several of the atomic positions in the cluster being occupied by Bi, Ga and GaH. Formulation of each anionic cluster was determined by the examination of a combination of techniques, namely single crystal diffraction, energy dispersive X-ray and electrospray ionisation mass spectrometry (ESI-MS), which indicated that the two fragments were present in a ratio of 92% to 8% for [Sm@Ga_2_HBi_11_] and [Sm@Ga_3_H_3_Bi_10_], respectively. The Sm–Bi/Ga distances in 116 span 3.0464(6)–3.4134(6) Å, which is a similar to the other 13-vertex lanthanide cluster complexes 114Ln and 115.

**Scheme 40:**
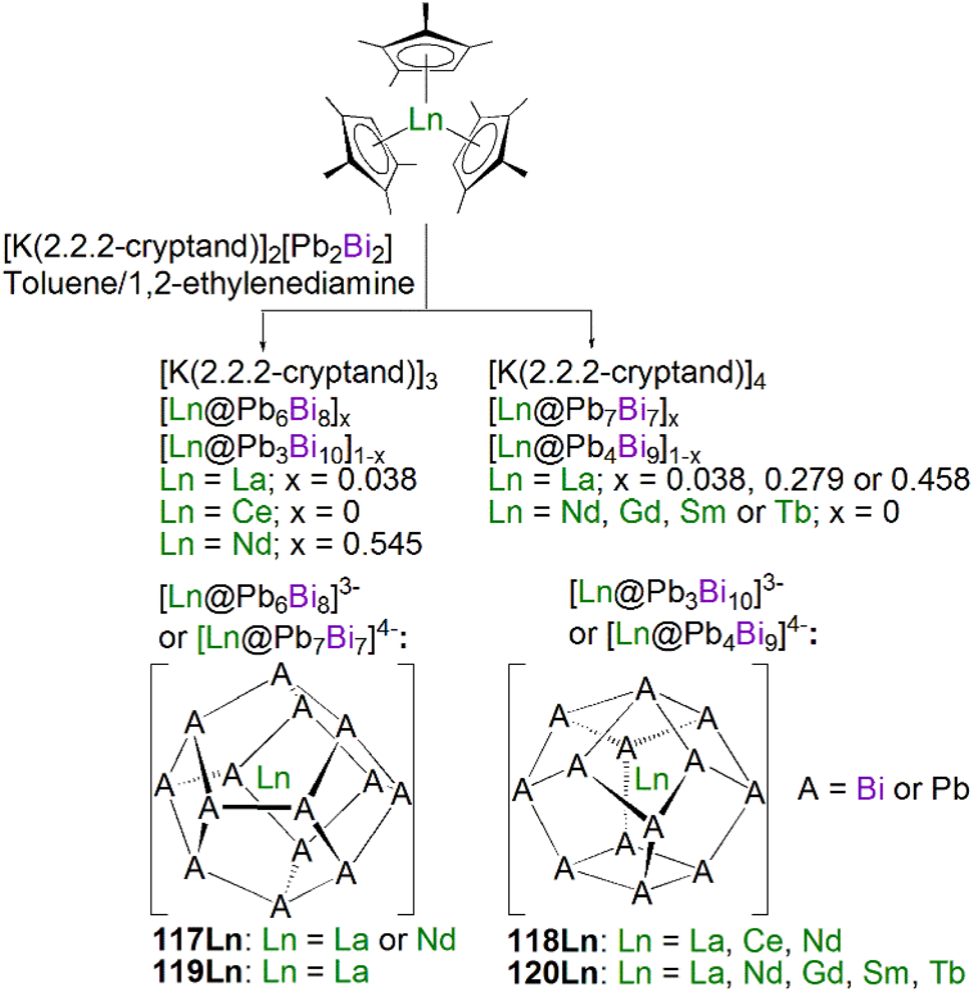
Synthesis of lanthanide polybismuth Zintl anionic clusters 117Ln–120Ln. Ln–A interactions are omitted for clarity.

In 2015, Dehnen and co-workers reported the synthesis of a wide range of lead bismuth cages. The separate reactions of the *in situ*-generated Zintl lead precursor [K(2.2.2-cryptand)]_2_[Pb_2_Bi_2_]·en with [Ln(C_5_Me_4_H)_3_] (Ln = La, Ce, Nd, Gd, Sm, Tb) in toluene yielded ten complexes, [K(2.2.2-cryptand)]_3_[Ln@Pb_6_Bi_8_]_*x*_[Ln@Pb_3_Bi_10_]_1−*x*_, (*x* = 0.038, 0 or 0.545 for La, Ce or Nd respectively) or [K(2.2.2-cryptand)]_4_ [Ln@Pb_7_Bi_7_]_*y*_[Ln@Pb_4_Bi_9_]_1−*y*_ (Ln = La; *y* = 0.038, 0.279 or 0.458; Ln = Nd, Sm or Tb; *y* = 0), depending on the lanthanide used, Scheme 40.^143^ All ten complexes feature a mixture of 14- and 13-vertex anions, [{Ln@Pb_6_Bi_8_}_*x*_{Ln@Pb_3_Bi_10_}_1−*x*_]^3−^ ([117Ln_*x*118Ln1−*x*_]^3−^) and [{Ln@Pb_7_Bi_7_}_*y*_{Ln@Pb_4_Bi_9_}_1−*y*_]^4−^([119Ln_*y*120Ln1−*y*_]^4−^); the structures for the differing anions are analogous to those of 113Ln and 114Ln. Like the previously reported lanthanide bismuth cages (114Ln and 116), the anionic clusters 117Ln–120Ln all contain disorder, resulting in both Pb and Bi occupying the same atomic sites.

**Scheme 41:**
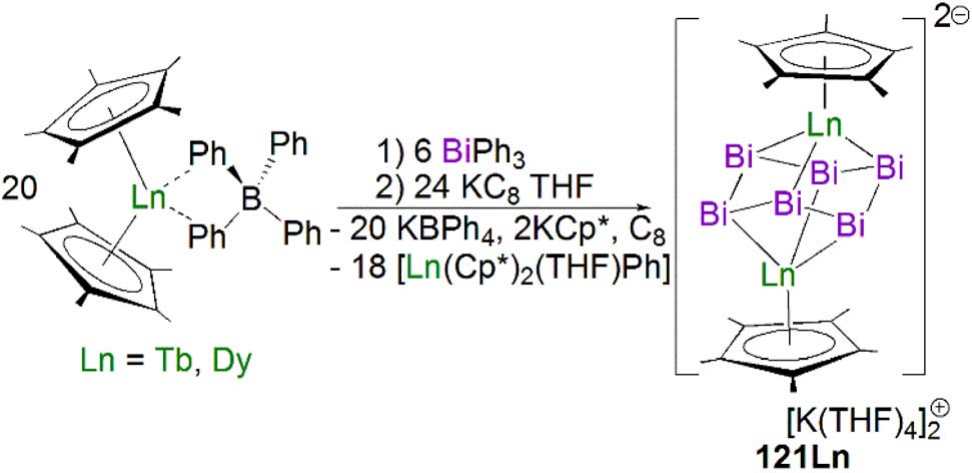
Synthesis of lanthanide polybismuth anionic clusters 121Ln.

In 2021, Demir and co-workers reported the synthesis of [K(THF)_4_]_2_[{Ln(Cp*)_2_}_2_(μ-Bi_6_)] (121Ln, Ln = Tb, Dy) by one-pot reactions of [Ln(Cp*)_2_(BPh_4_)] (Ln = Tb, Dy) with triphenylbismuth in THF, followed by reduction with KC_8_ at 45 °C, Scheme 41.^144^ The authors proposed that the use of KC_8_ induced reduction and bismuth cluster formation. The solid-state structures of 121Ln revealed [Ln_2_Bi_6_] cores with lanthanide centres bridged by a rare [Bi_6_]^6−^ Zintl ion. Notably, complexes 121Ln are the first examples of a cyclic bismuth hexamer in an organometallic complex with any metal. The Ln–Bi bond distances for 121Tb and 121Dy are 3.055(1)–3.070(1) and 3.042(1)–3.060(1) Å, respectively, which are approximately 0.2 Å shorter than that the Sm–Bi bonds in 108 (3.2645(10)–3.3108(11) Å). The Bi–Bi distances of 3.029(1)–3.042(1) and 3.027(1)–3.036(1) A° for 121Tb and 121Dy, respectively, are significantly longer than multiple Bi–Bi bonds (2.82–2.87 A°), and are comparable with Bi–Bi single bonds (>2.99 A°). Magnetic data and quantum calculations indicates strong ferromagnetic interactions between the lanthanide ions facilitated by the Zintl [Bi_6_]^6−^ ligands, resulting in magnetic blocking and open hysteresis loops that are rarely observed for super exchange-coupled SMMs containing solely lanthanide ions.

## 7. Actinide heavier pnictogen complexes

In parallel with lanthanide chemistry, actinide complexes containing heavier pnictogen ligands (from As to Bi) are much rarer than actinide–phosphorus congeners. Again, these complexes tend to form multi-nuclear clusters with the heavier pnictogen ligands bridged by two or more metal centres, thus well-defined mononuclear species are sparse. However, significant advances have been achieved in actinide arsenic multiple bonding chemistry that are discussed in this section. In contrast, there are currently no examples of actinide complexes featuring multiple bonding interactions with antimony or bismuth ligands.

### 7.1 Actinide arsenic complexes

**Fig. 11:**
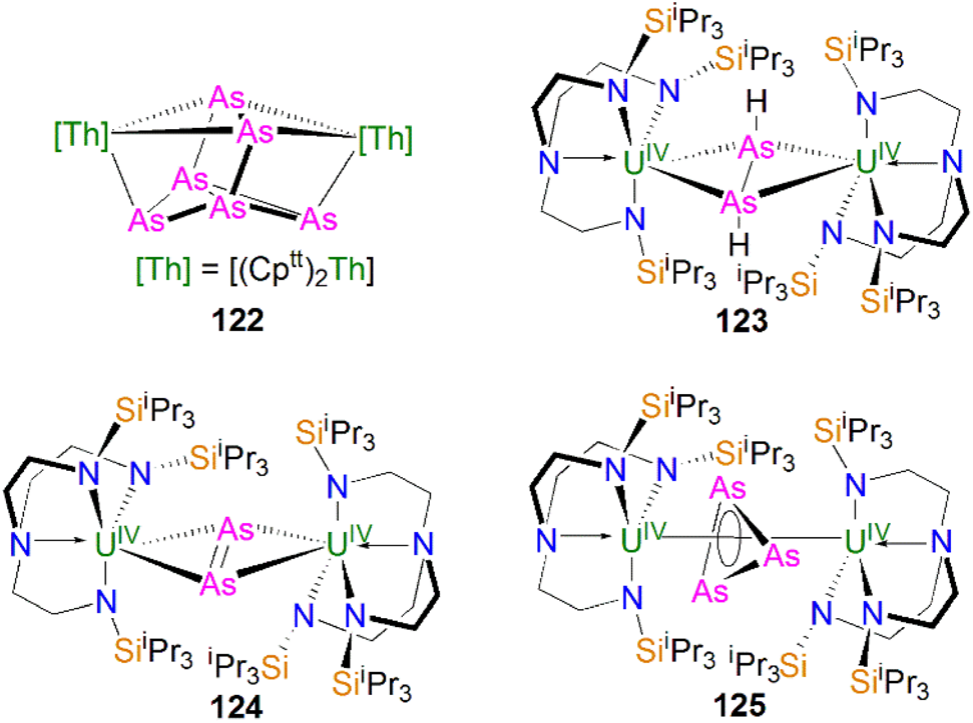
Examples containing actinide–arsenic bonds 122–125.

In 1994, Using a similar synthetic approach used for the preparation of 77, Scherer and co-workers synthesised the first examples of an actinide polyarsenic complex [{Th(Cp^tt^)_2_}_2_(μ^2^-η^3^-As_6_] (122) by the treatment of [(Cp^tt^)_2_Th(η^4^-C_4_H_6_)] with elemental As_4_ in boiling xylene,^145^Fig. 11. Complex 122 is also the first structurally characterised actinide complex containing metal–arsenic bonds, which is isostructural to the polyphosphorus analogue 77. In the molecular structure of 122, the Th–As bond distances span the range of 2.913(2) to 3.044(2) Å. However, it would be more than 20 years before more actinide–arsenic complexes started to emerge, reflecting the synthetic challenges of the area.

In 2015, Liddle, Scheer, and co-workers reported the diuranium complex [{U(Tren^TIPS^)}_2_(μ-η^2^:η^2^-HAsAsH)] (123)^146^ by the reaction of [U(Tren^TIPS^)(THF)][BPh_4_] with KAsH_2_ in 1 : 1.4 ratio *via* a dehydrocoupling process, Fig. 11. On one occasion, the uranium diarsenic complex [{U(Tren^TIPS^)}_2_(μ-η^2^:η^2^-As_2_)] (124)^146^ was also obtained from the reaction in less than 1% yield, which can be viewed as a fully dehydrocoupled product, Fig. 11. The solid-state structures of 123 and 124 are similar to each other, though the As–As bond distance in 123 (2.4102(13) Å) is longer than that in 124 (2.2568(14) Å), indicating As–As single and As=As double bond characters, respectively. The +4 oxidation state of 123 was confirmed by magnetometry data, so the diarsene ligand has been reduced to its diarsane-1,2-diide form. Complex 123 is the first example of HAsAsH complex for any d- or f-block metal. The characterisation data and theoretical calculations supported the presence of back-bonding-type interactions from uranium to the HAsAsH π*-orbital, indicating the strong π-accepting ability of this ligand. In other work, the authors also reported the structure of [{U(Tren^TIPS^)}_2_(μ-η^3^:η^3^-As_3_)] (125),^147^Fig. 11, but the low yield for this compound prevented further characterisation.

**Scheme 42:**
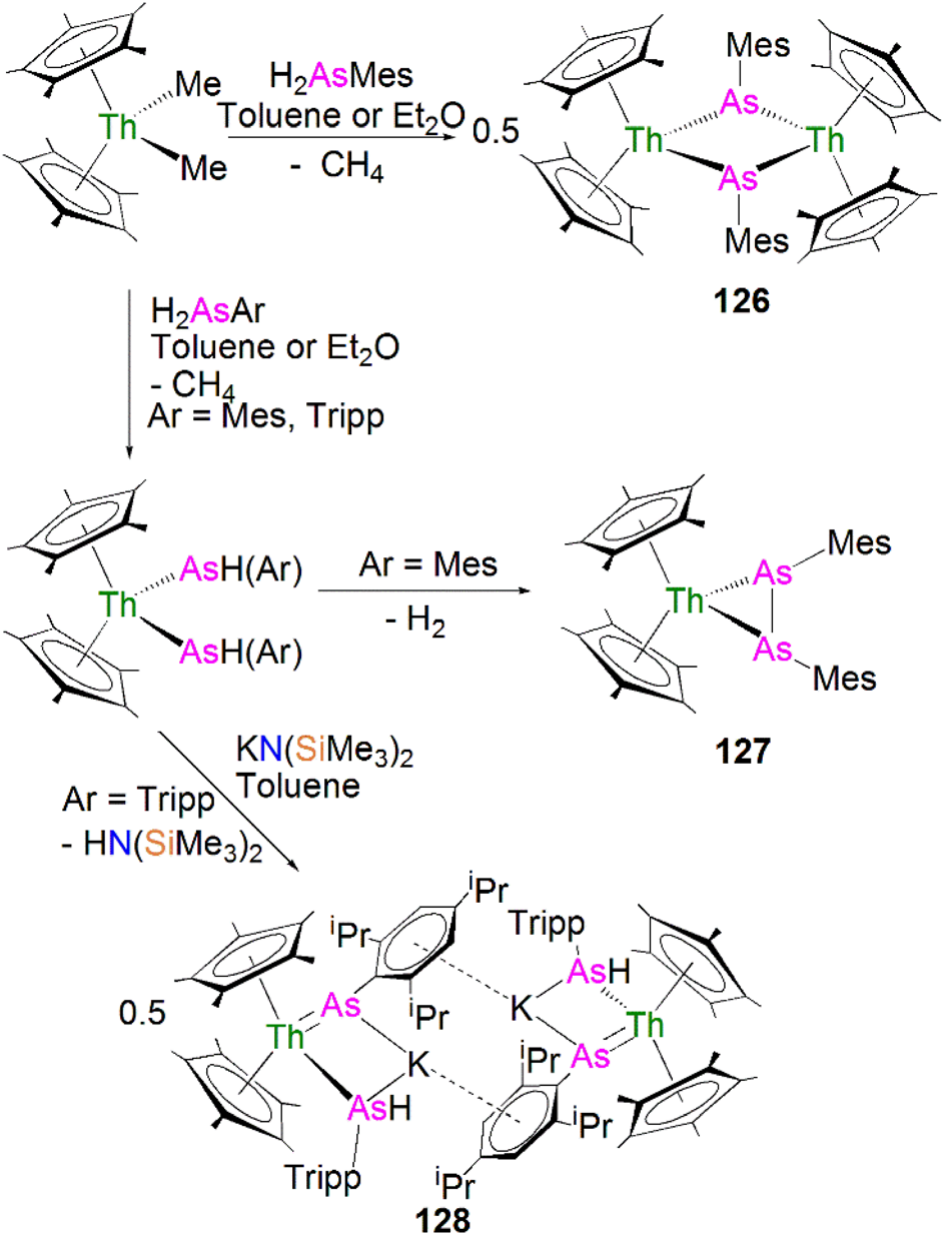
Synthesis of thorium arsinidiide and diarsene complexes 126–128.

In between 2016 and 2021, Walensky and co-workers reported the synthesis of thorium arsenic complexes, Scheme 42. Using methane elimination, the authors prepared the bridging dithorium arsinidiide complex [{Th(Cp*)_2_(μ-AsMes)_2_] (126) and the arsenide complexes [Th(Cp*)_2_(HAsAr)_2_] (Ar = Mes, Tripp) by the reactions of [Th(Cp*)_2_(Me)_2_] under different conditions.^148^ It was found that [Th(Cp*)_2_(HAsMes)_2_] is unstable at room temperature and converts to the dehydrocoupled product [Th(Cp*)_2_(μ-As_2_Mes_2_)] (127) with the release of H_2_ gas. Applying the same synthetic approach, the uranium analogues of 126 and 127 were prepared. With the bulkier substituents on the arsenide group, [Th(Cp*)_2_(HAsTripp)_2_] is thermally stable at room temperature and its reactivity with *tert*-butyl isocyanide ^*t*^BuNC to give an arsaazaallene product was investigated.^149^ The authors reported that deprotonation of [Th(Cp*)_2_{AsH(Tripp)}_2_] with one equivalent of KN(SiMe_3_)_2_ gave [Th(Cp*)_2_{μ-As(Tripp)}{μ-AsH(Tripp)}K]_2_ (128) in yields of 77%.^86^ The Th–As bond distances in 126 (2.8787(6) Å) and 127 (2.923(2)/2.971(3) Å) are quite long; by contrast, 128 exhibits a short Th–As distance of 2.7994(4) Å, indicating a multiple bonding interaction. This was probed computationally, revealing a Th=As Wiberg bond index (1.30) nearly twice that of the Th–As single bonds (0.70).

**Scheme 43:**
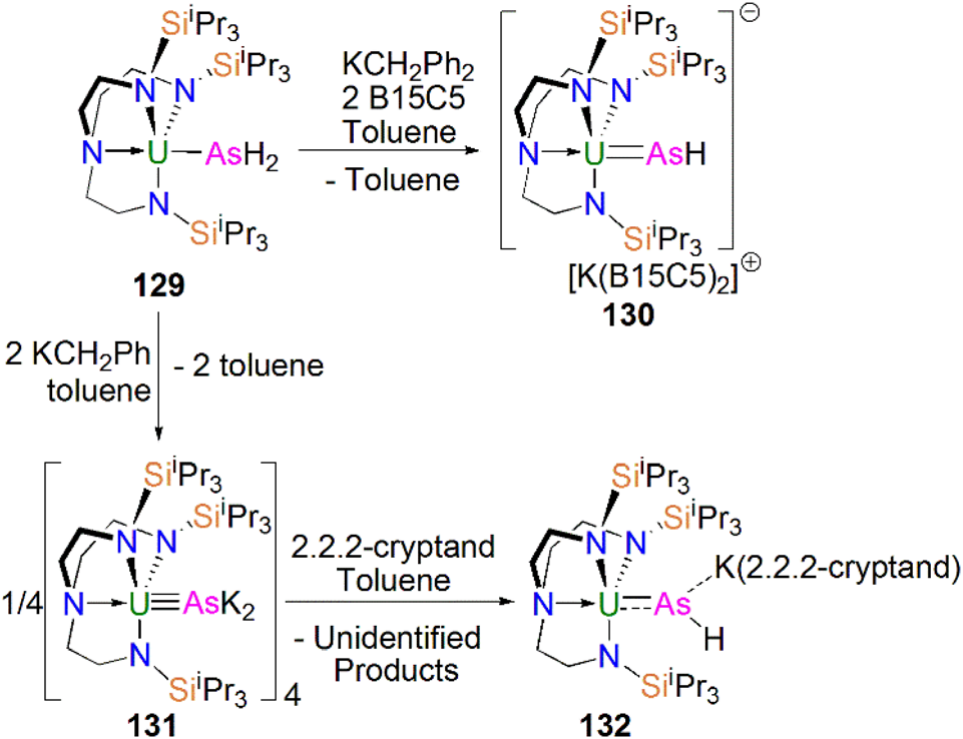
Synthesis of the uranium arsinidene complexes 130, arsenido 131, and arsinidiide 132 from the arsenide 129.

In 2015, Liddle, Scheer, and co-workers adapted the synthetic protocols used for the preparation of the uranium phosphinidene complex 64K. Deprotonation of 129 with benzyl potassium in the presence of two equivalents of B15C5 produced the analogous arsinidene complex [K(B15C5)_2_][U(Tren^TIPS^)(AsH)] (130),^147^Scheme 43. Most surprisingly, double deprotonation of 129 by two equivalents of benzyl potassium afforded the arsenido complex [{U(Tren^TIPS^)(μ-As)(μ-K_2_)}_4_] (131).^147^ Attempts to abstract the potassium cations from 131 using 2.2.2-cryptand resulted in formation of the arsinidiide [{U(Tren^TIPS^)(μ-AsH)}{K(2.2.2-cryptand)}] (132),^147^ with K/H exchange from solvent. The authors additionally reported an As–H stretch in the ATR-IR spectrum of 130 at 1857 cm^−1^. Complex 131 is a tetramer with an As_4_K_6_ adamantane-type core, with the bridging potassium ions self-evidently playing an indispensable role in stabilising the arsenido [As]^3−^ centres, *cf*. formation of 132.

The U–As distance of 2.7159(13) Å in the terminal arsinidene complex 130 is shorter than that seen for the potassium-capped arsinidiide complex 132 (2.7489(10) Å) and the arsenido complex 131 (2.730(2)–2.775(2) Å), which is due to the coordination of the potassium ion (or ions) to the arsenic centre(s). Computational analysis showed highly polarised single, double, and triple uranium arsenic bonding interactions for U–AsH_2_ (129), U=AsH (130) and U≡As (131), with substantial 5f orbital contributions to these bonds.

**Scheme 44:**
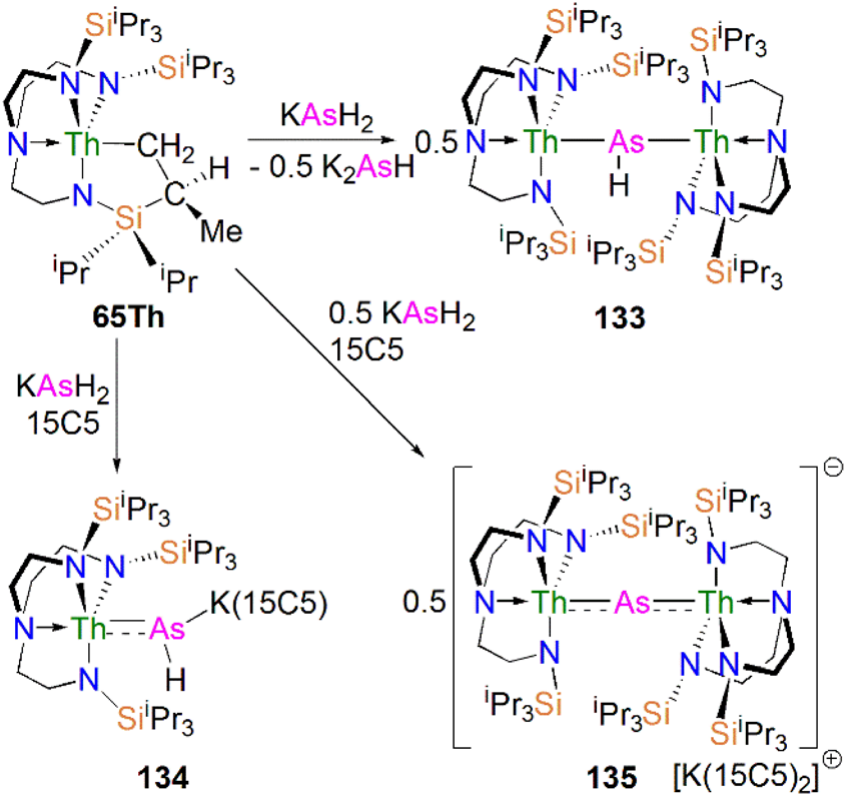
Synthesis of thorium arsinidiide and arsenido complexes 133–135.

In 2017, Liddle, Scheer, and co-workers adapted the protonolysis chemistry used to access 67, 70, and 71, performing protonolysis reactions of 65Th with different ratios of KAsH_2_ both with and without 15C5 to synthesise a range of Th–As complexes, Scheme 44, including the parent arsinidiide [{Th(Tren^TIPS^)}_2_(μ-AsH)] (133), the arsinidiide [{Th(Tren^TIPS^)(μ-AsH)}{K(15C5)}] (134), and the bridging arsenido [K(15C5)_2_][{Th(Tren^TIPS^)}_2_(μ-As)] (135), Scheme 44.^150^ The authors reported that attempts to prepare a terminal arsinidene [K(L)*_n_*][Th(Tren^TIPS^)(AsH)] (L = crown ethers or 2.2.2-cryptand) under various conditions were unsuccessful. Complex 134 could also be readily prepared in a 70% yield *via* the deprotonation of the parent arsenide [Th(Tren^TIPS^)AsH_2_] (the Th analogue of 129) with one equivalent of benzyl potassium in the presence of one equivalent of 15C5. The ATR-IR spectra of 133 and 134 exhibited As–H stretches of 1930 and 1922 cm^−1^, respectively. Consistent with the arsenido nature of 135, no As–H stretch was observed in the ATR-IR spectrum of 135.

Complexes 133–135 have respective Th–As distances of 2.9619(6)/3.0286(6), 2.8565(7) and 2.8063(14)/2.8060(14) Å, which are shorter than that of the parent arsenide [Th(Tren^TIPS^)AsH_2_] [3.065(3) Å]. When compared to the sum of the covalent single and double bond radii of Th and As of 2.96 and 2.57 Å, respectively, these Th–As bonds are relatively long and clearly polarised. An acute Th–As–H bond angle of 79.1(2)° in 134 suggested that a Th⋯H ‘agostic-type’ interaction could be present. The solid state structure of 135 is analogous to the thorium phosphido complex 70, with a symmetrical ThAsTh core and near linear Th–As–Th angle of 177.04(6)°. In line with the multiple bonding interaction in the ThAsTh linkage, the Th–As distances exhibited by 135 (2.8063(14)/2.8060(14) Å) are shorter than those in 133 (2.9619(6)/3.0286(6) Å). The authors reported that attempts to prepare a terminal Tren^TIPS^ thorium arsenido Th≡As species using the similar double deprotonation method for 132 proved unsuccessful; indeed, closely related work attempting to construct thorium nitrides supported by Tren^TIPS^ consistently resulted in the formation of parent imido complexes.

**Scheme 45:**
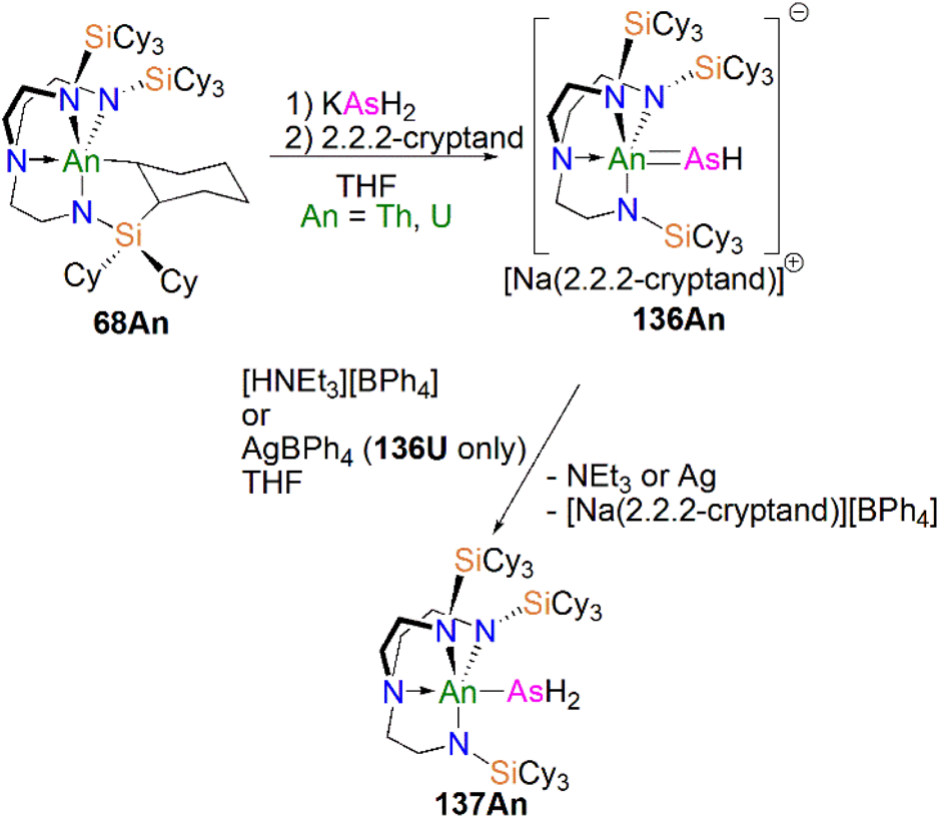
Synthesis of terminal actinide parent arsinidene and arsenide complexes 136An and 137An.

In 2022, Liddle, Scheer, and co-workers showed that a terminal parent arsinidene at thorium (and uranium for comparison) could be isolated using the bulky Tren^TCHS^ ligand, Scheme 45. Specifically, reaction of 68An with KAsH_2_ in the presence of 2.2.2-cryptand in THF afforded directly, in good yields, [K(2.2.2-cryptand)][An(Tren^TIPS^)(AsH)] (136An), Scheme 45.^84^ The molecular structures of 136An confirmed the presence of terminal arsinidene (AsH)^2−^ units that are well-protected by the tricyclohexylsilyl groups. The An–As bond distances of 2.8521(8) Å and 2.7581(6) Å for 136Th and 136U, respectively, are statistically invariant to those in 134 and 130, indicating multiple bonding interactions in these An = AsH linkages that were confirmed by DFT calculations. ‘Agostic-type’ interactions between the metal ions and arsinidene ligands are observed in both structures, with An–As–H angles of 60.50(12)° and 61.18(12)°, respectively. In addition, ATR-IR spectroscopy revealed As–H stretches at 1867 and 1875 cm^−1^ for 136Th and 136U, respectively. The authors found that treatment of 136An with [HNEt_3_][BPh_4_] in THF results in the isolation of the parent arsenide complexes [An(Tren^TCHS^)(AsH_2_)] (137An) in good yields, Scheme 45; oxidation of 136U also gave the protonated product 137U.^84^

### 7.2 Actinide 2-arsaethynolate complexes

Following the aforementioned developments in f-element 2-phosphethynolate chemistry, the chemistry of the corresponding 2-arsaethynolate anion (OCAs)^−^ is beginning to emerge. In 2018, Meyer and co-workers reported reactions of the U(iii) complex [U{(^Ad,Me^ArO)_3_N}(DME)] ({(^Ad,Me^ArO)_3_N}^3−^ = trianion of tris(2-hydroxy-3-(1-adamantyl)-5-methylbenzyl)amine) with one equivalent of [Na(OCAs)(dioxane)_3_], and 2.2.2-cryptand giving [Na(2.2.2-cryptand)][{U((^Ad,Me^ArO)_3_N)(THF)}(μ-O){U((^Ad,Me^ArO)_3_N)(CAs)}].^151^ In contrast, using two equivalents of [Na(OCAs)(dioxane)_3_] yielded the binuclear, μ-oxo bridged diuranium(iv/iv) complex [Na(2.2.2-cryptand)]_2_[{U((^Ad,Me^ArO)_3_N)}_2_(μ-O)(μ-AsCAs)], which contains a μ:η^1^-η^1^-coordinated (AsCAs)^2−^ ligand.^151^ In 2019, Liddle, Scheer, and co-workers reported the first U–OCAs complex [U(Tren^TIPS^)(OCAs)], and treatment of this complex with KC_8_ and 2.2.2-cryptand as an *in situ* electride mixture yielded [K(2.2.2-cryptand)][{U(Tren^TIPS^)}_2_{μ-η^2^(OAs):η^2^(CAs)–OCAs}] which is the As analogue of 75, with a highly reduced bent, carbene-like OCAs-ligand.^152^ In contrast, reduction or photolysis of [U(Tren^TIPS^)(OCAs)] with [U(Tren^TIPS^)] gave the mixed-valence arsenido complex [{U(Tren^TIPS^)}_2_(μ-As)], in very low yield, or 123, respectively.^152^ All of these results demonstrate the challenges of using the OCAs ligand to synthesise actinide–arsenic bonds and also that the synthetic methods and ancillary ligands drive the (OCAs)^−^ bond cleavage chemistry in very different directions.

### 7.3 Actinide antimony complexes

**Scheme 46:**
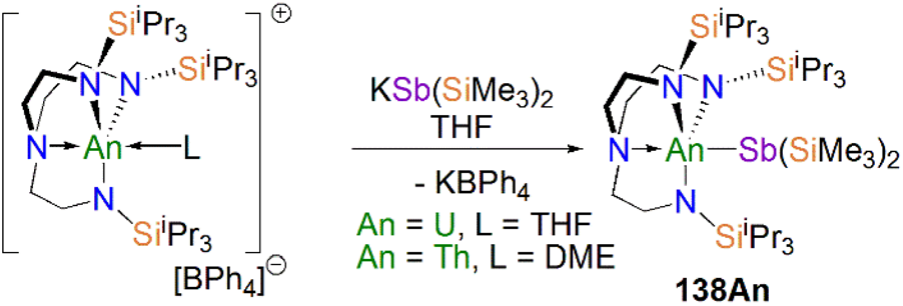
Synthesis of the actinide–antimony complexes 138An.

In 2017, Liddle, Scheer, and co-workers reported the isolation and characterisation of the first discrete (*i.e.* not multi-centre) An–Sb bonds (An=U and Th) from the reactions of [An(Tren^TIPS^)(L)][BPh_4_] (An=U, L=THF; An=Th, L=DME) and KSb(SiMe_3_)_2_ in THF, yielding [An(Tren^TIPS^){Sb(SiMe_3_)_2_}] (138An), Scheme 46.^153^ The closely related uranium complex [U(Tren^DMBS^){Sb(SiMe_3_)_2_}] with a less sterically bulky supporting ligand could also be prepared using the same synthetic approach.^153^

Complexes 138Th and 138U exhibit An–Sb distances of 3.2849(3) and 3.2089(6) Å, respectively. The authors observed a shorter U–Sb bond in 138U (3.2089(6) Å) compared to [U(Tren^DMBS^){Sb(SiMe_3_)_2_}] (3.2437(8) Å), likely a result of the differing sterics between the Tren^TIPS^ and Tren^DMBS^ ligand systems; the latter results in more orthogonal, and presumably weaker, binding of the stibide ligand. The analogous U–P and U–As complexes were also prepared in that study, revealing increasingly pyramidalised pnictide centres as the group is descended.

### 7.4 Actinide bismuth complexes

**Scheme 47:**
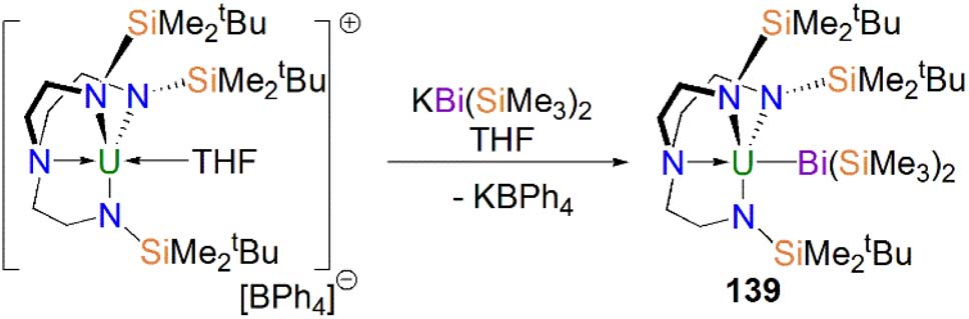
Synthesis of the uranium–bismuthide complex 139.

In the same publication describing the work in Section 7.3, Liddle, Scheer, and co-workers also reported the synthesis and characterisation of the first two-centre-two-electron (2c–2e) U–Bi bond, [U(Tren^DMBS^){Bi(SiMe_3_)_2_}] (139), by the reaction of [U(Tren^DMBS^)(THF)][BPh_4_] with KBi(SiMe_3_)_2_ in THF, Scheme 47.^153^ It was found that the U–Bi bond could not be stabilised using the bulkier Tren^TIPS^ ligands, likely due to steric overload. Complex 139 exhibits U–Bi distances of 3.3208(4) Å which is longer than the above An–Sb bonds. Whilst the U–Bi bond in 139 was isolable, no Th–Bi bond could be isolated with Tren^TIPS^ or Tren^DMBS^, underscoring the fragility of these linkages. Complex 139 is the only monomeric actinide complex containing a discrete bond to Bi to date.

**Scheme 48:**
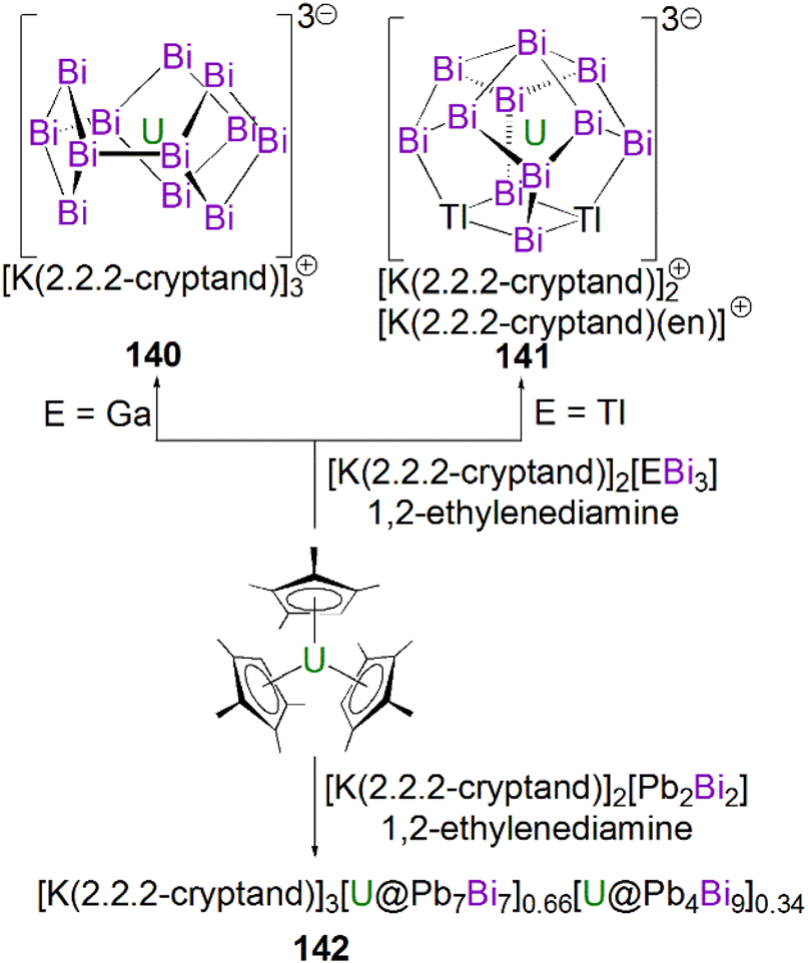
Synthesis of the uranium–bismuth Zintl anionic clusters 140–142. U–Bi bonds are omitted for clarity.

Dehnen and co-workers reported actinide bismuth clusters in 2016. Reaction of [U(C_5_Me_4_H)_3_] with [K(2.2.2-cryptand)]_2_[EE′Bi_2_]·en (E = Ga, Tl, E′ = Bi; E = E′ = Pb) in 1,2-ethlyenediamine gave either [K(2.2.2-cryptand)]_3_[U@Bi_12_] (140), [K(2.2.2-cryptand)]_2_[K(2.2.2-cryptand)(en)][U@Tl_2_Bi_11_] (141) or [K(2.2.2-cryptand)]_3_[U@Pb_7_Bi_7_]_0.66_[U@Pb_4_Bi_9_]_0.34_ (142), Scheme 48.^154^ The structure of the trianionic fragment of 140 is analogous to the antimony cluster 101Ln, exhibiting longer equatorial U–Bi bond distances (3.463(3)–3.545(3) Å) than the non-equatorial U–Bi bond distances (3.119(3)–3.167(3) Å). Complex 141 exhibits a 13-vertex cluster that is analogous to 114Ln, with U–Bi distances ranging from 3.068(1) to 3.4515(6) Å. Complex 142 has an isomorphic structure to 118Ln/120Ln, exhibiting U–Bi bond distances ranging from 2.885(9) to 3.6885(12) Å.

**Scheme 49:**
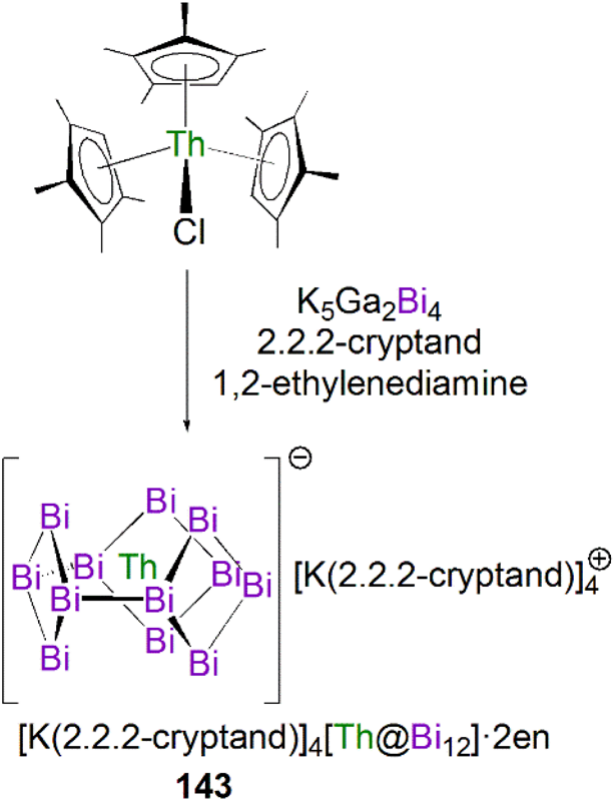
Synthesis of the thorium–bismuth Zintl anionic clusters 143. The Th–Bi bonds are omitted for clarity.

In 2021, Dehnen and co-workers extended this Bi cluster chemistry to include the first example of a thorium bismuth cluster containing Th–Bi bonds, Scheme 49. The authors reported that reaction of [(C_5_Me_4_H)_3_Th(Cl)] with K_5_Ga_2_Bi_4_, which can be used as an *in situ* source of [GaBi_3_]^2−^ and Bi_4_^2−^, in the presence of 2.2.2-cryptand in 1,2-ethlyenediamine afforded [K(2.2.2-cryptand)]_4_[Th@Bi_12_]·2en (143) as black, prismatic crystals, Scheme 49.^155^ The structure of 143 was confirmed by single-crystal X-ray diffraction and the Th : Bi ratio within the cluster was verified by micro-X-ray fluorescence spectroscopy. The molecular structure revealed Bi–Bi bond distances over a relatively small range (3.0420(14)–3.132(1) Å) and Th–Bi bond lengths (3.2104(11)–3.5908(9) Å) that are comparable to the An–Bi bonds in 139–142. Magnetic data and theoretical studies on 143 confirm the formal assignment as Th^4+^ and Bi_12_^8−^ with a remarkable ring current strength of 24.8 nAT^−1^ for [Th@Bi_12_]^4−^ (and 23.7 nAT^−1^ for Bi_12_^8−^). This is much larger than in 6π-aromatic benzene (11.4 nAT^−1^), but close to that in 26π-aromatic porphine (25.3 nAT^−1^), despite the much smaller number of 2π-electrons involved. The aromatic nature of [Th@Bi12]^4−^ extends aromaticity to the heaviest all-metal inorganic system.

## 8. Conclusions and outlook

Although f-element heavy pnictogen chemistry initially progressed quite slowly for many decades, with the resurgence of non-aqueous f-element chemistry momentum in this area has increased significantly in recent years, as evidenced by the burgeoning array of novel metal-heavy-pnictogen bond types that are now known. These well-characterised compounds have enabled us to secure new structural motifs and probe the nature of f-element ligand chemical bonds to further deepen our understanding of chemical bonding with increasingly heavy ions in non-relativistic to relativistic regimes. Overall, f-element heavier pnictogen chemistry is developing well but there are certainly numerous opportunities to advance knowledge and understanding in this burgeoning field.

Looking forward, there are several appealing directions for researchers to explore in this area: (1) in general terms, f-element-phosphorus chemistry is maturing, but arsenic and especially antimony and bismuth are poorly developed – bringing the latter three to the same level of maturity as phosphorus will do much to elucidate periodic trends; (2) there are still relatively few actinide–pnictidene/ido multiple bond complexes and even fewer lanthanide analogues – however, the reports of isolated complexes to date suggests that there is ample scope to secure new Ln and An double and triple bonds to P, As, Sb, and Bi if the right supporting ligands can be identified and coupled with suitable synthetic approaches; (3) heavy analogues of dinitrogen are now known and even as radical species, but examples remain few in number – developing better synthetic approaches would open the area up and provide interesting electronic and physicochemical properties and potential atom-transfer methodologies; and, (4) though relatively few in number, it is already clear that f-element pnictogen clusters can exhibit novel magnetic and aromaticity properties – expanding the range of such compounds can only enhance our understanding of these fundamental phenomena.

## Author contributions

J. D., D. P. M. and S. T. L. designed the structure of the review. All authors contributed to the writing of the article.

## Conflicts of interest

There are no conflicts to declare.

## Supplementary Material

## References

[cit1] Liu J. P. , Willard M., Tang W., Brück E., de Boer F., Liu E., Liu J., Felser C., Fecher G., Wollmann L., Isnard O., Burzo E., Liu S., Herbst J. F., Hu F., Liu Y., Sun J., Shen B. and de Visser A., Metallic magnetic materials, in Handbook of Magnetism and Magnetic Materials, ed. Coey M. and Parkin S., Springer, Cham, 2021

[cit2] Gutfleisch O., Willard M. A., Brück E., Chen C. H., Sankar S. G., Liu J. P. (2011). Adv. Mater..

[cit3] Hasegawa Y. and Kitagawa Y., Lanthanide-based materials for electroluminescence, in Modern Applications of Lanthanide Luminescence, ed. de Bettencourt-Dias A., Springer, Cham, 2021, Springer Series on Fluorescence, vol. 19

[cit4] Marin R., Jaque D. (2021). Chem. Rev..

[cit5] Dong H., Du S.-R., Zheng X.-Y., Lyu G.-M., Sun L.-D., Li L.-D., Zhang P.-Z., Zhang C., Yan C.-H. (2015). Chem. Rev..

[cit6] Hyre A. S., Doerrer L. H. (2020). Coord. Chem. Rev..

[cit7] Molander G. A. (1992). Chem. Rev..

[cit8] Ortu F. (2022). Chem. Rev..

[cit9] Scharfe M., Lira-Parada P. A., Amrute A. P., Mitchell S., Pérez-Ramírez J. (2016). J. Catal..

[cit10] Zhang Z., Liu S., Li X., Qin T., Wang L., Bo X., Liu Y., Xu L., Wang S., Sun X., Lu Y., Luo F., Liu S. (2018). ACS Appl. Mater. Interfaces.

[cit11] McAdams S. G., Ariciu A. M., Kostopoulos A. K., Walsh J. P. S., Tuna F. (2017). Coord. Chem. Rev..

[cit12] Cheng X., Zhou J., Yue J., Wei Y., Gao C., Xie X., Huang L. (2022). Chem. Rev..

[cit13] Leoncini A., Huskens J., Verboom W. (2017). Chem. Soc. Rev..

[cit14] Gelis A. V., Kozak P., Breshears A. T., Brown M. A., Launiere C., Campbell E. L., Hall G. B., Levitskaia T. G., Holfeltz V. E., Lumetta G. J. (2019). Sci. Rep..

[cit15] Atwood D. A. , The Rare Earth Elements: Fundamentals and Applications, John Wiley & Sons, Ltd, Chichester, UK, 2012

[cit16] Liddle S. T. (2015). Angew. Chem., Int. Ed..

[cit17] Bessen N. P., Jackson J. A., Jensen M. P., Shafer J. C. (2020). Coord. Chem. Rev..

[cit18] Schädle D., Anwander R. (2019). Chem. Soc. Rev..

[cit19] Keener M., Maria L., Mazzanti M. (2023). Chem. Sci..

[cit20] Vilanova S. P. and Walensky J. R., Encyclopedia of Inorganic and Bioinorganic Chemistry, John Wiley & Sons, Ltd, 2018

[cit21] Groom C. R., Bruno I. J., Lightfoot M. P., Ward S. C. (2016). Acta Crystallogr., Sect. B: Struct. Sci., Cryst. Eng. Mater..

[cit22] Ye L.-W., Zhu X.-Q., Sahani R. L., Xu Y., Qian P.-C., Liu R.-S. (2021). Chem. Rev..

[cit23] Zhu C., Xia H. (2018). Acc. Chem. Res..

[cit24] Aktaş H., Slootweg J. C., Lammertsma K. (2010). Angew. Chem., Int. Ed..

[cit25] Summerscales O. T., Gordon J. C. (2013). RSC Adv..

[cit26] Li T., Kaercher S., Roesky P. W. (2014). Chem. Soc. Rev..

[cit27] García M. E., García-Vivó D., Ramos A., Ruiz M. A. (2017). Coord. Chem. Rev..

[cit28] Qiao L., Zhang C., Zhang X.-W., Wang Z.-C., Yin H., Sun Z.-M. (2020). Chin. J. Chem..

[cit29] Giusti L., Landaeta V. R., Vanni M., Kelly J. A., Wolf R., Caporali M. (2021). Coord. Chem. Rev..

[cit30] Mills D. P., Evans P. (2021). Chem.–Eur. J..

[cit31] Grant L. N., Mindiola D. J. (2019). Chem.–Eur. J..

[cit32] Fryzuk M. D., Haddad T. S., Berg D. J. (1990). Coord. Chem. Rev..

[cit33] Ramirez B., Sharma P., Eisenhart R. J., Gagliardi L., Lu C. C. (2019). Chem. Sci..

[cit34] Ramirez B., Lu C. C. (2020). J. Am. Chem. Soc..

[cit35] Izod K., O'Shaughnessy P., Sheffield J. M., Clegg W., Liddle S. T. (2000). Inorg. Chem..

[cit36] Izod K., Liddle S. T., McFarlane W., Clegg W. (2004). Organometallics.

[cit37] Izod K., Liddle S. T., Clegg W. (2004). Chem. Commun..

[cit38] Rabe G. W., Riede J., Schier A. (1995). J. Chem. Soc., Chem. Commun..

[cit39] Rabe G. W., Ziller J. W. (1995). Inorg. Chem..

[cit40] Rabe G. W., Riede J., Schier A. (1996). Organometallics.

[cit41] Atlan S., Nief F., Ricard L. (1995). Bull. Soc. Chim. Fr..

[cit42] Nief F., Ricard L. (1997). J. Organomet. Chem..

[cit43] Rabe G. W., Guzei I. A., Rheingold A. L. (1997). Inorg. Chem..

[cit44] Westerhausen M., Schneiderbauer S., Hartmann M., Warchhold M., Nöth H. (2002). Z. Anorg. Allg. Chem..

[cit45] Westerhausen M., Schneiderbauer S., Makropoulos N., Warchhold M., Nöth H., Piotrowski H., Karaghiosoff K. (2002). Organometallics.

[cit46] Arnold P. L., Cloke F. G. N., Hitchcock P. B. (1997). Chem. Commun..

[cit47] Jacquot L., Xémard M., Clavaguéra C., Nocton G. (2014). Organometallics.

[cit48] Jaoul A., Clavaguéra C., Nocton G. (2016). New J. Chem..

[cit49] Arliguie T., Doux M., Mézailles N., Thuéry P., Le Floch P., Ephritikhine M. (2006). Inorg. Chem..

[cit50] Masuda J. D., Jantunen K. C., Ozerov O. V., Noonan K. J. T., Gates D. P., Scott B. L., Kiplinger J. L. (2008). J. Am. Chem. Soc..

[cit51] Cui P., Chen Y., Xu X., Sun J. (2008). Chem. Commun..

[cit52] Cui P., Chen Y., Borzov M. V. (2010). Dalton Trans..

[cit53] Wicker B. F., Scott J., Andino J. G., Gao X., Park H., Pink M., Mindiola D. J. (2010). J. Am. Chem. Soc..

[cit54] Lv Y., Kefalidis C. E., Zhou J., Maron L., Leng X., Chen Y. (2013). J. Am. Chem. Soc..

[cit55] Zhou J., Li T., Maron L., Leng X., Chen Y. (2015). Organometallics.

[cit56] Wang K., Luo G., Hong J., Zhou X., Weng L., Luo Y., Zhang L. (2014). Angew. Chem., Int. Ed..

[cit57] Tian H. , Hong J., Wang K., Rosal I. de., Maron L., Zhou X. and Zhang L., CSD Communications, Private Communications, 2017

[cit58] Pugh T., Tuna F., Ungur L., Collison D., McInnes E. J. L., Chibotaru L. F., Layfield R. A. (2015). Nat. Commun..

[cit59] Feng B., Xiang L., McCabe K. N., Maron L., Leng X., Chen Y. (2020). Nat. Commun..

[cit60] Feng B., Xiang L., Carpentier A., Maron L., Leng X., Chen Y. (2021). J. Am. Chem. Soc..

[cit61] Rieser T. E., Wetzel P., Maichle-Mössmer C., Sirsch P., Anwander R. (2023). J. Am. Chem. Soc..

[cit62] Lv Y., Xu X., Chen Y., Leng X., Borzov M. V. (2011). Angew. Chem., Int. Ed..

[cit63] Konchenko S. N., Pushkarevsky N. A., Gamer M. T., Köppe R., Schnöckel H., Roesky P. W. (2009). J. Am. Chem. Soc..

[cit64] Huang W., Diaconescu P. L. (2012). Chem. Commun..

[cit65] Li T., Wiecko J., Pushkarevsky N. A., Gamer M. T., Koeppe R., Konchenko S. N., Scheer M., Roesky P. W. (2011). Angew. Chem., Int. Ed..

[cit66] Li T., Gamer M. T., Scheer M., Konchenko S. N., Roesky P. W. (2013). Chem. Commun..

[cit67] Schoo C., Bestgen S., Köppe R., Konchenko S. N., Roesky P. W. (2018). Chem. Commun..

[cit68] Zhang F., Zhang J., Chen Z., Weng L., Zhou X. (2019). Inorg. Chem..

[cit69] Hauser A., Münzfeld L., Schlittenhardt S., Köppe R., Uhlmann C., Rauska U.-C., Ruben M., Roesky P. W. (2023). Chem. Sci..

[cit70] Edwards P. G., Andersen R. A., Zalkin A. (1984). Organometallics.

[cit71] Newell B. S., Schwaab T. C., Shores M. P. (2011). Inorg. Chem..

[cit72] Napoline J. W., Kraft S. J., Matson E. M., Fanwick P. E., Bart S. C., Thomas C. M. (2013). Inorg. Chem..

[cit73] Ward A. L., Lukens W. W., Lu C. C., Arnold J. (2014). J. Am. Chem. Soc..

[cit74] Ayres A. J., Zegke M., Ostrowski J. P. A., Tuna F., McInnes E. J. L., Wooles A. J., Liddle S. T. (2018). Chem. Commun..

[cit75] Ayres A. J., Wooles A. J., Zegke M., Tuna F., Liddle S. T. (2019). Inorg. Chem..

[cit76] Perales D., Bhowmick R., Zeller M., Miro P., Vlaisavljevich B., Bart S. C. (2022). Chem. Commun..

[cit77] Garner M. E., Arnold J. (2017). Organometallics.

[cit78] Garner M. E., Parker B. F., Hohloch S., Bergman R. G., Arnold J. (2017). J. Am. Chem. Soc..

[cit79] Ritchey J. M., Zozulin A. J., Wrobleski D. A., Ryan R. R., Wasserman H. J., Moody D. C., Paine R. T. (1985). J. Am. Chem. Soc..

[cit80] Hay P. J., Ryan R. R., Salazar K. V., Wrobleski D. A., Sattelberger A. P. (1986). J. Am. Chem. Soc..

[cit81] Behrle A. C., Castro L., Maron L., Walensky J. R. (2015). J. Am. Chem. Soc..

[cit82] Gardner B. M., Balázs G., Scheer M., Tuna F., McInnes E. J. L., McMaster J., Lewis W., Blake A. J., Liddle S. T. (2014). Angew. Chem., Int. Ed..

[cit83] Wildman E. P., Balázs G., Wooles A. J., Scheer M., Liddle S. T. (2016). Nat. Commun..

[cit84] Du J., Balázs G., Seed J. A., Cryer J. D., Wooles A. J., Scheer M., Liddle S. T. (2022). Angew. Chem., Int. Ed..

[cit85] Duttera M. R., Day V. W., Marks T. J. (1984). J. Am. Chem. Soc..

[cit86] Rookes T. M., Gardner B. M., Balázs G., Gregson M., Tuna F., Wooles A. J., Scheer M., Liddle S. T. (2017). Angew. Chem., Int. Ed..

[cit87] Vilanova S. P., Alayoglu P., Heidarian M., Huang P., Walensky J. R. (2017). Chem.–Eur. J..

[cit88] Rungthanaphatsophon P., Duignan T. J., Myers A. J., Vilanova S. P., Barnes C. L., Autschbach J., Batista E. R., Yang P., Walensky J. R. (2018). Inorg. Chem..

[cit89] Tarlton M. L., Yang Y., Kelley S. P., Maron L., Walensky J. R. (2021). Organometallics.

[cit90] Tarlton M. L., Yu X., Ward R. J., Kelley S. P., Autschbach J., Walensky J. R. (2021). Chem.–Eur. J..

[cit91] Tarlton M. L., Vilanova S. P., Kaumini M. G., Kelley S. P., Huang P., Walensky J. R. (2021). Inorg. Chem..

[cit92] Tarlton M. L., Yang Y., Kelley S. P., Maron L., Walensky J. R. (2021). Organometallics.

[cit93] Zhang C., Hou G., Zi G., Ding W., Walter M. D. (2019). Inorg. Chem..

[cit94] CSD Search by 10-08-2023

[cit95] Arney D. S. J., Schnabel R. C., Scott B. C., Burns C. J. (1996). J. Am. Chem. Soc..

[cit96] Hall S. W., Huffman J. C., Miller M. M., Avens L. R., Burns C. J., Sattelberger A. P., Arney D. S. J., England A. F. (1993). Organometallics.

[cit97] Zhang C., Hou G., Zi G., Ding W., Walter M. D. (2018). J. Am. Chem. Soc..

[cit98] Zhang C., Hou G., Zi G., Walter M. D. (2019). Dalton Trans..

[cit99] Wang Y., Zhang C., Zi G., Ding W., Walter M. D. (2019). New J. Chem..

[cit100] Wang D., Ding W., Hou G., Zi G., Walter M. D. (2020). Chem.–Eur. J..

[cit101] Wang D., Wang S., Hou G., Zi G., Walter M. D. (2020). Inorg. Chem..

[cit102] Wang D., Hou G., Zi G., Walter M. D. (2020). Organometallics.

[cit103] Wang D., Hou G., Zi G., Walter M. D. (2021). Organometallics.

[cit104] Wang S., Li T., Heng Y., Hou G., Zi G., Walter M. D. (2021). Organometallics.

[cit105] Wang D., Wang S., Li T., Heng Y., Hou G., Zi G., Walter M. D. (2021). Dalton Trans..

[cit106] King D. M., Tuna F., McInnes E. J. L., McMaster J., Lewis W., Blake A. J., Liddle S. T. (2012). Science.

[cit107] King D. M., Tuna F., McInnes E. J. L., McMaster J., Lewis W., Blake A. J., Liddle S. T. (2013). Nat. Chem..

[cit108] King D. M., McMaster J., Tuna F., McInnes E. J. L., Lewis W., Blake A. J., Liddle S. T. (2014). J. Am. Chem. Soc..

[cit109] Magnall R., Balázs G., Lu E., Tuna F., Wooles A. J., Scheer M., Liddle S. T. (2019). Angew. Chem., Int. Ed..

[cit110] Du J., Balázs G., Wooles A. J., Scheer M., Liddle S. T. (2021). Angew. Chem., Int. Ed..

[cit111] Scherer O. J., Werner B., Heckmann G., Wolmershäuser G. (1991). Angew. Chem., Int. Ed. Engl..

[cit112] Frey A. S. P., Cloke F. G. N., Hitchcock P. B., Green J. C. (2011). New J. Chem..

[cit113] Formanuik A., Ortu F., Beekmeyer R., Kerridge A., Adams R. W., Mills D. P. (2016). Dalton Trans..

[cit114] Patel D., Tuna F., McInnes E. J. L., Lewis W., Blake A. J., Liddle S. T. (2013). Angew. Chem., Int. Ed..

[cit115] Gardner B. M., Tuna F., McInnes E. J. L., McMaster J., Lewis W., Blake A. J., Liddle S. T. (2015). Angew. Chem., Int. Ed..

[cit116] Fang W., Douair I., Hauser A., Li K., Zhao Y., Roesky P. W., Wang S., Maron L., Zhu C. (2022). CCS Chem..

[cit117] Du J., Hunger D., Seed J. A., Cryer J. D., King D. M., Wooles A. J., van Slageren J., Liddle S. T. (2021). J. Am. Chem. Soc..

[cit118] Evans W. J., Ulibarri T. A., Ziller J. W. (1988). J. Am. Chem. Soc..

[cit119] Schumann H., Palamidis E., Loebel J., Plckardt J. (1988). Organometallics.

[cit120] Evans W. J., Leman J. T., Ziller J. W., Khan S. I. (1996). Inorg. Chem..

[cit121] Nief F., Turcitu D., Ricard L. (2002). Chem. Commun..

[cit122] Pugh T., Kerridge A., Layfield R. A. (2015). Angew. Chem., Int. Ed..

[cit123] Pugh T., Vieru V., Chibotaru L. F., Layfield R. A. (2016). Chem. Sci..

[cit124] Arleth N., Gamer M. T., Köppe R., Konchenko S. N., Fleischmann M., Scheer M., Roesky P. W. (2016). Angew. Chem., Int. Ed..

[cit125] Reinfandt N., Michenfelder N., Schoo C., Yadav R., Reichl S., Konchenko S. N., Unterreiner A. N., Scheer M., Roesky P. W. (2021). Chem.–Eur. J..

[cit126] Schoo C., Köppe R., Piesch M., Gamer M. T., Konchenko S. N., Scheer M., Roesky P. W. (2018). Chem.–Eur. J..

[cit127] Schöttle C., Bockstaller P., Popescu R., Gerthsen D., Feldmann C. (2015). Angew. Chem., Int. Ed..

[cit128] Schoo C., Bestgen S., Egeberg A., Seibert J., Konchenko S. N., Feldmann C., Roesky P. W. (2019). Angew. Chem., Int. Ed..

[cit129] Reinfandt N., Hauser A., Münzfeld L., Roesky P. W. (2022). Chem. Sci..

[cit130] Balázs G., Sierka M., Scheer M. (2005). Angew. Chem., Int. Ed..

[cit131] Evans W. J., Gonzales S. L., Ziller J. W. (1992). J. Chem. Soc., Chem. Commun..

[cit132] Min X., Popov I. A., Pan F.-X., Li L.-J., Matito E., Sun Z.-M., Wang L.-S., Boldyrev A. I. (2016). Angew. Chem., Int. Ed..

[cit133] Pugh T., Chilton N. F., Layfield R. A. (2017). Chem. Sci..

[cit134] Schoo C., Bestgen S., Egeberg A., Klementyeva S., Feldmann C., Konchenko S. N., Roesky P. W. (2018). Angew. Chem., Int. Ed..

[cit135] Reinfandt N., Schoo C., Dütsch L., Köppe R., Konchenko S. N., Scheer M., Roesky P. W. (2021). Chem.–Eur. J..

[cit136] Evans W. J., Gonzales S. L., Ziller J. W. (1991). J. Am. Chem. Soc..

[cit137] Chung A. B., Ryan A. J., Fang M., Ziller J. W., Evans W. J. (2021). Inorg. Chem..

[cit138] Zhang P., Nabi R., Staab J. K., Chilton N. F., Demir S. (2023). J. Am. Chem. Soc..

[cit139] Lips F., Clérac R., Dehnen S. (2011). Angew. Chem., Int. Ed..

[cit140] Lips F., Hołyńska M., Clérac R., Linne U., Schellenberg I., Pöttgen R., Weigend F., Dehnen S. (2012). J. Am. Chem. Soc..

[cit141] Weinert B., Weigend F., Dehnen S. (2012). Chem.–Eur. J..

[cit142] Weinert B., Müller F., Harms K., Clérac R., Dehnen S. (2014). Angew. Chem., Int. Ed..

[cit143] Ababei R., Massa W., Weinert B., Pollak P., Xie X., Clérac R., Weigend F., Dehnen S. (2015). Chem.–Eur. J..

[cit144] Zhang P., Benner F., Chilton N. F., Demir S. (2022). Chem.

[cit145] Scherer O. J., Schulze J., Wolmershäuser G. (1994). J. Organomet. Chem..

[cit146] Gardner B. M., Balázs G., Scheer M., Wooles A. J., Tuna F., McInnes E. J. L., McMaster J., Lewis W., Blake A. J., Liddle S. T. (2015). Angew. Chem., Int. Ed..

[cit147] Gardner B. M., Balázs G., Scheer M., Tuna F., McInnes E. J. L., McMaster J., Lewis W., Blake A. J., Liddle S. T. (2015). Nat. Chem..

[cit148] Tarlton M. L., Fajen O. J., Kelley S. P., Kerridge A., Malcomson T., Morrison T. L., Shores M. P., Xhani X., Walensky J. R. (2021). Inorg. Chem..

[cit149] Behrle A. C., Walensky J. R. (2016). Dalton Trans..

[cit150] Wildman E. P., Balázs G., Wooles A. J., Scheer M., Liddle S. T. (2017). Nat. Commun..

[cit151] Hoerger C. J., Heinemann F. W., Louyriac E., Rigo M., Maron L., Grützmacher H., Driess M., Meyer K. (2019). Angew. Chem., Int. Ed..

[cit152] Magnall R., Balázs G., Lu E., Kern M., van Slageren J., Tuna F., Wooles A. J., Scheer M., Liddle S. T. (2019). Chem.–Eur. J..

[cit153] Rookes T. M., Wildman E. P., Balázs G., Gardner B. M., Wooles A. J., Gregson M., Tuna F., Scheer M., Liddle S. T. (2018). Angew. Chem., Int. Ed..

[cit154] Lichtenberger N., Wilson R. J., Eulenstein A. R., Massa W., Clérac R., Weigend F., Dehnen S. (2016). J. Am. Chem. Soc..

[cit155] Eulenstein A. R., Franzke Y. J., Lichtenberger N., Wilson R. J., Deubner H. L., Kraus F., Clérac R., Weigend F., Dehnen S. (2021). Nat. Chem..

[cit156] Pugliese E. R., Benner F., Demir S. (2023). Chem.–Eur. J..

[cit157] Pugliese E. R., Benner F., Demir S. (2023). Chem. Commun..

[cit158] Wen Q., Feng B., Chen Y. (2023). Acc. Chem. Res..

