# Correction: f-Element heavy pnictogen chemistry

**DOI:** 10.1039/d4sc90017k

**Published:** 2024-02-21

**Authors:** Jingzhen Du, Philip J. Cobb, Junru Ding, David P. Mills, Stephen T. Liddle

**Affiliations:** a College of Chemistry, Zhengzhou University Zhengzhou 450001 China; b Department of Chemistry and Centre for Radiochemistry Research, The University of Manchester Oxford Road Manchester M13 9PL UK david.mills@manchester.ac.uk steve.liddle@manchester.ac.uk

## Abstract

Correction for ‘f-Element heavy pnictogen chemistry’ by Jingzhen Du *et al.*, *Chem. Sci.*, 2024, **15**, 13–45, https://doi.org/10.1039/D3SC05056D.

The authors regret that ref. 57 is incomplete in the original article. Ref. 57 is not incorrect, but the associated complex was also published in a paper in *J. Am. Chem. Soc.*, where its synthesis and characterisation were reported as part of a reactivity study of complex **27Y** with unsaturated substrates. The corrected ref. 57 is shown below as ref. 1.

The Royal Society of Chemistry apologises for these errors and any consequent inconvenience to authors and readers.

## Supplementary Material
